# A Paternò–Büchi Reaction of Aromatics with Quinones under Visible Light Irradiation

**DOI:** 10.3390/molecules29071513

**Published:** 2024-03-28

**Authors:** Wen-Wen Li, Jia-Lin Zhao, Ze-Yu Wang, Pei-Ting Li, Zi-Fa Shi, Xiao-Ping Cao, Qiang Liu

**Affiliations:** State Key Laboratory of Applied Organic Chemistry and College of Chemistry & Chemical Engineering, Lanzhou University, Lanzhou 730000, China; liww21@stu.xjtu.edu.cn (W.-W.L.); jlzhao20@lzu.edu.cn (J.-L.Z.); zywang19@lzu.edu.cn (Z.-Y.W.); lipt19@lzu.edu.cn (P.-T.L.); caoxp@lzu.edu.cn (X.-P.C.)

**Keywords:** Paternò–Büchi reaction, oxetanes, diaryl ethers, photochemistry, synthetic methods

## Abstract

Reported herein is a Paternò–Büchi reaction of aromatic double bonds with quinones under visible light irradiation. The reactions of aromatics with quinones exposed to blue LED irradiation yielded oxetanes at −78 °C, which was attributed to both the activation of double bonds in aromatics and the stabilization of oxetanes by thiadiazole, oxadiazole, or selenadiazole groups. The addition of Cu(OTf)_2_ to the reaction system at room temperature resulted in the formation of diaryl ethers via the copper-catalyzed ring opening of oxetanes in situ. Notably, the substrate scope was extended to general aromatics.

## 1. Introduction

**Scheme 1:**
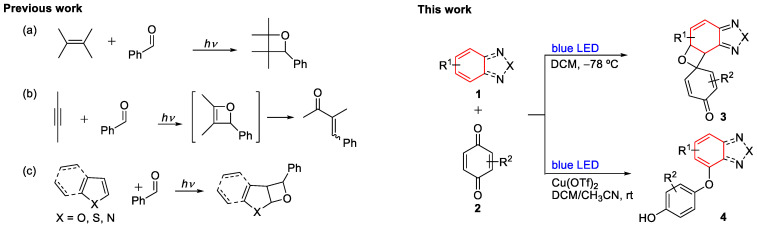
Selected examples of the Paternò–Büchi reaction.

Oxetane structural units are widely present in natural products and pharmaceutical active molecules [1,2,3,4,5]. The Paternò–Büchi (PB) reaction is an effective method to synthesize azetidines [6,7,8,9] and oxetanes [10,11,12,13]. The PB reaction originally referred to the reaction of olefins and carbonyl compounds under light irradiation to synthesize oxetanes (Scheme 1a) [14,15]. After decades of development, several carbon–carbon double bonds were subjected to the PB reaction with carbonyl compounds to synthesize oxetanes. Alkynes and carbonyl compounds yield unsaturated carbonyl compounds via oxetane intermediates (Scheme 1b) [16]. Furans [17,18], thiophenes [19,20,21], pyrroles [22,23], imidazoles [24,25], oxazoles [26], and indoles [27] also underwent the PB reaction with carbonyl under light conditions to generate oxetanes (Scheme 1c). The higher delocalization of large π-systems in the benzene ring than in the aromatic heterocyclic ring hinders addition reactions of aromatics, although they can be functionalized by radicals to synthesize phenols or arylamines [28,29]. To our knowledge, the PB reaction involving aromatic double bonds has not yet been reported, which facilitates the construction of oxa-[4.2.0] skeletons.

The vast majority of PB reactions require the use of UV light. A few visible-light PB reactions have recently been reported. In 2019, functionalized azetidines were synthesized via intramolecular aza PB reactions between imines and alkenes under visible light irradiation catalyzed by precious Ir-based complexes, as reported by Schindler and coworkers [30]. In 2020, Dell’Amico and coworkers developed a visible-light PB reaction of substituted indoles and aromatic ketones [27].

Herein, a PB reaction of aromatics with quinones was developed under visible light irradiation. At −78 °C, the [2+2] cycloadditions of carbon–carbon double bonds in the aromatics, which were activated by thiadiazole, oxadiazole, or selenadiazole, were achieved to form oxetanes with the carbonyl of quinones under blue LED irradiation (λ = 450 nm). The addition of Cu(OTf)_2_ at room temperature resulted in the synthesis of diaryl ethers from aromatics and quinones under the same irradiation via an oxetane intermediate. The range of substrate adaptation can be extended to general aromatics for the synthesis of diaryl ethers.

## 2. Results

Initially, 5,6-dimethylbenzothiadiazole (**1a**) and *p*-benzoquinone (**2a**) were selected as the model substrates. The solution of **1a** and **2a** with equivalent doses in CH_3_CN at 40 °C under blue LED (450 nm, 5 W) resulted in a 25% yield of oxetane **3a** (Table 1, entry 1). The ratio of **1a** and **2a** affected the yield of **3a** (Table 1, entries 2–4). A 3:1 ratio of **1a** and **2a** increased the yield of **3a** to 43% (Table 1, entry 4). Irradiation with UV light (λ = 254 nm) resulted in only a 7% yield of **3a**. Energy transfer catalysts can be used to promote [2+2] photocycloaddition [31]. The sensitizers matching the triplet excitation energy of **1a** (*E*_T_ = 46.1 kcal mol^−1^, calculated by DFT under B3LYP/6-31G(d)) or **2a** (*E*_T_ = 53.6 kcal mol^−1^) were used to improve the reaction yield, such as Ir(ppy)_3_ (*E*_T_ = 58.1 kcal mol^−1^), Ru(bpy)_3_Cl_2_·6H_2_O (*E*_T_ = 49.0 kcal mol^−1^), and thioxanthen-9-one (*E*_T_ = 63.4 kcal mol^−1^) [32]; however, they did not contribute to this reaction (Table 1, entries 6–8). Because product **3a** can be partially decomposed into starting materials during separation, the reaction was carried out at low temperatures to increase the yield. When the reaction was performed at –40 °C, the yield was increased to 48% (entry 9). At –78 °C, no oxetane **3a** was detected when Et_2_O or THF was used as a solvent (Table 1, entries 10 and 11). In dichloromethane (DCM), the optimal yield of **3a** was 74%. And, when the reaction was carried out in the dark at –78 °C, there was no **3a** detected. Thus, the use of **1a** (0.9 mmol) and **2a** (0.3 mmol) in DCM (3 mL) at –78 °C under blue LED irradiations represents optimized reaction conditions.

**Scheme 2:**
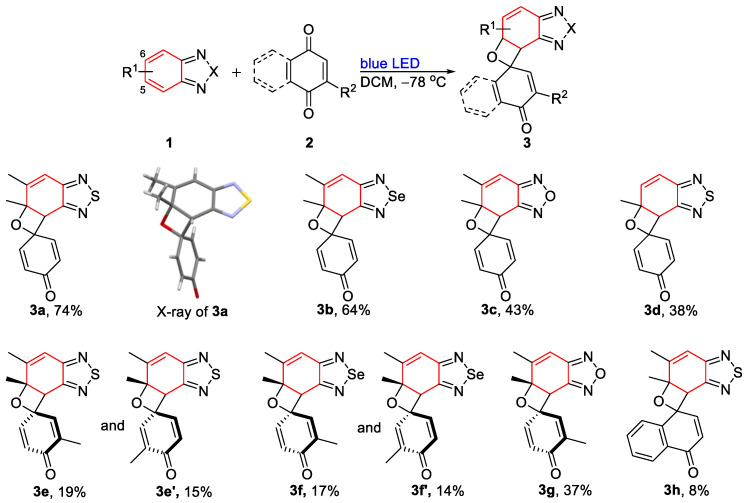
Substrate scope of the Paternò–Büchi reaction of aromatic with quinones.

Under the optimized reaction conditions, the substituent effect was significant and similar to many reported PB reactions [10]. The presence of methyl groups in **1** at position 5 or 6 facilitated the formation of oxetane products under the optimized conditions. As X in **1** was selenium or oxygen, oxetanes **3b** and **3c** were formed in 64% and 43% yields, respectively (Scheme 2). The presence of only a single methyl in 5-methylbenzo[*c*][1,2,5]thiadiazole (**1d**) at position 5 resulted in the selective synthesis of compound **3d** in 38% yield. The PB reaction only involved the double bonds with the methyl group in **1d**. The substrate 2-methylcyclohexa-2,5-diene-1,4-dione (**2b**) was also used in the PB reactions involving **1a**, 5,6-dimethylbenzo[*c*][1,2,5]selenadiazole (**1b**), or 5,6-dimethylbenzo[*c*][1,2,5]oxadiazole (**1c**) to synthesize oxetanes. Only the carbonyl at position 4 in **2b** participated in the PB reaction. The two isomers (**3e** and **3e′**, **3f** and **3f′**), whose configurations (**3e** and **3e′**) were confirmed via NOESY spectra (pages S16 and S18 in Supplementary Materials), were formed via the reaction of **2b** with **1a** and **1b**, respectively. However, only isomer **3g** was detected in the reaction between **2b** and **1c**. Naphthalene-1,4-dione (**1e**) was also used as a substrate in this PB reaction to obtain oxetane **3h** in 8% yield.

**Scheme 3:**
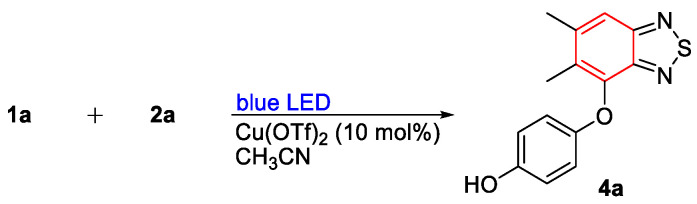
The reaction of **1a** and **2a** catalyzed by Cu(OTf)_2_.

At room temperature, the oxetane products in solution can be translated into the starting materials, e.g., a portion of **3a** was transformed into **1a** and **2a** in deuterated chloroform in 2 h. Thus, an equilibrium may exist between the oxetane products and the starting materials in the reactions under the optimized reaction conditions. In the case of the other substrates, the equilibrium shifted in favor of the starting materials, resulting in a low yield of oxetanes, which could not be separated. To expand the application of this reaction, Cu(OTf)_2_ was added to translate the oxetane to shift the equilibrium to the right by the ring-opening reaction of the oxetane catalyzed by Lewis acid [33,34]. Compound **4a** was obtained when the reaction of **1a**, **2a**, and Cu(OTf)_2_ (0.1 equiv.) in CH_3_CN was carried out under blue LED irradiation, which prompted us to develop a method for the synthesis of diaryl ethers (Scheme 3).

Diaryl ethers are important structural units, which are widely present in natural product molecules and functional materials [35,36]. Therefore, the development of effective methods for synthesizing diaryl ether has great research significance and widespread application. The general method to produce diaryl ethers involves the reaction of aryl halides with phenoxides via substitution reactions at high temperature in the presence of electron-withdrawing groups in aryl halides [37,38], and with phenols or phenoxides via transition metal-catalyzed reactions [39,40,41]. Methods for the synthesis of diaryl ethers also were developed without the use of aryl halides. The reactions involving phenols with aryl boronic acids in the presence of copper acetate were developed to synthesize diaryl ethers [42,43,44,45]. Recently, Ritter and coworkers developed a two-step process of diaryl ether synthesis from electron-rich aromatics and phenols using aryl thianthrenium salts by photoredox chemistry [46]. In this work, diaryl ethers were synthesized via a reaction between quinones and aromatics, which included electron-rich, -poor, and -neutral aromatics under blue LED in the presence of Cu(OTf)_2_.

To ensure optimized conditions, benzo[*c*][1,2,5]thiadiazole (**1b**) was used as the model substrate in the reaction with **2a** for conditional screening (Table 2). To our delight, the diaryl ether product **4b** was afforded in 50% yield using Cu(OTf)_2_ (10% of **2a**) as the catalyst in CH_3_CN at 40 °C under blue LED for 48 h (entry 1). Photosensitizers (Ir(bpy)_3_, Ru(bpy)_3_Cl_2_·6H_2_O, and thioxanthen-9-one) were also added to the reactions to increase the product yield, but there was no effect on the yield increase in **4a** (entries 2–4). The Lewis acids were selected as catalysts to optimize this reaction. When the Lewis acids, Fe(OTf)_3_, Zn(OTf)_2_, Yb(OTf)_3_, Nd(OTf)_3_, and Sc(OTf)_3_ (entries 5–9), were used to replace Cu(OTf)_2_ in the reactions, product **4a** could be obtained, but the yields were lower than that of entry 1. The using of Pd(OAc)_2_, AgOAc, and BF_3_·Et_2_O did not give **4a**. The product yield was also affected by reaction temperature. When the reaction was carried out at 25 °C, the yield of **4a** was increased to 53% (entry 13). The decrease in the ratio of **1b** and **2a** did not increase the yield of **4a** (entries 15–18). Under the 3:1 ratio of **1b** and **2a**, the yield of **4a** increased to 65% when the amount of Cu(OTf)_2_ increased to 20% of **2a** (entry 19). Then, the solvent effect was explored to increase the product yield. Product **4a** was not detected when the reactions were carried out in CH_3_OH, acetone, Et_2_O, or THF (entries 20–23). When DCM was used as a solvent, **4a** was obtained in 45% yield. Thus, a mixed solvent of DCM and CH_3_CN was used in the reaction, which resulted in the highest yield of **4a** (67%, entry 25). In addition, without blue LED irradiation, no **4a** was obtained (entry 26), which indicated that the excitation of the starting material was a decisive factor in the reaction rather than the copper-catalyzed coupling reaction. Finally, after the optimization of Lewis acids, reaction temperature, and solvents, **1b** (0.9 mmol), **2a** (0.3 mmol), and Cu(OTf)_2_ (0.06 mmol) in CH_3_CN/DCM (1:1, 3 mL) at room temperature for 48 h under blue LED were determined as the optimized conditions.

**Scheme 4:**
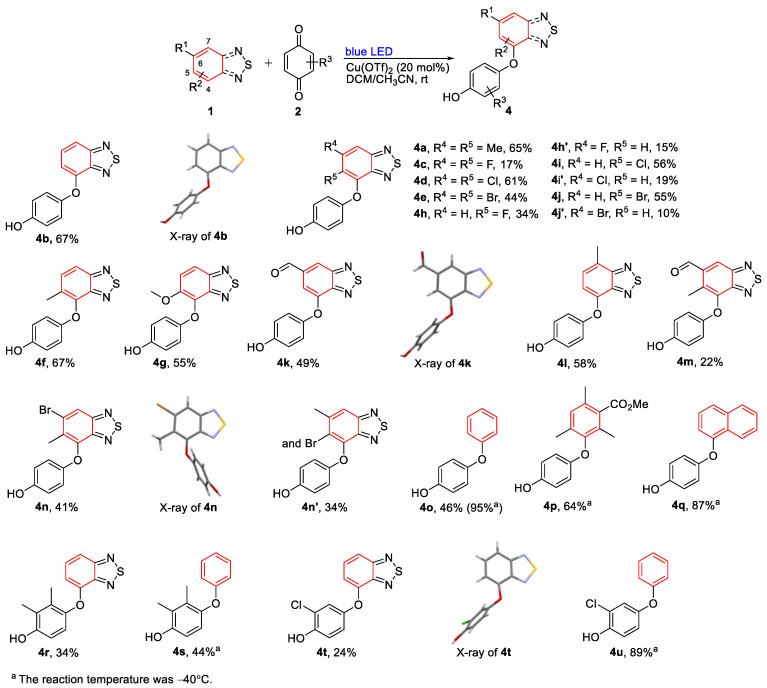
Substrate scope for the synthesis of diaryl ethers.

We then explored the scope of diaryl ether synthesis (Scheme 4). As substrate **1** contains thiadiazole, etherification always occurs at the active positions 4 or 7 in **1**. Under the optimized conditions, **4b** was obtained in 67% yield. The introduction of the same functional groups at positions 5 and 6 in **1**, such as CH_3_, F, Cl, and Br, resulted in diaryl ethers **4a** and **4c**–**4e** in good yield (besides **4c**). In the presence of a single substituent group in benzothiadiazole, the regioselectivity of the etherification was controlled by the electronic effects of the substituent. The presence of an electron-rich group (CH_3_, CH_3_O) at position 5 of **1** resulted in the synthesis of compounds **4f** and **4g** in 67% and 55% yield, respectively, via etherification at position 4 of **1**. With halogen at position 5 in **1**, two isomers of etherification at positions 4 and 7 were obtained, but the main products were **4h**, **4i**, and **4j**. When position 5 of **1** was substituted by the formyl group (electron-poor group), its etherification at position 4 produced **4k** in 49% yield. The presence of a CH_3_ group at position 4 in **1** led to its etherification at position 7 to generate **4l** in 58% yield. The reaction of 6-methylbenzo[*c*][1,2,5]thiadiazole-5-carbaldehyde (**1m**, R^1^ = 5-CHO, R^2^ = 6-CH_3_) and **2a** afforded **4m** in low yield. However, 5-bromo-6-methylbenzo[*c*][1,2,5]thiadiazole (**1n**, R^1^ = 5-Br, R^2^ = 6-CH_3_) reacted smoothly with **2a** under standard conditions to generate two isomers **4n** and **4n’** in good yields. General aromatics without the activating group (thiadiazole) were also used in this reaction to afford the corresponding diaryl ethers. Benzene as a substrate reacted with benzoquinone under standard conditions to yield **4o** (46%). Reducing the reaction temperature to −40 °C resulted in a 95% yield of **4o** (Table S1). The synthesis of **4p**, **4q**, **4s**, and **4u** was carried out in the presence of Cu(OTf)_2_ in CH_3_CN/DCM (1:1) at −40 °C for 48 h. When methyl 2,4,6-trimethylbenzoate was used as the substrate, the corresponding product **4p** was obtained in 64% yield, although it had steric hindrance. Etherification at the α position of naphthalene afforded **4q** in 87% yield. The substituent quinones also were tolerated in this reaction. The reactions of 2,3-dimethylbenzoquinone (**2c**, R^3^ = 2,3-dimethyl) with **1b** and benzene were carried out to obtain **4r** and **4s** with a yield of 34% and 44%, respectively. 2-Chlorobenzoquinone (**2d**, R^3^ = 2-Cl) reacted with **1b** to afford **4t** in 24% yield but with benzene to yield **4u** in good yield (89%).

The products all were characterized by ^1^H NMR, ^13^C NMR, and HRMS. The configurations of products were confirmed by X-ray analysis of the single crystals of **3a**, **4b**, **4k**, **4n**, and **4t**.

**Scheme 5:**
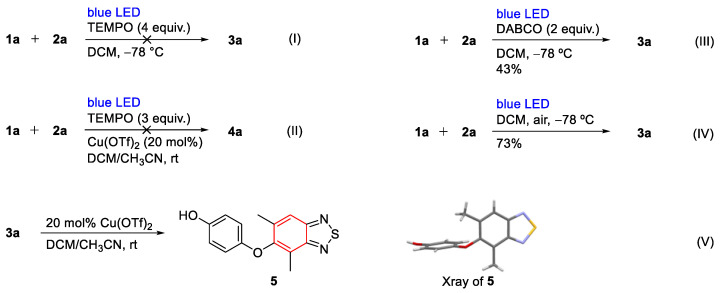
Control experiments.

To elucidate the reaction mechanisms, the control experiments, UV-vis absorption, and fluorescence involved **1a** and **2a** were investigated. In UV-vis absorption spectra (Figure S1), only benzoquinone (**2a**) had an absorption peak at >400 nm, which was not affected by **1a** or Cu(OTf)_2_. As shown in Scheme 5, no products were detected when 2,2,6,6-tetramethylpiperidinooxy (TEMPO) was added to the reactions during the synthesis of oxetane **3a** and diaryl ether **4a** under standard conditions (I and II in Scheme 5). The results showed that **2a** was excited under blue LED and the processes involved free radicals. Product **3a** could be smoothly obtained in the reaction with triplet quencher DABCO (III in Scheme 5), or the reaction under atmosphere (IV in Scheme 5) [47], which indicated that the triplet state was not favored in the reaction system. The result of the energy transfer calculations from the Gibbs energy of the photoinduced electron transfer (PET) equation showed Δ*G* > 0 (page S51 in Supplementary Materials), forbidding the possibility of the PET mechanism, which also needs to involve the triplet state [47]. Oxetane **3a** was treated with Cu(OTf)_2_ (0.2 equiv.) in DCM/CH_3_CN (1:1) at room temperature to obtain compound **5**, whose structure was confirmed via X-ray analysis of its single crystal (V in Scheme 5).

**Scheme 6:**
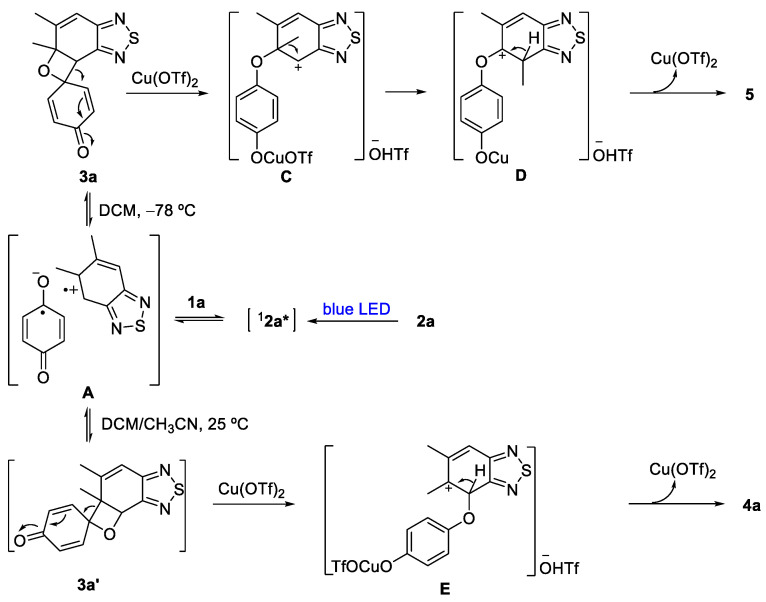
Speculated mechanism.

Based on the literature [13,48] and the experimental evidence, a plausible mechanism of the reactions involving **1a** and **2a** was proposed (Scheme 6). Under blue LED irradiation, **2a** was excited to the singlet state and reacted with **1a** to mainly form the singlet exciplex (**A**) [49]. The two oxetane products (**3a** and **3a′**) may be produced via singlet biradical intermediates, which could go back to **1a** and **2a**. Oxetane **3a** was more stable than **3a′** (the internal energy difference was 8 kcal/mol, which was calculated by DFT at the B3LYP/6-31G* level), which can be separated and characterized. Treatment of **3a** with Cu(OTf)_2_ resulted in the cleavage of the carbon–carbon bond in oxetane to form intermediate **C**, followed by methyl migration and aromatization resulting in **5**. When the starting materials were treated with Cu(OTf)_2_ under blue LED irradiation at room temperature, the equilibrium was biased toward raw materials. However, the highly reactive intermediate **3a′** was transformed into intermediate **E**, followed by aromatization to form **4a**, which eventually promoted the transformation of starting materials to **4a**. Accordingly, only **4a** was obtained instead of **5** because of the high energy barrier from **C** to **D**.

## 3. Experimental Section

### 3.1. Synthetic Procedures

#### 3.1.1. General Process I: Synthesis of Oxetanes (**3a**–**3h**)

To a quartz tube containing a stirring bar, aromatic **1** (0.9 mmol), quinone **2** (0.3 mmol), and dichloromethane (3 mL) were added. The reaction tube was degassed with argon and stirred for 48 h at –78 °C under blue LED (λ = 450 nm, 5 W) irradiation. The reaction system was raised to room temperature and transferred into a single-necked flask. The reaction mixture was concentrated in vacuo and separated by column chromatography (PE/EtOAc = 5:1) to obtain **3**.

*5′,5a′-Dimethyl-5a′,7a′-dihydrospiro[cyclohexane-1,7′-oxeto[3′,2′:3,4]benzo[1,2-c][1,2,5]thiadiazole]-2,5-dien-4-one* (**3a**). Colorless solid (61 mg, 74%); M.p. 108–110 °C; ^1^H NMR (600 MHz, CDCl_3_) *δ* 7.16 (dd, *J* = 10.1 Hz, 3.0 Hz, 1H), 6.84 (dd, *J* = 10.2 Hz, 2.9 Hz, 1H), 6.64 (s, 1H), 6.21 (d, *J* = 10.0 Hz, 1H), 5.98 (d, *J* = 10.3 Hz, 1H), 4.44 (s, 1H), 2.06 (s, 3H), 1.84 (d, *J* = 0.7 Hz, 3H); ^13^C NMR (150 MHz, CDCl_3_) *δ* 184.2 (C), 155.7 (C), 153.5 (C), 147.7 (CH), 146.7 (C), 144.6 (CH), 129.7 (CH), 128.4 (CH), 118.3 (CH), 82.6 (C), 78.8 (C), 50.6 (CH), 28.8 (CH_3_), 16.6 (CH_3_); HRMS (ESI) *m*/*z* calcd for C_14_H_13_N_2_O_2_S [M + H]^+^: 273.0692, found: 273.0694.
*5′,5a′-Dimethyl-5a′,7a′-dihydrospiro[cyclohexane-1,7′-oxeto[3′,2′:3,4]benzo[1,2-c][1,2,5]selenadiazole]-2,5-dien-4-one* (**3b**). Colorless solid (61 mg, 64%); M.p. 107–109 °C; ^1^H NMR (400 MHz, CDCl_3_) *δ* 7.15 (dd, *J* = 10.0 Hz, 3.2 Hz, 1H), 6.84 (dd, *J* = 10.0 Hz, 3.2 Hz, 1H), 6.65 (d, *J* = 1.6 Hz, 1H), 6.20 (dd, *J* = 10.0 Hz, 2.0 Hz, 1H), 5.97 (dd, *J* = 10.4 Hz, 2.0 Hz, 1H), 4.43 (s, 1H), 2.04 (d, *J* = 1.6 Hz, 3H), 1.82 (s, 3H); ^13^C NMR (100 MHz, CDCl_3_) *δ* 184.4 (C), 159.7 (C), 158.4 (C), 148.0 (CH), 147.3 (C), 144.7 (CH), 130.0 (CH), 128.7 (CH), 122.0 (CH), 82.8 (C), 78.7 (C), 53.7 (CH), 28.7 (CH_3_), 16.7 (CH_3_); HRMS (ESI) *m*/*z* calcd for C_14_H_13_N_2_O_2_Se [M + H]^+^: 321.0137, found: 321.0135.
*5′,5a′-Dimethyl-5a′,7a′-dihydrospiro[cyclohexane-1,7′-oxeto[3′,2′:3,4]benzo[1,2-c][1,2,5]oxadiazole]-2,5-dien-4-one* (**3c**). Colorless solid (33 mg, 43%); M.p. 113–115 °C; ^1^H NMR (400 MHz, CDCl_3_) *δ* 7.15 (dd, *J* = 10.1 Hz, 2.8 Hz, 1H), 6.75 (dd, *J* = 10.4 Hz, 3.2 Hz, 1H), 6.62 (d, *J* = 1.6 Hz, 1H), 6.21 (dd, *J* = 10.2 Hz, 2.0 Hz, 1H), 6.05 (dd, *J* = 10.0 Hz, 2.0 Hz, 1H), 4.46 (s, 1H), 2.07 (d, *J* = 1.6 Hz, 3H), 1.81 (s, 3H); ^13^C NMR (100 MHz, CDCl_3_) *δ* 184.0 (C), 149.8 (C), 148.8 (C), 147.1 (CH), 146.9 (C), 143.6 (CH), 130.4 (CH), 129.1 (CH), 110.4 (CH), 81.1 (C), 77.6 (C), 44.4 (CH), 28.5 (CH_3_), 17.2 (CH_3_); HRMS (ESI) *m*/*z* calcd for C_14_H_13_N_2_O_3_ [M + H]^+^: 257.0921, found: 257.0920.
*5a′-Methyl-5a′,7a′-dihydrospiro[cyclohexane-1,7′-oxeto[3′,2′:3,4]benzo[1,2-c][1,2,5]thiadiazole]-2,5-dien-4-one* (**3d**). Light-yellow liquid (30 mg, 38%); ^1^H NMR (400 MHz, CDCl_3_) *δ* 7.18 (dd, *J* = 10.0 Hz, 3.2 Hz, 1H), 6.87 (d, *J* = 10.0 Hz, 1H), 6.76 (dd, *J* = 10.0 Hz, 2.8 Hz, 1H), 6.29 (d, *J* = 10.0 Hz, 1H), 6.20 (dd, *J* = 10.0 Hz, 2.0 Hz, 1H), 5.97 (dd, *J* = 10.4 Hz, 2.0 Hz, 1H), 4.46 (s, 1H), 1.80 (s, 3H); ^13^C NMR (100 MHz, CDCl_3_) *δ* 184.3 (C), 154.8 (C), 154.2 (C), 147.6 (CH), 144.6 (CH), 137.5 (CH), 129.9 (CH), 128.4 (CH), 121.4 (CH), 80.9 (C), 79.8 (C), 49.9 (CH), 30.3 (CH_3_); HRMS (ESI) *m*/*z* calcd for C_13_H_11_N_2_O_2_S [M + H]^+^: 259.0536, found: 259.0535.
*3,5′,5a′-Trimethyl-5a′,7a′-dihydrospiro[cyclohexane-1,7′-oxeto[3′,2′:3,4]benzo[1,2-c][1,2,5]thiadiazole]-2,5-dien-4-one* (**3e**). Light-yellow liquid (13 mg, 15%); ^1^H NMR (400 MHz, CDCl_3_) *δ* 6.93 (d, *J* = 1.6 Hz, 1H), 6.79 (dd, *J* = 10.0 Hz, 2.8 Hz, 1H), 6.63 (d, *J* = 1.2 Hz, 1H), 5.96 (d, *J* = 10.4 Hz, 1H), 4.42 (s, 1H), 2.05 (d, *J* = 1.6 Hz, 3H), 1.93 (d, *J* = 1.6 Hz, 3H), 1.82 (s, 3H); ^13^C NMR (100 MHz, CDCl_3_) *δ* 185.2 (C), 155.9 (C), 154.0 (C), 147.1 (CH), 144.3 (C), 143.4 (CH), 135.9 (C), 129.9 (CH), 118.3 (CH), 82.3 (C), 79.2 (C), 50.9 (CH), 29.0 (CH_3_), 16.8 (CH_3_), 15.5 (CH_3_); HRMS (ESI) *m*/*z* calcd for C_15_H_15_N_2_O_2_S [M + H]^+^: 287.0849, found: 287.0847.
*3,5′,5a′-Trimethyl-5a′,7a′-dihydrospiro[cyclohexane-1,7′-oxeto[3′,2′:3,4]benzo[1,2-c][1,2,5]thiadiazole]-2,5-dien-4-one* (**3e’**). Light-yellow liquid (16 mg, 19%); ^1^H NMR (400 MHz, CDCl_3_) *δ* 7.12 (dd, *J* = 10.0 Hz, 3.2 Hz, 1H), 6.65 (d, *J* = 1.6 Hz, 1H), 6.56 (d, *J* = 1.6 Hz, 1H), 6.20 (d, *J* = 10.0 Hz, 1H), 4.41 (s, 1H), 2.06 (d, *J* = 1.2 Hz, 3H), 1.82 (s, 3H), 1.67 (d, *J* = 1.2 Hz, 3H); ^13^C NMR (100 MHz, CDCl_3_) *δ* 185.1 (C), 155.9 (C), 154.0 (C), 147.6 (CH), 147.0 (C), 139.5 (CH), 137.2 (C), 128.5 (CH), 118.4 (CH), 82.4 (C), 79.4 (C), 50.7 (CH), 29.0 (CH_3_), 16.8 (CH_3_), 15.6 (CH_3_); HRMS (ESI) *m*/*z* calcd for C_15_H_15_N_2_O_2_S [M + H]^+^: 287.0849, found: 287.0847.
*3,5′,5a′-Trimethyl-5a′,7a′-dihydrospiro[cyclohexane-1,7′-oxeto[3′,2′:3,4]benzo[1,2-c][1,2,5]thiadiazole]-2,5-dien-4-one* (**3f**). Light-yellow liquid (17 mg, 17%); ^1^H NMR (400 MHz, CDCl_3_) *δ* 6.94 (dd, *J* = 3.2 Hz, 1.6 Hz, 1H), 6.82 (dd, *J* = 10.0 Hz, 3.2 Hz, 1H), 6.66 (d, *J* = 1.2 Hz, 1H), 5.97 (d, *J* = 10.0 Hz, 1H), 4.41 (s, 1H), 2.055 (d, *J* = 1.6 Hz, 3H), 1.935 (d, *J* = 1.2 Hz, 3H), 1.82 (s, 3H); ^13^C NMR (100 MHz, CDCl_3_) *δ* 185.1 (C), 159.8 (C), 158.7 (C), 147.6 (CH), 144.3 (CH), 143.6 (CH), 135.8 (C), 129.9 (CH), 121.8 (CH), 82.2 (C), 79.0 (C), 53.8 (CH), 28.7 (CH_3_), 16.7 (CH_3_), 15.5 (CH_3_); HRMS (ESI) *m*/*z* calcd for C_15_H_15_N_2_O_2_Se [M + H]^+^: 335.0293, found: 335.0293.
*3,5′,5a′-Trimethyl-5a′,7a′-dihydrospiro[cyclohexane-1,7′-oxeto[3′,2′:3,4]benzo[1,2-c][1,2,5]thiadiazole]-2,5-dien-4-one* (**3f′**). Light-yellow liquid (14 mg, 14%); ^1^H NMR (400 MHz, CDCl_3_) ^1^H NMR (400 MHz, CDCl_3_) *δ* 7.13 (dd, *J* = 10.0 Hz, 2.8 Hz, 1H), 6.68 (d, *J* = 1.2 Hz, 1H), 6.58 (dd, *J* = 2.8 Hz, 1.6 Hz, 1H), 6.21 (d, *J* = 10.0 Hz, 1H), 4.41 (s, 1H), 2.06 (d, *J* = 0.8 Hz, 3H), 1.82 (d, 3H), 1.67 (d, *J* = 1.2 Hz, 3H); ^13^C NMR (100 MHz, CDCl_3_) *δ* 185.1 (C), 159.9 (C), 158.9 (C), 147.9 (CH), 147.4 (CH), 139.6 (CH), 137.2 (C), 128.5 (CH), 121.9 (CH), 82.4 (C), 79.2 (C), 53.5 (CH), 28.8 (CH_3_), 16.7 (CH_3_), 15.6 (CH_3_); HRMS (ESI) *m*/*z* calcd for C_15_H_15_N_2_O_2_Se [M + H]^+^: 335.0293, found: 335.0294.
*3,5′,5a′-Trimethyl-5a′,7a′-dihydrospiro[cyclohexane-1,7′-oxeto[3′,2′:3,4]benzo[1,2-c][1,2,5]thiadiazole]-2,5-dien-4-one* (**3g**). Light-yellow liquid (30 mg, 39%); ^1^H NMR (400 MHz, CDCl_3_) *δ* 6.92 (d, *J* = 1.2 Hz, 1H), *δ* 6.72 (dd, *J* = 10.0 Hz, 2.8 Hz, 1H), 6.63 (s, 1H), 6.05 (d, *J* = 10.4 Hz, 1H), 4.44 (s, 1H), 2.07 (d, *J* = 1.3 Hz, 3H), 1.93 (s, 3H), 1.81 (s, 3H); ^13^C NMR (100 MHz, CDCl_3_) *δ* 184.8 (C), 15.0 (C), 148.9 (C), 147.2 (CH), 143.2 (C), 142.6 (CH), 136.5 (C), 130.4 (CH), 110.4 (CH), 80.7 (C), 78.1 (C), 44.7 (CH), 28.6 (CH_3_), 17.2 (CH_3_), 15.5 (CH_3_); HRMS (ESI) *m*/*z* calcd for C_15_H_14_N_2_NaO_3_ [M + Na]^+^: 293.0897, found: 293.0896.
*5′,5a′-Dimethyl-5a′,7a′-dihydro-4H-spiro[naphthalene-1,7′-oxeto[3′,2′:3,4]benzo[1,2-c][1,2,5]thiadiazol]-4-one* (**3h**). Light-brown solid (8 mg, 8%); M.p. 119–121 °C; ^1^H NMR (400 MHz, CDCl_3_) *δ* 8.16 (d, *J* = 8.0 Hz, 1H), 8.08 (d, *J* = 8.0 Hz 1H), 7.77 (t, *J* = 7.6 Hz, 1H), 7.54 (t, *J* = 7.6 Hz, 1H), 7.24 (d, *J* = 10.4 Hz, 1H), 6.69 (s, 1H), 6.11 (d, *J* = 10.4 Hz, 1H), 4.57 (s, 1H), 2.16 (s, 3H), 1.97 (s, 3H); ^13^C NMR (100 MHz, CDCl_3_) *δ* 183.3 (C), 155.8 (C), 153.8 (C), 147.4 (C), 146.3 (CH), 143.5 (C), 133.6 (CH), 130.0 (CH), 129.8 (C), 129.1 (CH), 126.7 (CH), 126.4 (CH), 118.4 (CH), 82.2 (C), 81.3 (C), 56.4 (CH), 27.7 (CH_3_), 16.8 (CH_3_); HRMS (ESI) *m*/*z* calcd for C_18_H_15_N_2_O_2_S [M + H]^+^: 323.0849, found: 323.0847.

#### 3.1.2. General Process II: Synthesis of Diaryl Ethers (**4a**–**4n**)

To a quartz tube containing a stirring bar, aromatic **1** (0.9 mmol), quinone **2** (0.3 mmol), copper trifluoromethanesulfonate (22 mg, 0.06 mmol), dichloromethane (1.5 mL), and acetonitrile (1.5 mL) were added. The reaction tube was degassed with argon and stirred for 48 h at room temperature under blue LED (λ = 450 nm, 5 W) irradiation. The reaction system was transferred into a single-necked flask. The reaction mixture was concentrated in vacuo and separated by column chromatography (PE/EtOAc = 10:1) to obtain **4**.

*4-((5,6-Dimethylbenzo[c][1,2,5]thiadiazol-4-yl)oxy)phenol* (**4a**). Light-yellow solid (53 mg, 65%); M.p. 187–188 °C; ^1^H NMR (400 MHz, CD_3_COCD_3_) *δ* 8.05 (s, 1H), 7.71 (s, 1H), 6.76–6.71 (m, 4H), 2.523 (d, *J* = 0.8 Hz, 3H), 2.33 (s, 3H); ^13^C NMR (100 MHz, CD_3_COCD_3_) *δ* 156.1 (C), 153.3 (C), 152.4 (C), 149.7 (C), 143.8 (C), 143.0 (C), 132.0 (C), 117.5 (CH), 117.2 (CH), 116.7 (CH), 21.2 (CH_3_), 12.9 (CH_3_); HRMS (ESI) *m*/*z* calcd for C_14_H_13_N_2_O_2_S [M + H]^+^: 273.0692, found: 273.0692.
*4-(Benzo[c][1,2,5]thiadiazol-4-yloxy)phenol* (**4b**). Green solid (49 mg, 67%); M.P. 178–179 °C; ^1^H NMR (400 MHz, CD_3_COCD_3_) *δ* 8.43 (s, 1H), 7.67 (d, *J* = 8.8 Hz, 1H), 7.60–7.56 (m, 1H), 7.09–7.07 (m, 2H), 6.95–6.93 (m, 2H), 6.77 (d, *J* = 7.6 Hz, 1H); ^13^C NMR (100 MHz, CD_3_COCD_3_) *δ* 157.6 (C), 155.6 (C), 151.9 (C), 149.1 (C), 148.6 (C), 131.2 (CH), 122.4 (CH), 117.3 (CH), 115.5 (CH), 111.6 (CH); HRMS (ESI) *m*/*z* calcd for C_12_H_9_N_2_O_2_S [M + H]^+^: 245.0379, found: 245.0378.
*4-((5,6-Difluorobenzo[c][1,2,5]thiadiazol-4-yl)oxy)phenol* (**4c**). Colorless solid (14 mg, 17%); M.P. 168–169 °C; ^1^H NMR (400 MHz, CD_3_COCD_3_) *δ* 8.23 (s, 1H), 7.90–7.85 (m, 1H), 6.99 (d, *J* = 12.8 Hz, 2H), 6.80 (d, *J* = 10.8 Hz, 2H); ^13^C NMR (100 MHz, CD_3_COCD_3_) *δ* 155.5 (C, dd, *J* = 253.0 Hz, 16.0 Hz), 154.6 (C), 151.7 (C), 151.5 (C, d, *J* = 14.0 Hz), 148.0 (C), 146.5 (C, dd, *J* = 256.0 Hz, 20.0 Hz), 133.5 (C, d, *J* = 16.0 Hz), 118.5 (CH), 116.8 (CH), 103.0 (CH, d, *J* = 21.0 Hz); ^19^F NMR (100 MHz, CD_3_COCD_3_) *δ* −129.7 (F, dd, *J* = 4.6 Hz, 2.7 Hz), −149.8 (F, dd, *J* = 4.5 Hz, 1.8 Hz); HRMS (ESI) *m*/*z* calcd for C_12_H_7_F_2_N_2_O_2_S [M + H]^+^: 281.0191, found: 281.0190.
*4-((5,6-Dichlorobenzo[c][1,2,5]thiadiazol-4-yl)oxy)phenol* (**4d**). Green solid (57 mg, 61%); M.P. 182–183 °C; ^1^H NMR (400 MHz, CD_3_COCD_3_) *δ* 8.25 (s, 1H), 8.22 (s, 1H), 6.88 (d, *J* = 8.8 Hz, 2H), 6.78 (d, *J* = 9.2 Hz, 2H); ^13^C NMR (100 MHz, CD_3_COCD_3_) *δ* 154.7 (C), 154.2 (C), 151.4 (C), 149.2 (C), 144.7 (C), 135.8 (C), 127.0 (C), 118.8 (CH), 118.1 (CH), 116.7 (CH); HRMS (ESI) *m*/*z* calcd for C_12_H_7_Cl_2_N_2_O_2_S [M + H]^+^: 312.9600, found: 312.9591.
*4-((5,6-Dibromobenzo[c][1,2,5]thiadiazol-4-yl)oxy)phenol* (**4e**). Yellow solid (53 mg, 44%); M.P. 183–184 °C; ^1^H NMR (400 MHz, CD_3_COCD_3_) *δ* 8.41 (s, 1H), 8.21 (s, 1H), 6.85 (d, *J* = 9.2 Hz, 2H), 6.77 (d, *J* = 8.8 Hz, 2H); ^13^C NMR (100 MHz, CD_3_COCD_3_) *δ* 156.0 (C), 154.2 (C), 151.4 (C), 149.4 (C), 146.0 (C), 127.9 (C), 122.3 (CH), 120.5 (C), 118.1 (CH), 116.7 (CH); HRMS (ESI) *m*/*z* calcd for C_12_H_7_Br_2_N_2_O_2_S [M + H]^+^: 402.8569, found: 402.8567.
*4-((5-Methylbenzo[c][1,2,5]thiadiazol-4-yl)oxy)phenol* (**4f**). Colorless solid (52 mg, 67%); M.P. 133–134 °C; ^1^H NMR (400 MHz, CDCl_3_), *δ* 7.79 (d, *J* = 8.8 Hz, 1H), 7.53 (d, *J* = 9.2 Hz, 1H), 6.76–6.71 (m, 4H), 5.05 (s, 1H) 2.41 (s, 3H); ^13^C NMR (100 MHz, CDCl_3_) *δ* 155.5 (C), 152.0 (C), 150.7 (C), 149.9 (C), 143.1 (C), 133.8 (CH), 130.4 (C), 117.6 (CH), 116.5 (CH), 116.2 (CH), 15.8 (CH_3_); HRMS (ESI) *m*/*z* calcd for C_13_H_11_N_2_O_2_S [M + H]^+^: 259.0536, found: 259.0534.
*4-((5-Methoxybenzo[c][1,2,5]thiadiazol-4-yl)oxy)phenol* (**4g**). Green solid (45 mg, 55%); M.P. 149–150 °C; ^1^H NMR (400 MHz, CDCl_3_), *δ* 7.85 (d, *J* = 9.2 Hz, 1H), 7.56 (d, *J* = 9.6 Hz, 1H), 6.86–6.82 (m, 2H), 6.75–6.71 (m, 2H), 4.01 (s, 3H); ^13^C NMR (100 MHz, CDCl_3_) *δ* 152.2 (C), 152.1 (C), 151.5 (C), 150.7 (C), 132.8 (C), 121.9 (CH), 118.0 (CH), 116.7 (CH), 116.1 (CH), 58.3 (CH_3_); HRMS (ESI) *m*/*z* calcd for C_13_H_11_N_2_O_3_S [M + H]^+^: 275.0485, found: 275.0483.
*4-((5-Fluorobenzo[c][1,2,5]thiadiazol-4-yl)oxy)phenol* (**4h**). Yellow solid (27 mg, 34%); M.P. 159–160 °C; ^1^H NMR (400 MHz, CD_3_COCD_3_), *δ* 8.20 (s, 1H), 7.96 (dd, *J* = 9.2 Hz, 4.4 Hz, 1H), 7.78 (dd, *J* = 10.8 Hz, 10.0 Hz, 1H), 6.94–6.90 (m, 2H), 6.81–6.77 (m, 2H); ^13^C NMR (100 MHz, CD_3_COCD_3_) *δ* 155.4 (C, d, *J* = 249.0 Hz), 154.2 (C), 154.1 (C), 152.1 (C), 151.3 (C, d, *J* = 7.0 Hz), 133.5 (C, d, *J* = 14.0 Hz), 122.9 (CH, d, *J* = 26.0 Hz), 119.0 (CH, d, *J* = 10.0 Hz), 118.0 (CH), 116.7 (CH); ^19^F NMR (100 MHz, CD_3_COCD_3_) *δ* −130.8 (F, d, *J* = 2.0 Hz); HRMS (ESI) *m*/*z* calcd for C_12_H_8_FN_2_O_2_S [M + H]^+^: 263.0285, found: 263.0284.
*4-((6-Fluorobenzo[c][1,2,5]thiadiazol-4-yl)oxy)phenol* (**4h′**). Colorless solid (12 mg, 15%); M.P. 200–202 °C; ^1^H NMR (400 MHz, CD_3_COCD_3_), *δ* 8.59 (s, 1H), 7.38 (dd, *J* = 9.2 Hz, 2.4 Hz, 1H), 7.17–7.15 (m, 2H), 7.00–6.98 (m, 2H), 6.57 (dd, *J* = 11.2 Hz, 2.4 Hz, 1H); ^13^C NMR (100 MHz, CD_3_COCD_3_) *δ* 165.4 (C, d, *J* = 248.0 Hz), 156.7 (C, d, *J* = 14.0 Hz), 156.3 (C), 152.8 (C, d, *J* = 15.0 Hz), 147.7 (C), 146.7 (C), 122.8 (CH), 117.6 (CH), 102.9 (d, *J* = 34.0 Hz, CH), 98.7 (d, *J* = 25.0 Hz, CH); ^19^F NMR (100 MHz, CD_3_COCD_3_) *δ* −108.4 (F); HRMS (ESI) *m*/*z* calcd for C_12_H_8_FN_2_O_2_S [M + H]^+^: 263.0285, found: 263.0284.
*4-((5-Chlorobenzo[c][1,2,5]thiadiazol-4-yl)oxy)phenol* (**4i**). Green solid (47 mg, 56%); M.P. 154–156 °C; ^1^H NMR (400 MHz, CD_3_COCD_3_), *δ* 8.13 (s, 1H), 7.94 (d, *J* = 9.2 Hz, 1H), 7.82 (d, *J* = 9.2 Hz, 1H), 6.86–6.82 (m, 2H), 6.79–6.75 (m, 2H); ^13^C NMR (100 MHz, CD_3_COCD_3_) *δ* 156.4 (C), 154.7 (C), 151.8 (C), 150.9 (C), 143.8 (C), 132.5 (CH), 127.6 (C), 119.3 (CH), 118.1 (CH), 116.8 (CH); HRMS (ESI) *m*/*z* calcd for C_12_H_8_ClN_2_O_2_S [M + H]^+^: 278.9990, found: 278.9987.
*4-((6-Chlorobenzo[c][1,2,5]thiadiazol-4-yl)oxy)phenol* (**4i′**). Green solid (16 mg, 19%); M.P. 160–162 °C; ^1^H NMR (400 MHz, CD_3_COCD_3_), *δ* 8.59 (s, 1H), 7.73 (d, *J* = 2 Hz, 1H), 7.18–7.13 (m, 2H), 7.00–6.97 (m, 2H), 6.64 (d, *J* = 2 Hz, 1H); ^13^C NMR (100 MHz, CD_3_COCD_3_) *δ* 156.9 (C), 156.2 (C), 152.2 (C), 147.8 (C), 147.7 (C), 137.3 (C), 122.7 (CH), 117.6 (CH), 114.3 (CH), 112.3 (CH); HRMS (ESI) *m*/*z* calcd for C_12_H_8_ClN_2_O_2_S [M + H]^+^: 278.9990, found: 278.9987.
*4-((5-Bromobenzo[c][1,2,5]thiadiazol-4-yl)oxy)phenol* (**4j**). Brown solid (53 mg, 55%); M.P. 174–175 °C; ^1^H NMR (400 MHz, CD_3_COCD_3_), *δ* 8.17 (s, 1H), 7.95 (d, *J* = 9.2 Hz, 1H), 7.88 (d, *J* = 9.2 Hz, 1H), 6.85–6.81 (m, 2H), 6.79–6.75 (m, 2H); ^13^C NMR (100 MHz, CD_3_COCD_3_) *δ* 156.8 (C), 154.0 (C), 151.7 (C), 150.8 (C), 145.2 (C), 134.9 (CH), 119.5 (CH), 118.0 (CH), 117.1 (C), 116.7 (CH); HRMS (ESI) *m*/*z* calcd for C_12_H_8_BrN_2_O_2_S [M + H]^+^: 322.9484, found: 322.9483.
*4-((6-Bromobenzo[c][1,2,5]thiadiazol-4-yl)oxy)phenol* (**4j′**). Light-yellow solid (10 mg, 10%); M.P. 173–174 °C; ^1^H NMR (400 MHz, CD_3_COCD_3_), *δ* 8.61 (s, 1H), 7.926 (d, *J* = 1.6 Hz, 1H), 7.18–7.14 (m, 2H), 7.01–6.97 (m, 2H), 6.748 (d, *J* = 1.6 Hz, 1H); ^13^C NMR (100 MHz, CD_3_COCD_3_) *δ* 157.5 (C), 156.2 (C), 152.2 (C), 148.0 (C), 147.7 (C), 125.2 (C), 122.8 (CH), 117.7 (CH), 117.6 (CH), 114.6 (CH); HRMS (ESI) *m*/*z* calcd for C_12_H_8_BrN_2_O_2_S [M + H]^+^: 322.9484, found: 322.9483.
*7-(4-Hydroxyphenoxy)benzo[c][1,2,5]thiadiazole-5-carbaldehyde* (**4k**). Brown solid (40 mg, 49%); M.P. 199–201 °C; ^1^H NMR (400 MHz, CD_3_COCD_3_); *δ* 10.16 (s, 1H), 8.54 (s, 1H), 8.364 (d, *J* = 1.2 Hz, 1H), 7.17–7.13 (m, 2H), 7.105 (d, *J* = 1.2 Hz, 1H), 7.01–6.97 (m, 2H); ^13^C NMR (100 MHz, CD_3_COCD_3_) *δ* 192.3 (CH) 157.2 (C), 156.1 (C), 153.0 (C), 151.4 (C), 147.8 (C), 139.4 (C), 122.8 (CH), 122.3 (CH), 117.5 (CH), 105.8 (CH); HRMS (ESI) *m*/*z* calcd for C_13_H_9_N_2_O_3_S [M + H]^+^: 273.0328, found: 273.0327.
*4-((7-Methylbenzo[c][1,2,5]thiadiazol-4-yl)oxy)phenol* (**4l**). Yellow solid (45 mg, 58%); M.p. 204–206 °C; ^1^H NMR (400 MHz, CD_3_COCD_3_); *δ* 8.37 (s, 1H), 7.34 (d, *J* = 6.4 Hz, 1H), 7.04 (dd, *J* = 6.4 Hz, 2 Hz, 1H), 6.91 (d, *J* = 4.4 Hz, 1H), 6.74 (d, *J* = 7.6 Hz, 1H), 2.63 (s, 3H); ^13^C NMR (100 MHz, CD_3_COCD_3_) *δ* 156.7 (C), 154.3 (C), 148.7 (C), 148.3 (C), 148.2 (C), 128.3 (CH), 124.6 (C), 121.0 (CH), 116.3 (CH), 111.9 (CH), 16.4 (CH_3_); HRMS (ESI) *m*/*z* calcd for C_13_H_10_N_2_O_2_S [M]^+^: 258.0457, found: 258.0461.
*4-(4-Hydroxyphenoxy)-6-methylbenzo[c][1,2,5]thiadiazole-5-carbaldehyde* (**4m**). Yellow solid (19 mg, 22%); M.p. 175–177 °C; ^1^H NMR (400 MHz, CD_3_COCD_3_); *δ* 10.46 (s, 1H), 8.51 (s, 1H), 8.07 (s, 1H), 6.79–6.74 (m, 4H), 2.68 (s, 3H); ^13^C NMR (100 MHz, CD_3_COCD_3_) *δ* 193.5 (CH), 155.4 (C), 153.7 (C), 152.4 (C), 152.3 (C), 145.5 (C), 138.7 (C), 130.6 (C), 126.0 (CH), 117.6 (CH), 116.9 (CH), 12.5 (CH_3_); HRMS (ESI) *m*/*z* calcd for C_14_H_11_N_2_O_3_S [M + H]^+^: 287.0485, found: 287.0486.
*4-((6-Bromo-5-methylbenzo[c][1,2,5]thiadiazol-4-yl)oxy)phenol* (**4n**). Yellow solid (41 mg, 41%); M.P. 180–181 °C; ^1^H NMR (400 MHz, CD_3_COCD_3_); *δ* 8.22 (s, 1H), 6.75 (s, 4H), 4.62(s, 1H), 2.50 (s, 3H); ^13^C NMR (100 MHz, CD_3_COCD_3_) *δ* 156.7 (C), 153.7 (C), 152.2 (C), 149.9 (C), 144.6 (C), 131.2 (C), 129.8 (C), 121.7 (CH), 117.6 (CH), 116.8 (CH), 16.9 (CH_3_); HRMS (ESI) *m*/*z* calcd for C_13_H_10_BrN_2_O_2_S [M + H]^+^: 336.9641, found: 336.9639.
*4-((5-Bromo-6-methylbenzo[c][1,2,5]thiadiazol-4-yl)oxy)phenol* (**4n′**). Colorless solid (34 mg, 34%); M.P. 173–174 °C; ^1^H NMR (400 MHz, CD_3_COCD_3_); *δ* 7.77 (s, 1H), 6.83 (d, *J* = 8.8 Hz, 2H), 6.75 (d, *J* = 8.8 Hz, 2H), 4.63 (s, 1H), 2.67 (d, *J* = 0.8 Hz, 3H); ^13^C NMR (100 MHz, CD_3_COCD_3_) *δ* 156.2 (C), 153.9 (C), 151.6 (C), 149.2 (C), 144.9 (C), 141.5 (C), 121.8 (C), 118.0 (CH), 117.9 (CH), 116.7 (CH), 24.3 (CH_3_);HRMS (ESI) *m*/*z* calcd for C_13_H_10_BrN_2_O_2_S [M + H]^+^: 336.9641, found: 336.9639.

#### 3.1.3. General Process III: Synthesis of Diaryl Ethers (**4o**–**4u**)

To a quartz tube containing a stirring bar, aromatic **1** (0.9 mmol), quinone **2** (0.3 mmol), copper trifluoromethanesulfonate (22 mg, 0.06 mmol), dichloromethane (1.5 mL), and acetonitrile (1.5 mL) were added. The reaction tube was degassed with argon and stirred for 48 h at −40 °C under blue LED (λ = 450 nm, 5 W) irradiation. The reaction system was raised to room temperature and transferred into a single-necked flask. The reaction mixture was concentrated in vacuo and separated by column chromatography (PE/EtOAc = 10:1) to obtain **4**.

*4-Phenoxyphenol* (**4o**) [50]. Colorless solid (54 mg, 95%); M.P. 83–84 °C; ^1^H NMR (400 MHz, CDCl_3_) *δ* 7.33–7.29 (m, 2H), 7.05 (t, *J* = 7.4 Hz, 1H), 6.96–6.93 (m, 4H), 6.84–6.80 (m, 2H), 4.85 (s, 1H); ^13^C NMR (100 MHz, CDCl_3_) *δ* 158.4 (C), 151.6 (C), 150.2 (C), 129.6 (CH), 122.5 (CH), 121.0 (CH), 117.6 (CH), 116.3 (CH); HRMS (ESI) *m*/*z* calcd for C_12_H_11_O_2_ [M + H]^+^: 187.0754, found: 187.0751.
*3-(4-Hydroxyphenoxy)-2,4,6-trimethylbenzoate* (**4p**). Colorless solid (55 mg, 64%); M.P. 153–154 °C; ^1^H NMR (400 MHz, CDCl_3_) *δ* 6.93 (s, 1H), 6.69 (d, *J* = 8.8 Hz, 2H), 6.59 (d, *J* = 8.8 Hz, 2H), 5,09 (s, 1H), 3.91 (s, 3H), 2.28 (s, 3H), 2.075 (s, 3H), 2.071 (s, 3H); ^13^C NMR (100 MHz, CDCl_3_) *δ* 170.4 (C), 151.6 (C), 150.0 (C), 149.3 (C), 133.1 (C), 131.4 (C), 130.6 (CH), 128.8 (C), 116.1 (CH), 115.3 (CH), 52.1 (CH_3_), 19.3(CH_3_), 16.4 (CH_3_), 13.5 (CH_3_); HRMS (ESI) *m*/*z* calcd for C_17_H_19_O_4_ [M + H]^+^: 287.1278, found: 287.1277.
*4-(Naphthalen-1-yloxy)phenol* (**4q**). Colorless liquid (61 mg, 87%); ^1^H NMR (400 MHz, CDCl_3_) *δ* 8.31 (d, *J* = 8.8 Hz, 1H), 7.88 (d, *J* = 8.8 Hz, 1H), 7.58–7.52 (m, *3*H), 7.35 (t, *J* = 8.0 Hz, 1H), 7.00 (d, *J* = 8.8 Hz, 2H), 6.85–6.81 (m, 3H), 4.32 (s, 1H); ^13^C NMR (100 MHz, CDCl_3_) *δ* 154.3 (C), 151.6 (C), 150.7 (C), 134.8 (C), 127.6 (CH), 126.6 (CH), 126.3 (C), 125.8 (CH), 125.7 (CH), 122.4 (CH), 122.0 (CH), 120.7 (CH), 116.4 (CH), 111.3 (CH); HRMS (ESI) *m*/*z* calcd for C_16_H_13_O_2_ [M + H]^+^: 237.0910, found: 237.0908.
*4-(Benzo[c][1,2,5]thiadiazol-4-yloxy)-2-methylphenol* (**4r**). Light-yellow solid (28 mg, 34%); M.P. 142–143 °C; ^1^H NMR (400 MHz, CDCl_3_), *δ* 7.62 (dd, *J* = 8.8 Hz, 0.8 Hz, 1H), 7.41–7.37 (m, 1H), 6.87 (d, *J* = 8.4 Hz 1H), 6.72 (d, *J* = 8.8 Hz, 1H), 6.43 (dd, *J* = 7.6 Hz, 0.8 Hz, 1H), 5.25 (s, 1H), 2.23 (s, 3H), 2.12 (s, 3H); ^13^C NMR (100 MHz, CDCl_3_) *δ* 156.5 (C), 151.1 (C), 150.9 (C), 147.9 (C), 146.0 (C), 130.7 (C), 130.1 (CH), 124.6 (C), 119.2 (CH), 114.2 (CH), 113.3 (CH), 109.0 (CH), 12.8 (CH_3_), 12.2 (CH_3_). HRMS (ESI) *m*/*z* calcd for C_14_H_12_N_2_O_2_S [M + H]^+^: 273.0692, found: 273.0692.
*2,3-Dimethyl-4-phenoxyphenol* (**4s**). Colorless solid (28 mg, 44%); M.P. 79–80 °C; ^1^H NMR (400 MHz, CDCl_3_) *δ* 7.28–7.24 (m, 1H), 6.98 (t, *J* = 7.6 Hz, 1H), 6.82 (d, *J* = 8.0 Hz, 2H), 6.74 (d, *J* = 8.4 Hz, 1H), 6.63 (d, *J* = 8.4 Hz, 1H), 4.63 (s, 1H), 2.21 (s, 3H), 2.12 (s, 3H); ^13^C NMR (100 MHz, CDCl_3_) *δ* 159.0 (C), 150.2 (C), 147.2 (C), 130.8 (C), 129.5 (CH), 124.2 (C), 121.5 (CH), 119.0 (CH), 116.0 (CH), 113.0 (CH), 12.8 (CH_3_), 12.1 (CH_3_); HRMS (ESI) *m*/*z* calcd for C_14_H_15_O_2_ [M + H]^+^: 215.1067, found: 215.1064.
*4-(Benzo[c][1,2,5]thiadiazol-4-yloxy)-2-chlorophenol* (**4t**). Light-yellow solid (20 mg, 24%); M.P. 140–142 °C; ^1^H NMR (400 MHz, CDCl_3_), *δ* 7.71 (d, *J* = 8.8 Hz, 1H), 7.49–7.45 (m, 1H), 7.20 (d, *J* = 2.4 Hz, 1H), 7.09–7.03 (m, 2H), 6.78 (d, *J* = 7.2 Hz, 1H), 5.51 (s, 1H); ^13^C NMR (100 MHz, CDCl_3_) *δ* 150.0 (C) *δ* 148.7 (C), *δ* 148.5 (C), *δ* 129.8 (CH), *δ* 121.0 (CH), *δ* 120.7 (CH), *δ* 120.2 (C), *δ* 117.0 (CH), *δ* 115.8 (CH), *δ* 111.5 (CH); HRMS (ESI) *m*/*z* calcd for C_12_H_8_ClN_2_O_2_S [M + H]^+^: 278.9990, found: 278.9986.
*2-Chloro-4-phenoxyphenol* (**4u**) [51]. Red liquid (59 mg, 89%); ^1^H NMR (400 MHz, CDCl_3_) *δ* 7.36–7.32 (m, 2H), 7.11 (t, *J* = 7.2 Hz, 1H), 7.055 (d, *J* = 2.4 Hz, 1H), 7.03–6.97 (m, 3H), 6.915 (dd, *J* = 8.8 Hz, 2.8 Hz, 1H), 5.48 (s, 1H); ^13^C NMR (100 MHz, CDCl_3_) *δ* 157.6 (C), 150.2 (C), 147.6 (C), 129.7 (CH), 123.1 (CH), 120.0 (CH), 119.97 (CH), 119.7 (C), 117.9 (CH), 116.7 (CH); HRMS (ESI) *m*/*z* calcd for C_12_H_10_ClO_2_ [M + H]^+^: 221.0364, found: 221.0360.

#### 3.1.4. Synthesis of **5**

*4-((4,6-Dimethylbenzo[c][1,2,5]thiadiazol-5-yl)oxy)phenol* (**5**). Compound **3a** (82 mg, 0.30 mmol) was dissolved in a mixed solvent of acetonitrile and dichloromethane and then copper triflate (22 mg, 0.06 mmol) was added in an argon atmosphere. After TLC monitoring, the raw material disappeared. Then, the solvent was directly removed for column chromatography (PE/EtOAc = 10:1). Green solid (8 mg, 10%); M.P. 142–144 °C; ^1^H NMR (400 MHz, CDCl_3_) *δ* 7.71 (s, 1H), 6.75 (d, *J* = 8.8 Hz, 2H),6.69 (d, *J* = 8.8 Hz, 2H), 4.71 (s, 1H), 2.53 (s, 3H), 2.30 (s, 3H); ^13^C NMR (100 MHz, CDCl_3_) *δ* 155.4, 152.7, 152.4, 151.7, 150.4, 137.2, 122.4, 118.6, 116.4, 115.6, 18.2, 11.9; HRMS (ESI) *m*/*z* calcd for C_14_H_13_N_2_O_2_S [M + H]^+^: 273.0692, found: 273.0692.

### 3.2. X-ray Crystallographic Analysis

The structure of **3a**, **4b**, **4k**, **4n**, **4t**, and **5** was confirmed based on single-crystal X-ray analysis.

CCDC No. 2121130 (**3a**), 2091206 (**4b**), 2121129 (**4k**), 2121131 (**4n**), 2091197 (**4t**), and 2091205 (**5**) contain the supplementary crystallographic data for this paper. The crystal data can be obtained free of charge from the Cambridge Crystallographic Data Centre at www.ccdc.cam.ac.uk/datarequest/cif (accessed on 9 September 2022).

## 4. Conclusions

In summary, a PB reaction between aromatics and quinones under visible light irradiation was developed. The PB reactions between aromatics and quinones were accomplished via activation of the benzene ring double bond using the combination of thiadiazole, oxadiazole, or selenadiazole under blue LED irradiation, resulting in the synthesis of oxetanes with an obvious substituent effect. The addition of Cu(OTf)_2_ opened the generated oxetane rings in situ to transform into diaryl ethers, which expanded the range of substrate adaptation.

## Figures and Tables

**Table 1:** The Paternò–Büchi reaction of aromatic with quinones ^a^.

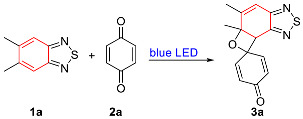					
Entry	1a:2a (Equiv.)	Photosensitizers (5 mmol%)	Solvent	Temp. (°C)	Yield (%) ^b^
1	1:1	-	CH_3_CN	40	25
2	1:3	-	CH_3_CN	40	33
3	2:1	-	CH_3_CN	40	32
4	3:1	-	CH_3_CN	40	43
5 ^[c]^	3:1	-	CH_3_CN	40	7
6	3:1	Ir(ppy)_3_	CH_3_CN	40	np ^[d]^
7	3:1	Ru(bpy)_3_Cl_2_·6H_2_O	CH_3_CN	40	32
8	3:1	Thioxanthen-9-one	CH_3_CN	40	40
9	3:1	-	CH_3_CN	–40	48
10	3:1	-	Et_2_O	–78	np
11	3:1	-	THF	–78	np
12	3:1	-	DCM	–78	74
13 ^[e]^	3:1	-	DCM	–78	np

^a^ Reaction conditions: **1a** and **2a** (0.30 mmol) in the solvent (3 mL) under blue LED irradiation for 48 h. ^b^ Isolated yields. ^[c]^ Light source: 254 nm. ^[d]^ np = no product. ^[e]^ No blue LED irradiation.

**Table 2:** Optimization of the reaction conditions for the synthesis of diaryl ethers ^a^.

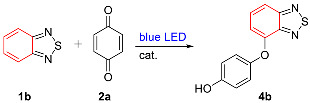					
Entry	1b:2a (Equiv.)	Catalyst (% ^b^)	Solvent	Temp. (°C)	Yield (%) ^c^
1	3:1	Cu(OTf)_2_ (10)	CH_3_CN	40	50
2 ^d^	3:1	Cu(OTf)_2_ (10)	CH_3_CN	40	np
3 ^e^	3:1	Cu(OTf)_2_ (10)	CH_3_CN	40	23
4 ^f^	3:1	Cu(OTf)_2_ (10)	CH_3_CN	40	46
5	3:1	Fe(OTf)_3_ (10)	CH_3_CN	40	45
6	3:1	Zn(OTf)_2_ (10)	CH_3_CN	40	43
7	3:1	Yb(OTf)_3_ (10)	CH_3_CN	40	16
8	3:1	Nd(OTf)_3_ (10)	CH_3_CN	40	15
9	3:1	Sc(OTf)_3_ (10)	CH_3_CN	40	10
10	3:1	Pd(OAc)_2_ (10)	CH_3_CN	40	np
11	3:1	AgOAc (10)	CH_3_CN	40	np
12	3:1	BF_3_·Et_2_O (10)	CH_3_CN	40	np
13	3:1	Cu(OTf)_2_ (10)	CH_3_CN	25	53
14	3:1	Cu(OTf)_2_ (10)	CH_3_CN	10	44
15	1:1	Cu(OTf)_2_ (10)	CH_3_CN	25	28
16	1.5:1	Cu(OTf)_2_ (10)	CH_3_CN	25	37
17	2:1	Cu(OTf)_2_ (10)	CH_3_CN	25	39
18	1:1.5	Cu(OTf)_2_ (10)	CH_3_CN	25	39
19	3:1	Cu(OTf)_2_ (20)	CH_3_CN	25	65
20	3:1	Cu(OTf)_2_ (20)	CH_3_OH	25	np
21	3:1	Cu(OTf)_2_ (20)	acetone	25	np
22	3:1	Cu(OTf)_2_ (20)	Et_2_O	25	np
23	3:1	Cu(OTf)_2_ (20)	THF	25	np
24	3:1	Cu(OTf)_2_ (20)	DCM	25	45
25	3:1	Cu(OTf)_2_ (20)	CH_3_CN/ DCM	25	67
26 ^g^	3:1	Cu(OTf)_2_ (20)	CH_3_CN/ DCM	25	np

^a^ Reaction conditions: **1b** and **2a** (0.30 mmol) in the solvent (3 mL) under blue LED irradiation for 48 h. ^b^ The mole ratio of **2a**. ^c^ Isolated yields. ^d^ Ir(bpy)_3_ (5% mole of **2a**) was added. ^e^ Ru(bpy)_3_Cl_2_·6H_2_O (5% mole of **2a**) was added. ^f^ Thioxanthen-9-one (5% mole of **2a**) was added. ^g^ No blue LED irradiation.

## Data Availability

Data are contained within the article and Supplementary Materials.

## Supplementary Materials

The following supporting information can be downloaded at: https://www.mdpi.com/article/10.3390/molecules29071513/s1, Figure S1: UV-vis absorption spectra of **2a**; Figure S2: The fluorescence spectra of **2a**; Figure S3: Cyclic voltammetry (CV) curve of **1a**; Table S1: Optimization of the reaction conditions for the synthesis of diaryl ethers; Crytallographic data; Copies of NMR spectra; DFT calculations data. References [13,47,52,53,54] are cited in the Supplementary Materials.

## References

[1] Shimada N., Hasegawa S., Harada T., Tomisawa T., Fujii A., Takita T. (1986). Oxetanocin, a novel nucleoside from bacteria. J. Antibiot..

[2] Bhagwat S.S., Hamann P.R., Still W.C. (1985). Synthesis of thromboxane A2. J. Am. Chem. Soc..

[3] Omura S., Murata M., Imamura N., Iwai Y., Tanaka H., Furusaki A., Matsumoto H. (1984). Oxetin, a new antimetabolite from an actinomycete fermentation, isolation, structure and biological activity. J. Antibiot..

[4] Loh J., Carlson R.W., York W.S., Stacey G. (2002). Bradyoxetin, a unique chemical signal involved in symbiotic gene regulation. Proc. Natl. Acad. Sci. USA.

[5] Wang J.-P., Shu Y., Liu S.-X., Hu J.-T., Sun C.-T., Zhou H., Gan D., Cai X.-Y., Pu W., Cai L. (2019). Expanstines A–D: Four unusual isoprenoid epoxycyclohexenones generated by Penicillium expansum YJ-15 fermentation and photopromotion. Org. Chem. Front..

[6] Becker M.R., Wearing E.R., Schindler C.S. (2020). Synthesis of azetidines via visible-light-mediated intermolecular [2+2] photocycloadditions. Nat. Chem..

[7] Richardson A.D., Becker M.R., Schindler C.S. (2020). Synthesis of azetidines by aza Paternò-Büchi reactions. Chem. Sci..

[8] Kandappa S.K., Valloli L.K., Ahuja S., Parthiban J., Sivaguru J. (2021). Taming the excited state reactivity of imines—From non-radiative decay to aza Paternò-Büchi reaction. Chem. Soc. Rev..

[9] Li X., Grosskopf J., Jandl C., Bach T. (2021). Enantioselective, visible light mediated aza Paternò-Büchi reactions of quinoxalinones. Angew. Chem. Int. Ed..

[10] D’Auria M. (2019). The Paternò-Büchi reaction–a comprehensive review. Photochem. Photobiol. Sci..

[11] Flores D.M., Schmidt V.A. (2019). Intermolecular 2 + 2 Carbonyl-Olefin Photocycloadditions Enabled by Cu(I)-Norbornene MLCT. J. Am. Chem. Soc..

[12] Franceschi P., Mateos J., Vega-Penaloza A., Dell’Amico L. (2020). Microfluidic visible-light Paternò-Büchi reaction of oxindole enol ethers. Eur. J. Org. Chem..

[13] Fréneau M., Hoffmann N. (2017). The Paternò-Büchi reaction–Mechanisms and application to organic synthesis. J. Photochem. Photobiol. C Photochem. Rev..

[14] Buchi G., Ihman C.G., Lipinsky E.S. (1954). Light-catalyzed organic reactions. I. The reaction of carbonyl compounds with 2-methyl-2-butene in the presence of ultraviolet light. J. Am. Chem. Soc..

[15] Paterno E., Chieffi G. (1909). Sintesi in chimica organica per mezzo della luce. Composti degli idrocarbun non saturi con aldeidi e chetoni. Gazz. Chim. Ital..

[16] Friedrich L.E., Bower J.D. (1973). Detection of an oxetene intermediate in the photoreaction of benzaldehyde with 2-butyne. J. Am. Chem. Soc..

[17] D’Auria M., Racioppi R., Romaniello G. (2000). The Paternò-Büchi reaction of 2-furylmethanols. Eur. J. Org. Chem..

[18] D’Auria M., Racioppi R. (2000). Paterno-Büchi reaction on 5-methyl-2-furylmethanol derivatives. Arkivoc.

[19] Vargas F., Rivas C., Navarro M., Alvarado Y. (1996). Synthesis of an elusive oxetane by photoaddition of benzophenone to thiophene in the presence of a Lewis acid. J. Photochem. Photobiol. A Chem..

[20] Capozzo M., D’Auria M., Emanuele L., Racioppi R. (2007). Synthesis and photochemical reactivity towards the Paternò-Büchi reaction of benzo[b]furan derivatives: Their use in the preparation of 3-benzofurylmethanol derivatives. J. Photochem. Photobiol. A Chem..

[21] Rivas C., Velez M., Crescente O. (1970). Synthesis of an oxetan by photoaddition of benzophenone to a thiophen derivative. Chem. Commun..

[22] Jones G., Gilow H.M., Low J. (1979). Regioselective photoaddition of pyrroles and aliphatic carbonyl compounds. A new synthesis of 3(4)-substituted pyrroles. J. Org. Chem..

[23] Rivas C., Bolivar R.A. (1976). Synthesis of oxetanes by photoaddition of carbonyl compounds to pyrrole derivatives. J. Heterocycl. Chem..

[24] Matsuura T., Banba A., Ogura K. (1971). Photoinduced reactions–XLV: Photoaddition of ketones to methylimidazoles. Tetrahedron.

[25] Nakano T., Rodriguez W., De Roche S.Z., Larrauri J.M., Rivas C., Perez C. (1980). Photoaddition of ketones to imidazoles, thiazoles, isothiazoles and isoxazoles. Synthesis of their oxetanes. J. Heterocycl. Chem..

[26] Griesbeck A.G., Fiege M., Lex J. (2000). Oxazole–Carbonyl photocycloadditions: Selectivity pattern and synthetic route to erythro α-amino, β-hydroxy ketones. Chem. Commun..

[27] Mateos J., Vega-Penaloza A., Franceschi P., Rigodanza F., Andreetta P., Companyo X., Pelosi G., Bonchio M., Dell’Amico L. (2020). A visible-light Paternò-Büchi dearomatisation process towards the construction of oxeto-indolinic polycycles. Chem. Sci..

[28] Wang T., Stein P.M., Shi H., Hu C., Rudolph M., Hashmi A.S.K. (2021). Hydroxylamine-mediated C–C amination via an aza-hock rearrangement. Nat. Commun..

[29] Wang T., Hoffmann M., Dreuw A., Hasagić E., Hu C., Stein P.M., Witzel S., Shi H., Yang Y., Rudolph M. (2021). A Metal-Free Direct Arene C−H Amination. Adv. Synth. Catal..

[30] Becker M.R., Richardson A.D., Schindler C.S. (2019). Functionalized azetidines via visible light-enabled aza Paternò-Büchi reactions. Nat. Commun..

[31] Blum T.R., Miller Z.D., Bates D.M., Guzei I.A., Yoon T.P. (2016). Enantioselective photochemistry through Lewis acid-catalyzed triplet energy transfer. Science.

[32] Strieth-Kalthoff F., James M.J., Teders M., Pitzer L., Glorius F. (2018). Energy transfer catalysis mediated by visible light: Principles, applications, directions. Chem. Soc. Rev..

[33] Zhou Z., LaPointe A.M., Shaffer T.D., Coates G.W. (2023). Nature-inspired methylated polyhydroxybutyrates from C1 and C4 feedstocks. Nat. Chem..

[34] Getzler Y.D.Y.L., Kundnani V., Lobkovsky E.B., Coates G.W. (2004). Catalytic Carbonylation of β-Lactones to Succinic Anhydrides. J. Am. Chem. Soc..

[35] Theil F. (1999). Synthesis of diaryl ethers: A long-standing problem has been solved. Angew. Chem. Int. Ed..

[36] Rao A.V.R., Gurjar M.K., Reddy K.L., Rao A.S. (1995). Studies directed toward the synthesis of vancomycin and related cyclic peptides. Chem. Rev..

[37] Zhu J. (1997). SNAr bases macrocyclization via biaryl ether formation: Application in natural product synthesis. Synlett.

[38] Boger D.L., Borzilleri R.M., Nukui S., Beresis R.T. (1997). Synthesis of the vancomycin CD and DE ring systems. J. Org. Chem..

[39] Mulla S.A.R., Inamdar S.M., Pathan M.Y., Chavan S.S. (2012). Ligand free, highly efficient synthesis of diaryl ether over copper fluorapatite as heterogeneous reusable catalyst. Tetrahedron Lett..

[40] Zhang Y., Ni G., Li C., Xu S., Zhang Z., Xie X. (2015). The coupling reactions of aryl halides and phenols catalyzed by palladium and MOP-type ligands. Tetrahedron.

[41] Cristau H.-J., Cellier P.P., Hamada S., Spindler J.-F., Taillefer M. (2004). A general and mild ullmann-type synthesis of fiaryl ethers. Org. Lett..

[42] Munoz A., Leo P., Orcajo G., Martínez F., Calleja G. (2019). URJC-1-MOF as new heterogeneous recyclable catalyst for C-Heteroatom coupling reactions. ChemCatChem.

[43] Cermak J.K., Cirkva V. (2014). Copper-mediated synthesis of mono- and dichlorinated diaryl ethers. Tetrahedron Lett..

[44] Chan D.M.T., Monaco K.L., Wang R.-P., Winters M.P. (1998). New N- and O-arylations with phenylboronic acids and cupric acetate. Tetrahedron Lett..

[45] Evans D.A., Katz J.L., West T.R. (1998). Synthesis of diaryl ethers through the copper-promoted arylation of phenols with arylboronic acids. An expedient synthesis of thyroxine. Tetrahedron Lett..

[46] Sang R., Korkis S.E., Su W., Ye F., Engl P.S., Berger F., Ritter T. (2019). Site-selective C−H oxygenation via aryl sulfonium salts. Angew. Chem. Int. Ed..

[47] Sharma A., Dixit V., Kumar S., Jain N. (2021). Visible Light-Mediated In Situ Generation of δ,δ-Disubstituted p-Quinone Methides: Construction of a Sterically Congested Quaternary Stereocenter. Org. Lett..

[48] Franceschi P., Cuadros S., Goti G., Dell’Amico L. (2023). Mechanisms and Synthetic Strategies in Visible Light-Driven [2+2]-Heterocycloadditions. Angew. Chem. Int. Ed..

[49] Ciufolini M.A., Rivera-Fortin M.A., Zuzukin V., Whitmire K.H. (1994). Origin of Regioselectivity in Paterno-Buechi Reactions of Benzoquinones with Alkylidenecycloalkanes. J. Am. Chem. Soc..

[50] Janssen D.E., VanAllan J., Wilson C.V. (1955). The synthesis of 4-phenoxycatechol and 2-phenoxyhydroquinone. J. Org. Chem..

[51] Koyama H., Miller D.J., Boueres J.K., Desai R.C., Jones A.B., Berger J.P., MacNaul K.L., Kelly L.J., Doebber T.W., Wu M.S. (2004). (2R)-2-Ethylchromane-2-carboxylic Acids: Discovery of Novel PPARα/γ Dual Agonists as Antihyperglycemic and Hypolipidemic Agents. J. Med. Chem..

[52] Sagadevan A., Ragupathi A., Hwang K.C. (2015). Photoinduced Copper-Catalyzed Regioselective Synthesis of Indoles: Three-Component Coupling of Arylamines, Terminal Alkynes, and Quinones. Angew. Chem. Int. Ed..

[53] Schnapp K.A., Wilson R.M., Ho D.M., Caldwell R.A., Creed D. (1990). Benzoquinone-olefin exciplexes: The observation and chemistry of the p-benzoquinone-tetraphenylallene exciplex. J. Am. Chem. Soc..

[54] Yang J., Duan J., Wang G., Zhou H., Ma B., Wu C., Xiao J. (2020). Visible-Light-Promoted Site-Selective N(1)-Alkylation of Benzotriazoles with alpha-Diazoacetates. Org. Lett..

