# Supplementary material for: A Paternò–Büchi Reaction of Aromatics with Quinones under Visible Light Irradiation
Source: Molecules. 2024 Mar 28;29(7):1513. doi: 10.3390/molecules29071513 (PMC11013315; doi:10.3390/molecules29071513)
Supplement: Supplementary file 1 [file molecules-29-01513-s001.zip › molecules-2906194-supplementary.pdf]

Supplementary Material

A Paternò–Büchi Reaction of Aromatics with Quinones under Visible Light Irradiation

Wen-Wen Li †, Jia-Lin Zhao †, Ze-Yu Wang, Pei-Ting Li, Zi-Fa Shi *, Xiao-Ping Cao and Qiang Liu *

State Key Laboratory of Applied Organic Chemistryand College of Chemistry & Chemical Engineering, Lanzhou University, Lanzhou 730000, China; liww21@stu.xjtu.edu.cn (W.-W.L.); jlzhao20@lzu.edu.cn (J.-L.Z.); zywang19@lzu.edu.cn (Z.-Y.W.); lipt19@lzu.edu.cn (P.-T.L.); caoxp@lzu.edu.cn (X.-P.C.)

* Correspondence: shizf@lzu.edu.cn (Z.-F.S.); liuqiang@lzu.edu.cn (Q.L.); Tel.: +86-931-8912500 (Z.-F.S.)

† These authors contributed equally to this work.

Table of Contents

1. General information S2
2. Optimization of the reaction conditions of 1o and 2a S2
3. Crytallographic data S3
4. Copies of ^{1}H, ^{13}C, ^{19}F, and DEPT 135 NMR spectra S9
5. Mechanistic investigation S50
6. DFT calculations data S53

1. General information

All commercial reagents are directly purchased and used. The reactions that require anhydrous and anaerobic operations are protected by argon atmosphere. The solvents used in the experiment are used after drying and re-evaporating in strict accordance with the "Laboratory Chemical Purification Manual". The reaction process is monitored by thin layer chromatography (TLC). The product was separated and purified by silica gel column chromatography. The eluent used petroleum ether (PE) with a boiling range of 60–90 °C for thin layer chromatography silica gel (GF254) and column chromatography silica gel (200–300 mesh). Bruker AM 400 MHz, 600 MHz nuclear magnetic resonance instrument was used for ^{1}H, ^{13}C determination, and TMS was used as internal standard. The melting point was measured by a Reichert Microscope melting point instrument, and the high resolution mass spectrometer was measured by Bruker Daltonics APEX II 47e FT-ICR high resolution mass spectrometer. Single crystal X-ray diffraction measurements were made on a XtaLAB Synerogy DW instrument. The light source of visible light irradiation reactions is a 5 W blue LED light (450 nm). The reaction apparatus for low temperature blue light irradiation is assembled on a low temperature reactor as shown below.

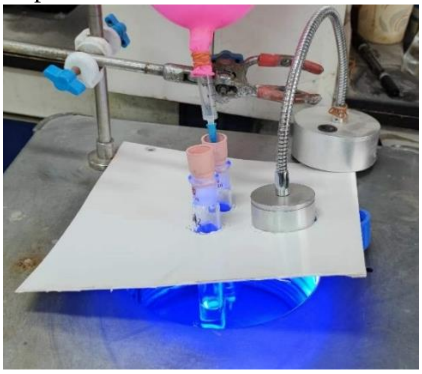

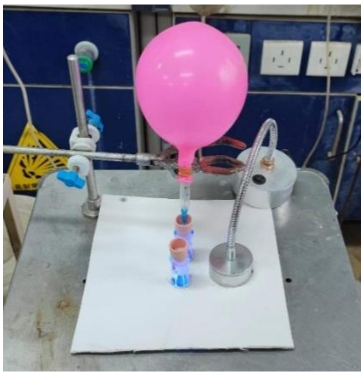

2. Optimization of the reaction conditions of 1o and 2a

Table S1 Optimization of the reaction conditions for the synthesis of diaryl ethers

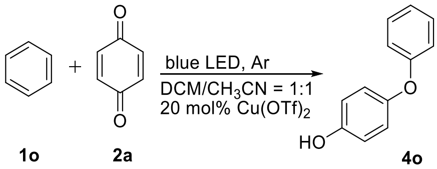

Entry[a]	T (°C)	**1o** : **2a** (equiv.)	Yields (%)[b]
1	25	3:1	46
2	10	3:1	54
3	0	3:1	66
4	−20	3:1	84
5	−40	3:1	95
6	−78	3:1	91
7	−40	1:1	59
8	−40	2:1	73

[a]Reaction conditions: **1o** and **2a** (0.30 mmol) in DCM/CH_3CN (1:1, 3 mL) under blue LED (λ = 450 nm) for 48 h. [b]isolated yields.

3. Crytallographic data

Solid state molecular structure of 3a

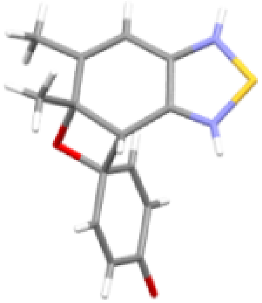

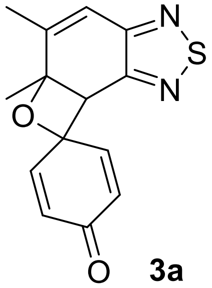

3a

Crystal data	
CCDC number	2121130
Chemical formula	$C_{14}H_{12}N_2O_2S$
M_r	274.33
Crystal system, space group	Orthorhombic, *Pbcn*
Temperature (K)	293
a, *b*, *c* (Å)	23.1593 (11), 8.0634 (4), 14.5089 (8)
V (Å^3)	2709.4 (2)
Z	8
Radiation type	Cu *K*α
μ (mm^{-1})	2.12
Crystal size (mm)	× ×
Data collection	
Diffractometer	XtaLAB Synergy, Dualflex, HyPix
Absorption correction	Multi-scan<br>*CrysAlis PRO* 1.171.39.34b (Rigaku Oxford Diffraction, 2017) Empirical absorption correction using spherical harmonics, implemented in SCALE3 ABSPACK scaling algorithm.
T_{min}, T_{max}	0.777, 1.000
No. of measured, independent and observed [$I > 2\sigma(I)$] reflections	7605, 2618, 2205
R_{int}	0.026
$(\sin \theta/\lambda)_{max}$ (Å^{-1})	0.624
Refinement	
$R[F^2 > 2\sigma(F^2)]$, $wR(F^2)$, S	0.061, 0.187, 1.06
No. of reflections	2618
No. of parameters	174
H-atom treatment	H-atom parameters constrained
$\Delta\rho_{max}$, $\Delta\rho_{min}$ (e Å^{-3})	0.30, −0.43

Solid state molecular structure of 4b

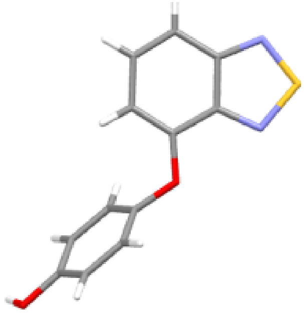

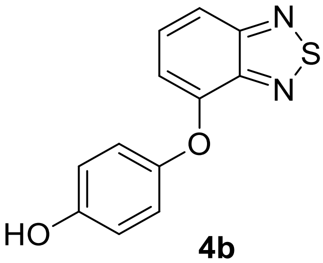

4b

Crystal data	
CCDC number	2091206
Chemical formula	$C_{12}H_8N_2O_2S$
M_r	244.26
Crystal system, space group	Monoclinic, $P2_1/c$
Temperature (K)	292
a, *b*, *c* (Å)	7.9998 (2), 10.0316 (3), 13.7380 (4)
β (°)	96.915 (3)
V (Å^3)	1094.47 (6)
Z	4
Radiation type	Cu *K*α
μ (mm^{-1})	2.56
Crystal size (mm)	0.13 × 0.11 × 0.07
Data collection	
Diffractometer	SuperNova, Dual, Cu at zero, Eos
Absorption correction	Multi-scan<br>*CrysAlis PRO* 1.171.38.43f (Rigaku Oxford Diffraction, 2015) Empirical absorption correction using spherical harmonics, implemented in SCALE3 ABSPACK scaling algorithm.
T_{min}, T_{max}	0.672, 1.000
No. of measured, independent and observed [$I > 2\sigma(I)$] reflections	3757, 1915, 1797
R_{int}	0.013
$(\sin \theta/\lambda)_{max}$ (Å^{-1})	0.595
Refinement	
$R[F^2 > 2\sigma(F^2)]$, $wR(F^2)$, S	0.036, 0.098, 1.07
No. of reflections	1915
No. of parameters	155
H-atom treatment	H-atom parameters constrained
$\Delta\rho_{max}$, $\Delta\rho_{min}$ (e Å^{-3})	0.22, −0.45

Solid state molecular structure of 4k

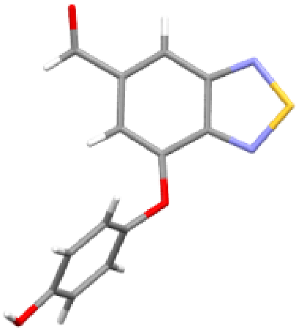

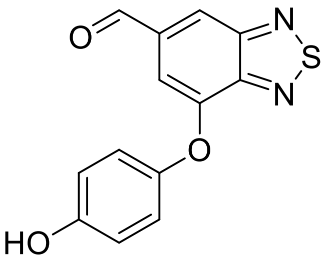

4k

Crystal data	
CCDC number	2121129
Chemical formula	$C_{13}H_8N_2O_3S$
M_r	272.27
Crystal system, space group	Monoclinic, $P2_1/c$
Temperature (K)	299
a, *b*, *c* (Å)	7.9222 (1), 11.0501 (1), 13.4314 (2)
β (°)	99.517 (1)
V (Å^3)	1159.62 (3)
Z	4
Radiation type	Cu *K*α
μ (mm^{-1})	2.55
Crystal size (mm)	0.18 × 0.15 × 0.12
Data collection	
Diffractometer	ROD, Synergy Custom system, HyPix
Absorption correction	Multi-scan<br>*CrysAlis PRO* 1.171.40.84a (Rigaku Oxford Diffraction, 2020) Empirical absorption correction using spherical harmonics, implemented in SCALE3 ABSPACK scaling algorithm.
T_{min}, T_{max}	0.574, 1.000
No. of measured, independent and observed [$I > 2\sigma(I)$] reflections	6494, 2221, 2154
R_{int}	0.019
$(\sin \theta/\lambda)_{max}$ (Å^{-1})	0.629
Refinement	
$R[F^2 > 2\sigma(F^2)]$, $wR(F^2)$, S	0.035, 0.093, 1.06
No. of reflections	2221
No. of parameters	173
H-atom treatment	H-atom parameters constrained
$\Delta\rho_{max}$, $\Delta\rho_{min}$ (e Å^{-3})	0.22, −0.30

Solid state molecular structure of 4n

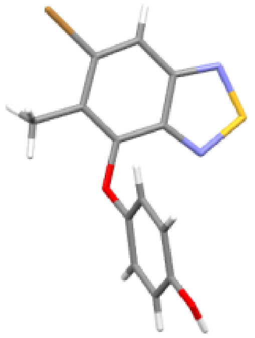

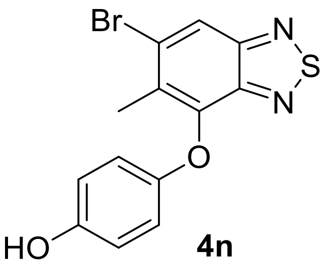

4n

Crystal data	
CCDC number	2121131
Chemical formula	$C_{13}H_9BrN_2O_2S$
M_r	337.18
Crystal system, space group	Triclinic, *P*1
Temperature (K)	150
a, *b*, *c* (Å)	7.2789 (2), 9.2558 (2), 9.8648 (3)
α, β, γ (°)	73.824 (2), 79.464 (2), 88.141 (2)
V (Å^3)	627.42 (3)
Z	2
Radiation type	Cu *K*α
μ (mm^{-1})	6.02
Crystal size (mm)	0.15 × 0.13 × 0.11
Data collection	
Diffractometer	ROD, Synergy Custom system, HyPix
Absorption correction	Multi-scan<br>*CrysAlis PRO* 1.171.40.84a (Rigaku Oxford Diffraction, 2020) Empirical absorption correction using spherical harmonics, implemented in SCALE3 ABSPACK scaling algorithm.
T_{min}, T_{max}	0.371, 1.000
No. of measured, independent and observed [$I > 2\sigma(I)$] reflections	5746, 2445, 2411
R_{int}	0.027
$(\sin \theta/\lambda)_{max}$ (Å^{-1})	0.629
Refinement	
$R[F^2 > 2\sigma(F^2)]$, $wR(F^2)$, S	0.030, 0.079, 1.00
No. of reflections	2445
No. of parameters	174
H-atom treatment	H-atom parameters constrained
$\Delta\rho_{max}$, $\Delta\rho_{min}$ (e Å^{-3})	0.55, −0.73

Solid state molecular structure of 4t

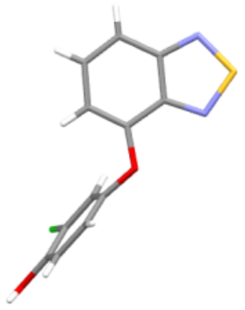

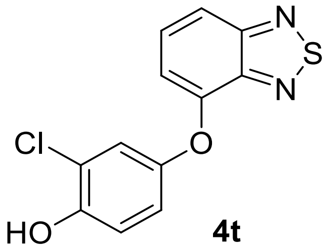

4t

Crystal data	
CCDC number	2091197
Chemical formula	$C_{12}H_7ClN_2O_2S$
M_r	278.71
Crystal system, space group	Monoclinic, $P2_1/c$
Temperature (K)	299
a, *b*, *c* (Å)	7.4320 (2), 20.8735 (4), 7.94233 (19)
β (°)	106.075 (3)
V (Å^3)	1183.93 (5)
Z	4
Radiation type	Cu *K*α
μ (mm^{-1})	4.48
Crystal size (mm)	0.11 × 0.1 × 0.08
Data collection	
Diffractometer	ROD, Synergy Custom system, HyPix
Absorption correction	Multi-scan *CrysAlis PRO* 1.171.40.84a (Rigaku Oxford Diffraction, 2020) Empirical absorption correction using spherical harmonics, implemented in SCALE3 ABSPACK scaling algorithm.
T_{min}, T_{max}	0.187, 1.000
No. of measured, independent and observed [$I > 2\sigma(I)$] reflections	6649, 2313, 1848
R_{int}	0.105
$(\sin \theta/\lambda)_{max}$ (Å^{-1})	0.629
Refinement	
$R[F^2 > 2\sigma(F^2)]$, $wR(F^2)$, S	0.108, 0.279, 1.11
No. of reflections	2313
No. of parameters	164
H-atom treatment	H-atom parameters constrained
$\Delta\rho_{max}$, $\Delta\rho_{min}$ (e Å^{-3})	0.60, −0.75

Solid state molecular structure of 5

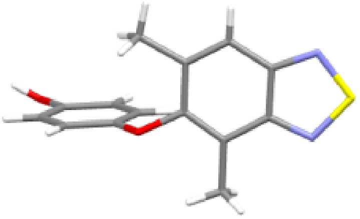 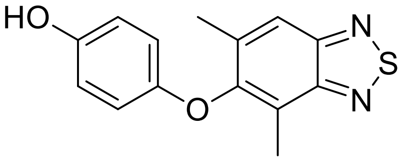

5

Crystal data	
CCDC number	2091205
Chemical formula	$C_{14}H_{12}N_2O_2S$
M_r	272.32
Crystal system, space group	Monoclinic, *C*2/*c*
Temperature (K)	302
a, *b*, *c* (Å)	22.5406 (7), 5.8616 (1), 21.3423 (6)
β (°)	115.025 (3)
V (Å^3)	2555.11 (13)
Z	8
Radiation type	Cu *K*α
μ (mm^{-1})	2.25
Crystal size (mm)	0.07 × 0.04 × 0.01
Data collection	
Diffractometer	XtaLAB Synergy R, DW system, HyPix
Absorption correction	Multi-scan<br>*CrysAlis PRO* 1.171.41.105a (Rigaku Oxford Diffraction, 2021) Empirical absorption correction using spherical harmonics, implemented in SCALE3 ABSPACK scaling algorithm.
T_{min}, T_{max}	0.449, 1.000
No. of measured, independent and observed [$I > 2\sigma(I)$] reflections	7683, 2527, 2281
R_{int}	0.028
$(\sin \theta/\lambda)_{max}$ (Å^{-1})	0.629
Refinement	
$R[F^2 > 2\sigma(F^2)]$, $wR(F^2)$, S	0.043, 0.127, 1.08
No. of reflections	2527
No. of parameters	175
H-atom treatment	H-atom parameters constrained
$\Delta\rho_{max}$, $\Delta\rho_{min}$ (e Å^{-3})	0.26, −0.35

4. Copies of ^{1}H, ^{13}C, ^{19}F, and DEPT 135 NMR spectra

^{1}H NMR ($CDCl_3$, 400 MHz), ^{13}C NMR ($CDCl_3$, 100 MHz), and DEPT 135 spectra of **3a**

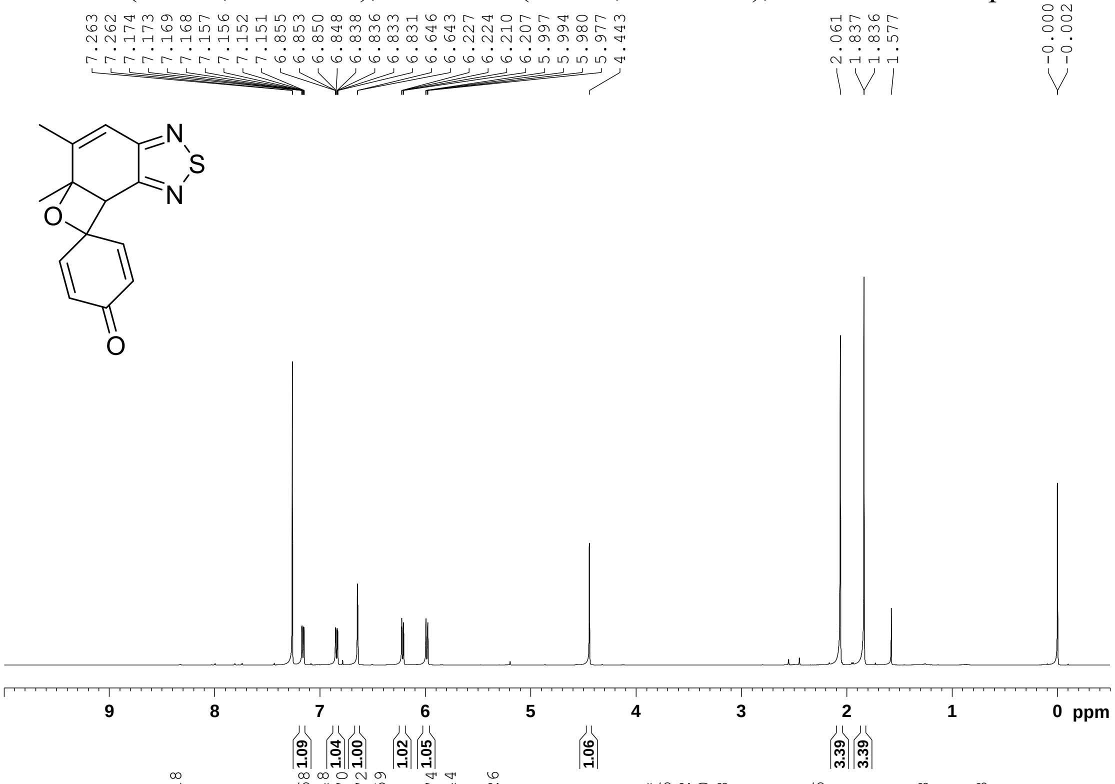

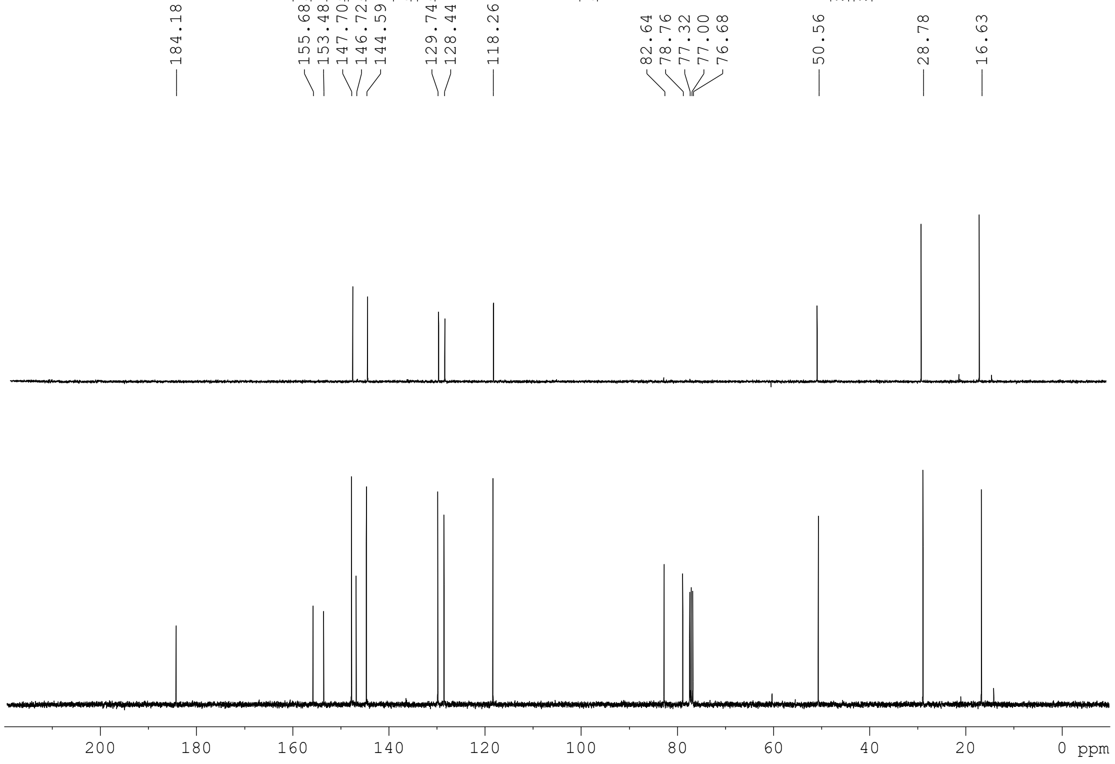

^{1}H NMR ($CDCl_3$, 400 MHz), ^{13}C NMR ($CDCl_3$, 100 MHz), and DEPT 135 spectra of **3b**

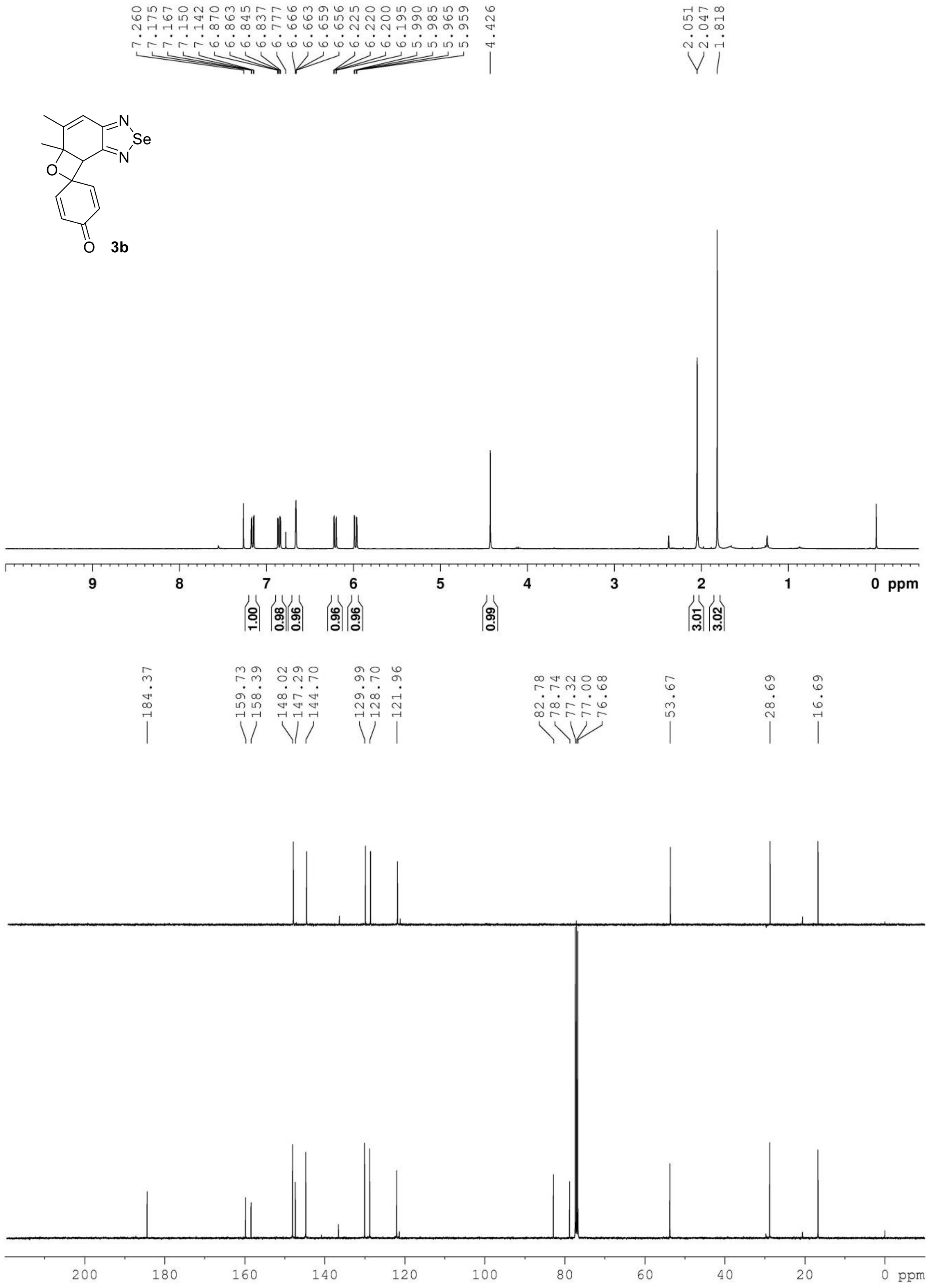

^{1}H NMR ($CDCl_3$, 400 MHz), ^{13}C NMR ($CDCl_3$, 100 MHz), and DEPT 135 spectra of **3c**

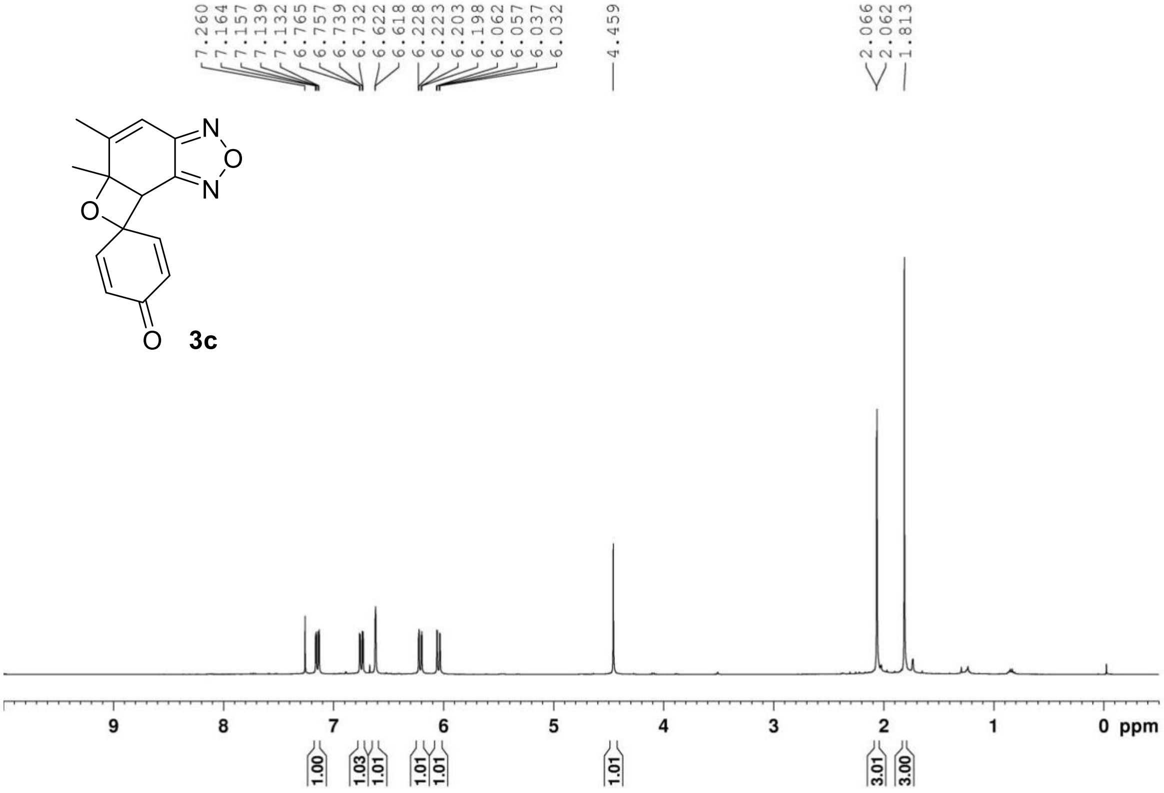

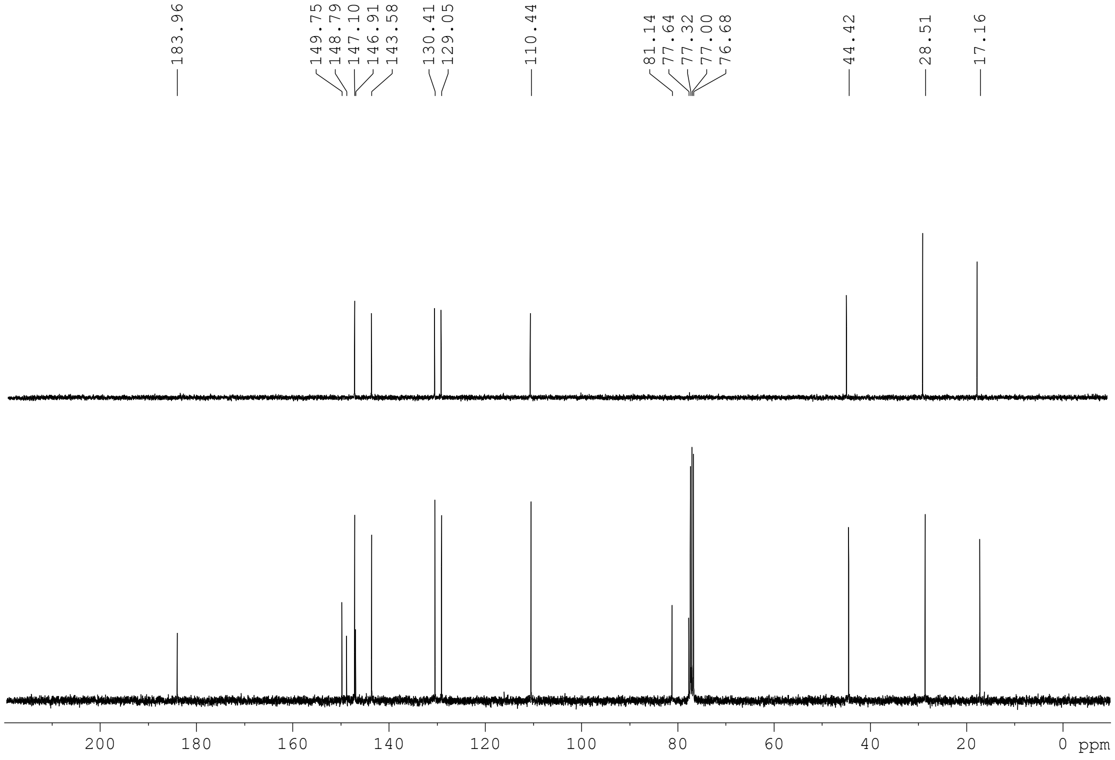

^{1}H NMR ($CDCl_3$, 400 MHz), ^{13}C NMR ($CDCl_3$, 100 MHz), and DEPT 135 spectra of **3d**

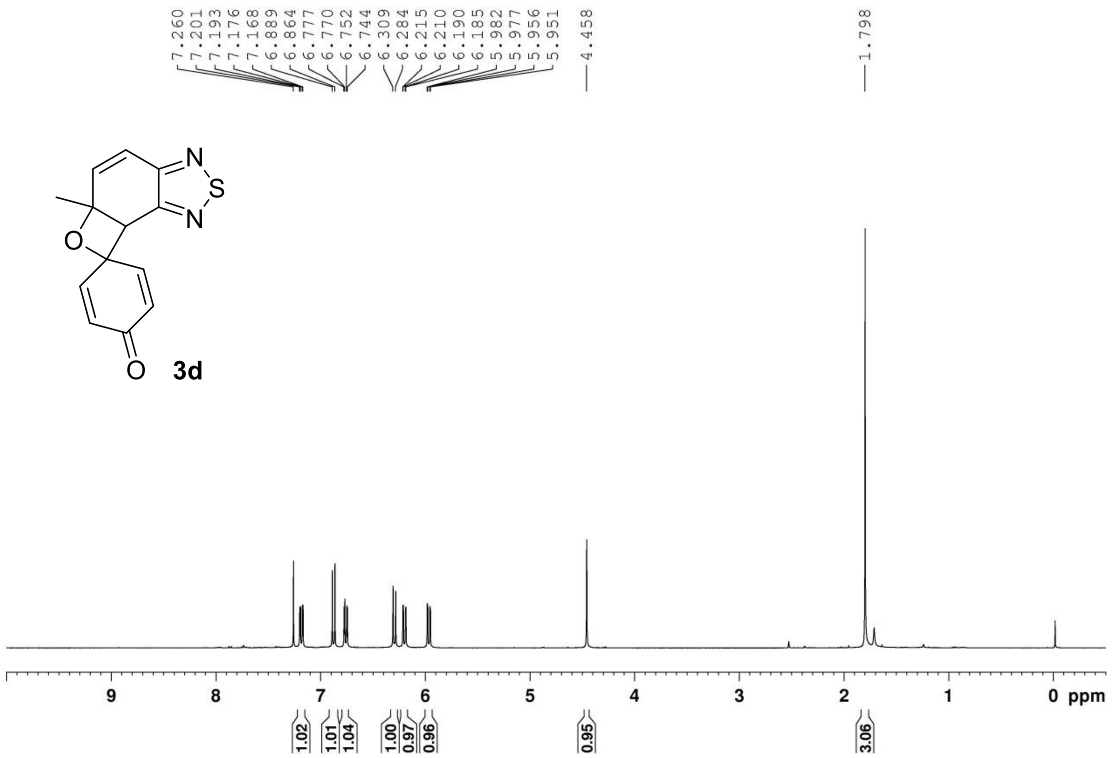

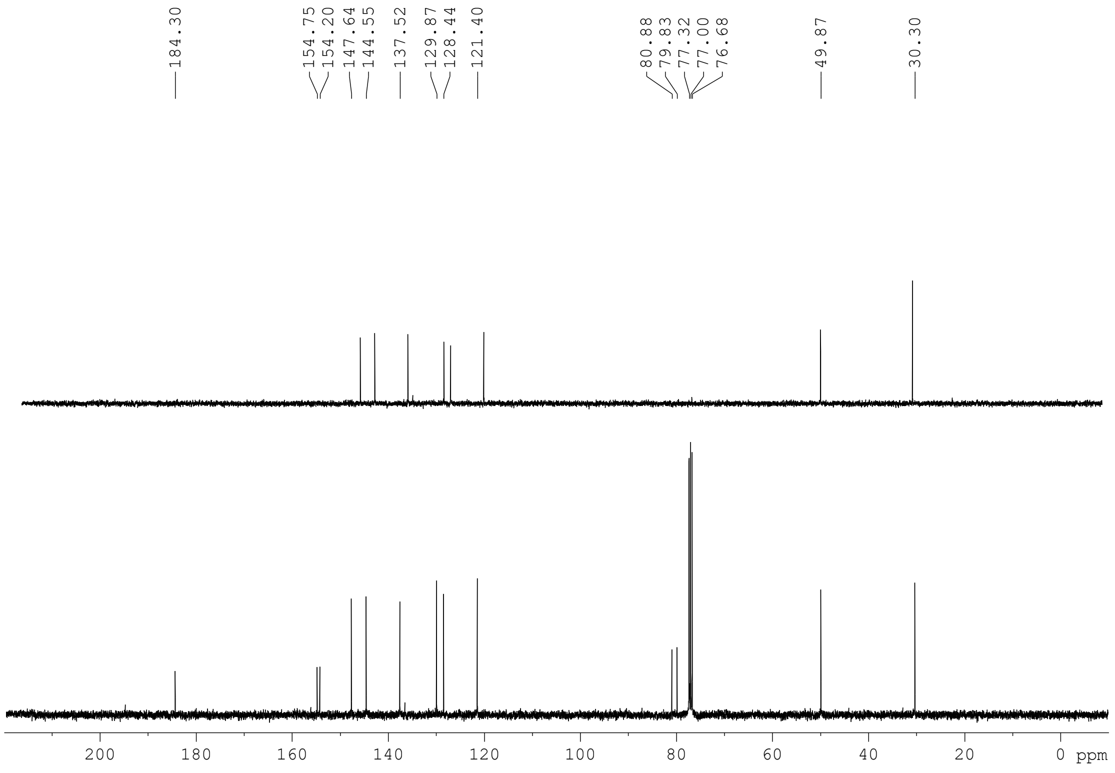

^{1}H NMR ($CDCl_3$, 400 MHz), ^{13}C NMR ($CDCl_3$, 100 MHz), and DEPT 135 spectra of **3e**

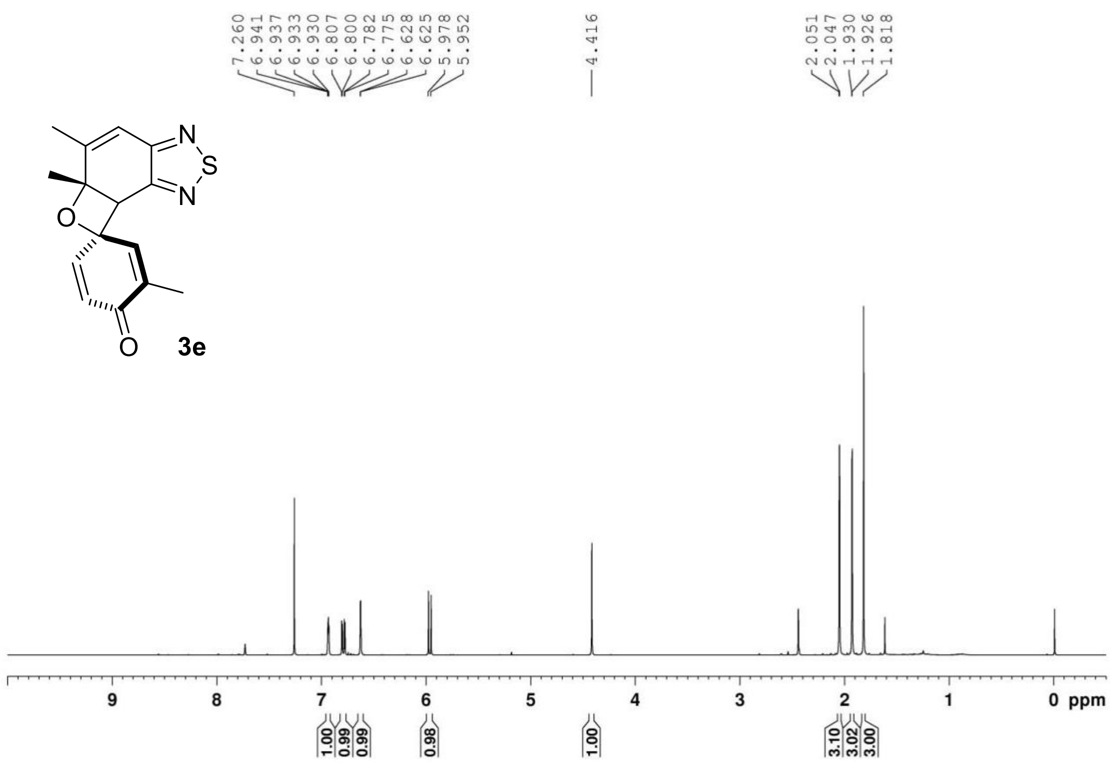

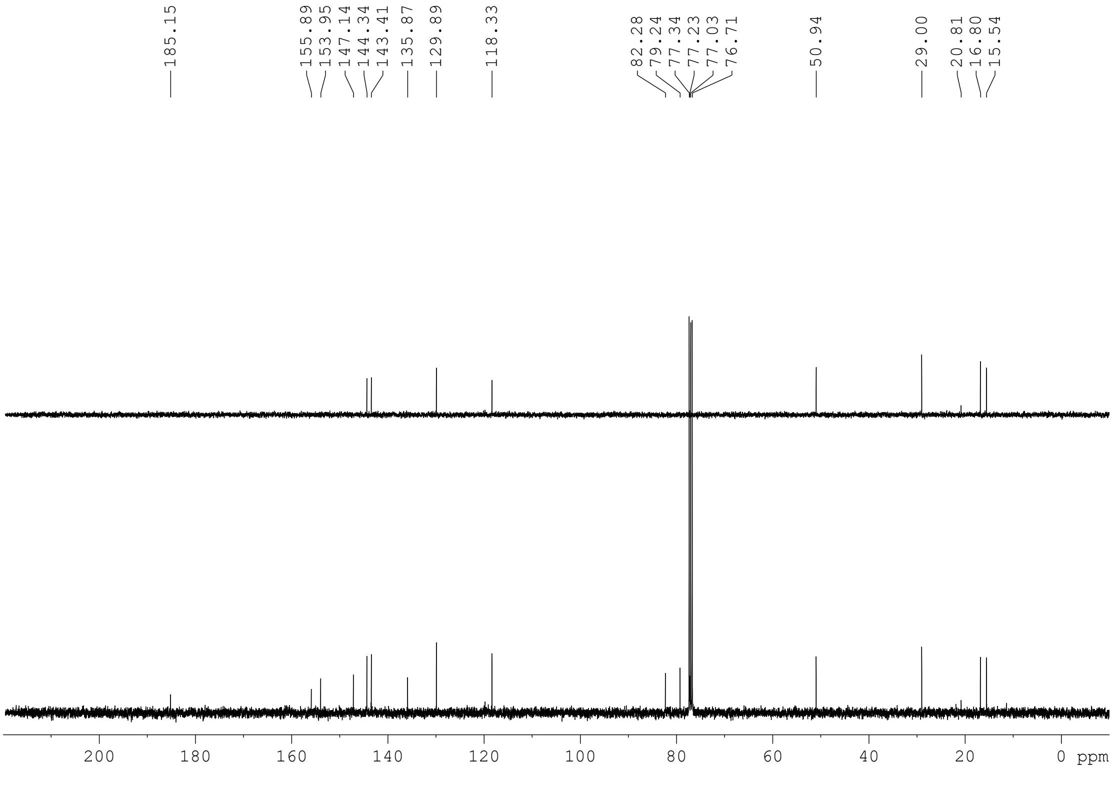

NOESY NMR ($CDCl_3$, 400 MHz) of **3e**

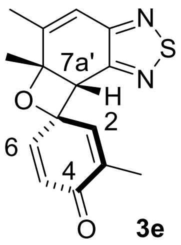

3e

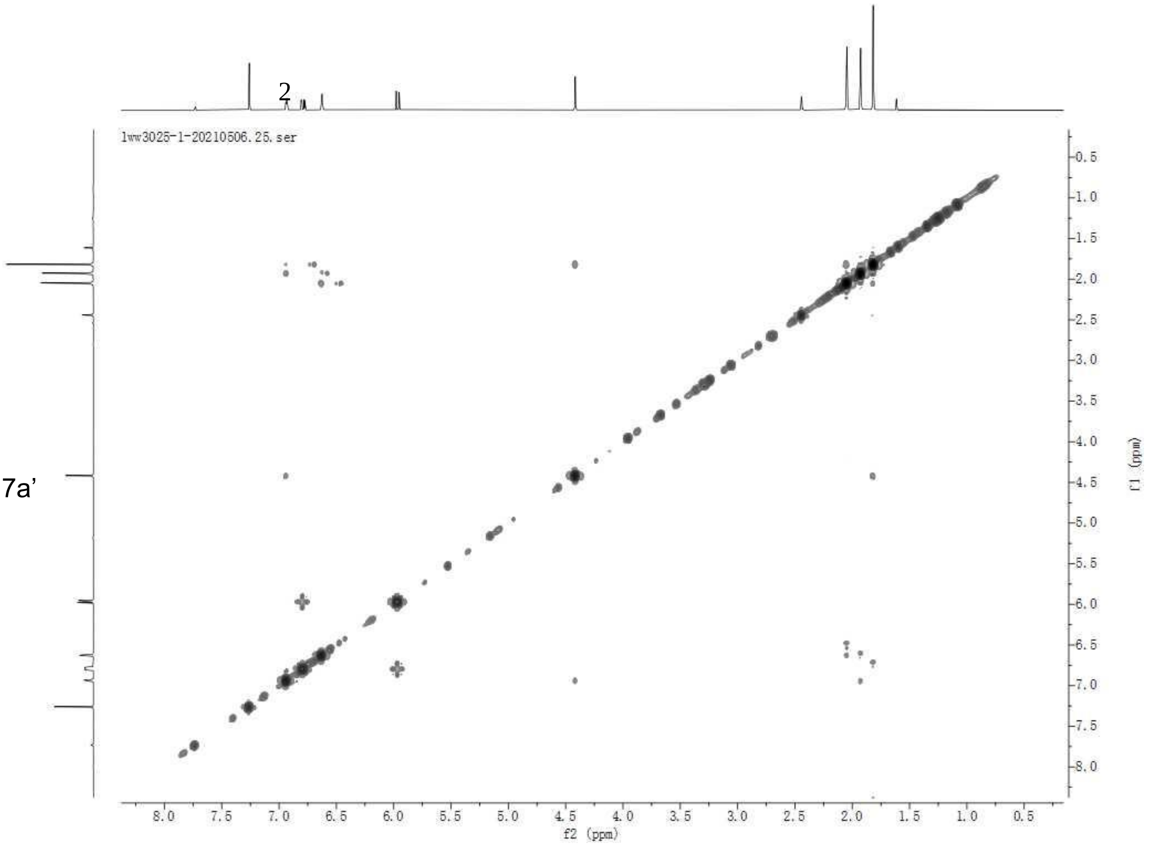

^{1}H NMR ($CDCl_3$, 400 MHz), ^{13}C NMR ($CDCl_3$, 100 MHz), and DEPT 135 spectra of **3e'**

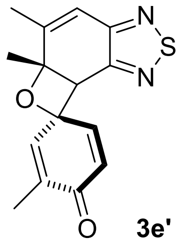

3e'

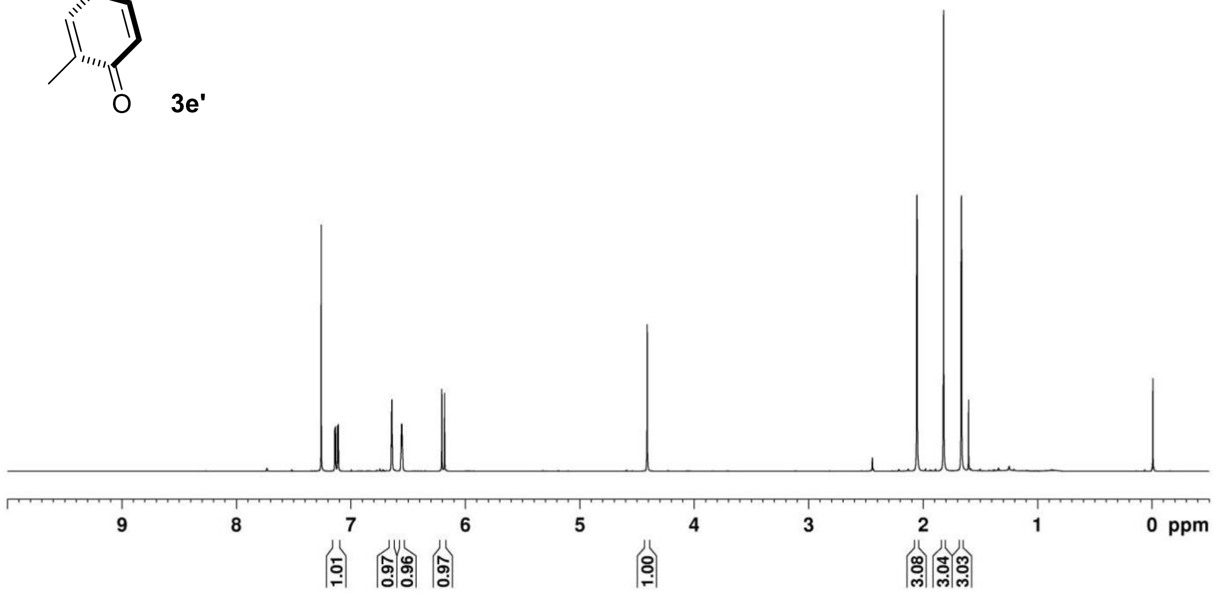

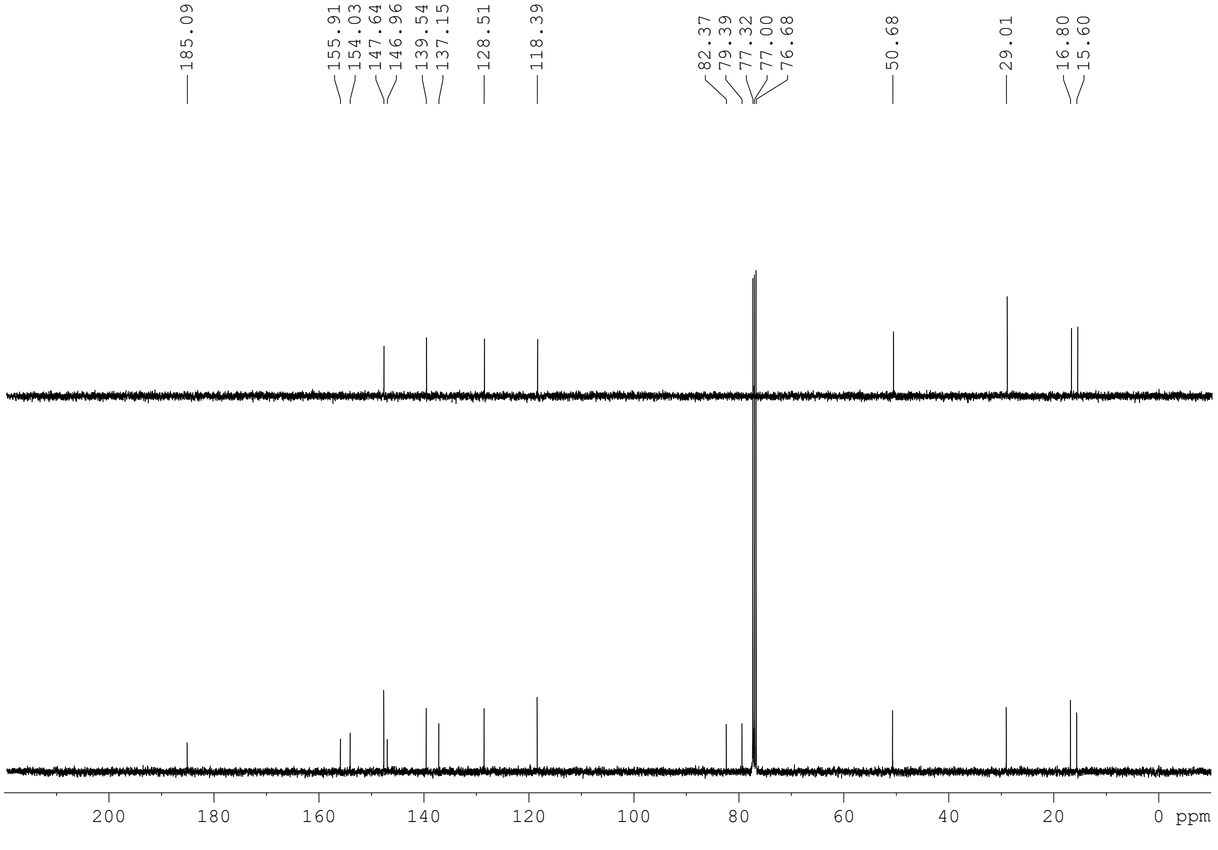

NOESY NMR ($CDCl_3$, 400 MHz) of **3e'**

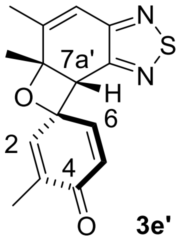

3e'

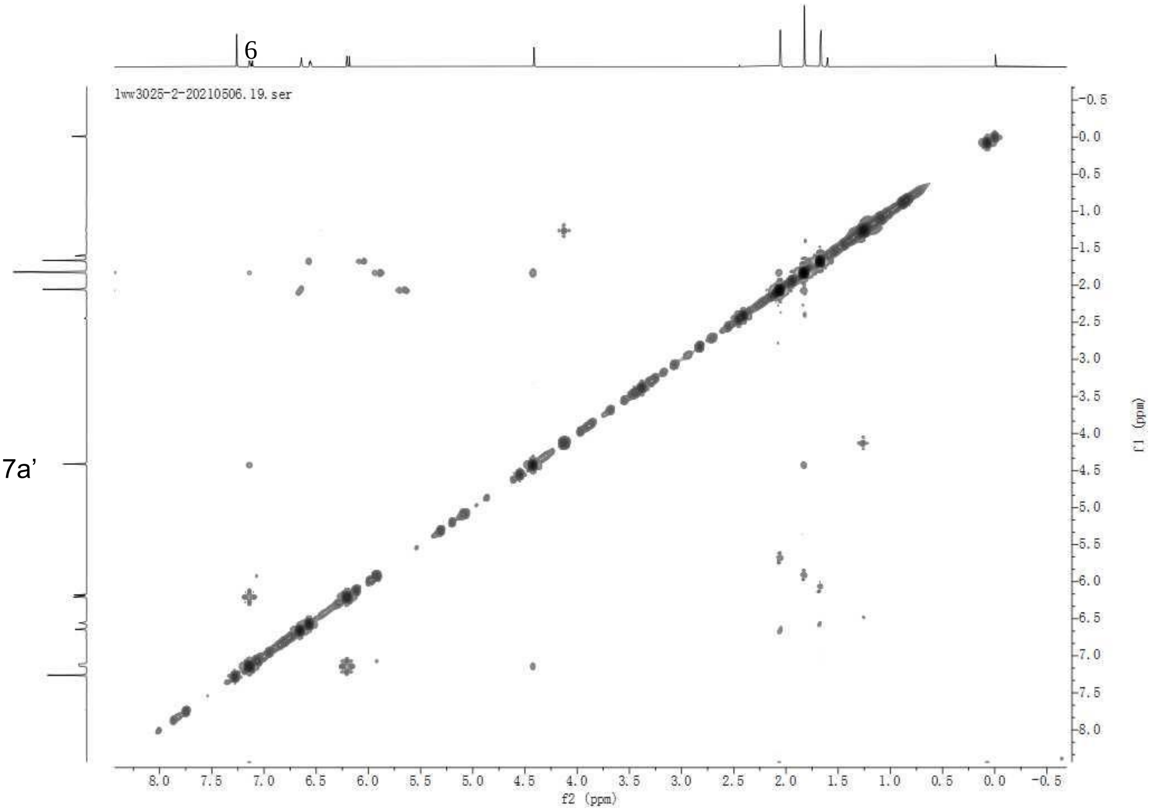

^{1}H NMR ($CDCl_3$, 400 MHz), ^{13}C NMR ($CDCl_3$, 100 MHz), and DEPT 135 spectra of **3f**

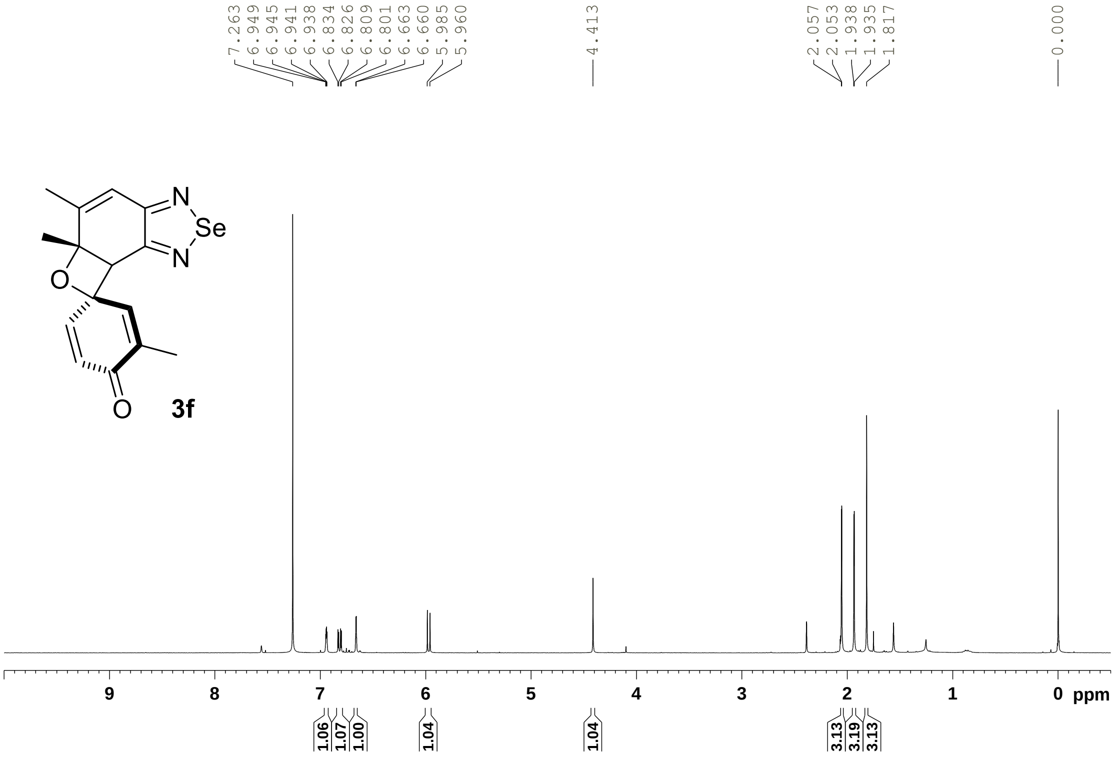

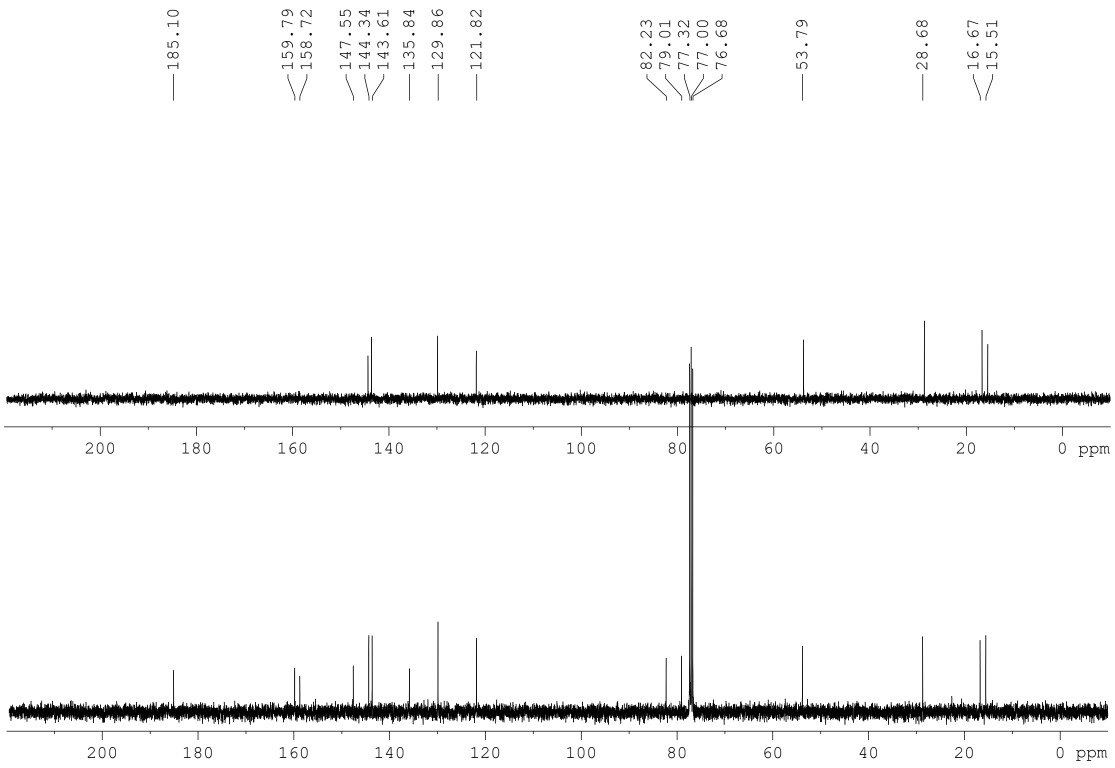

^{1}H NMR ($CDCl_3$, 400 MHz), ^{13}C NMR ($CDCl_3$, 100 MHz), and DEPT 135 spectra of **3f'**

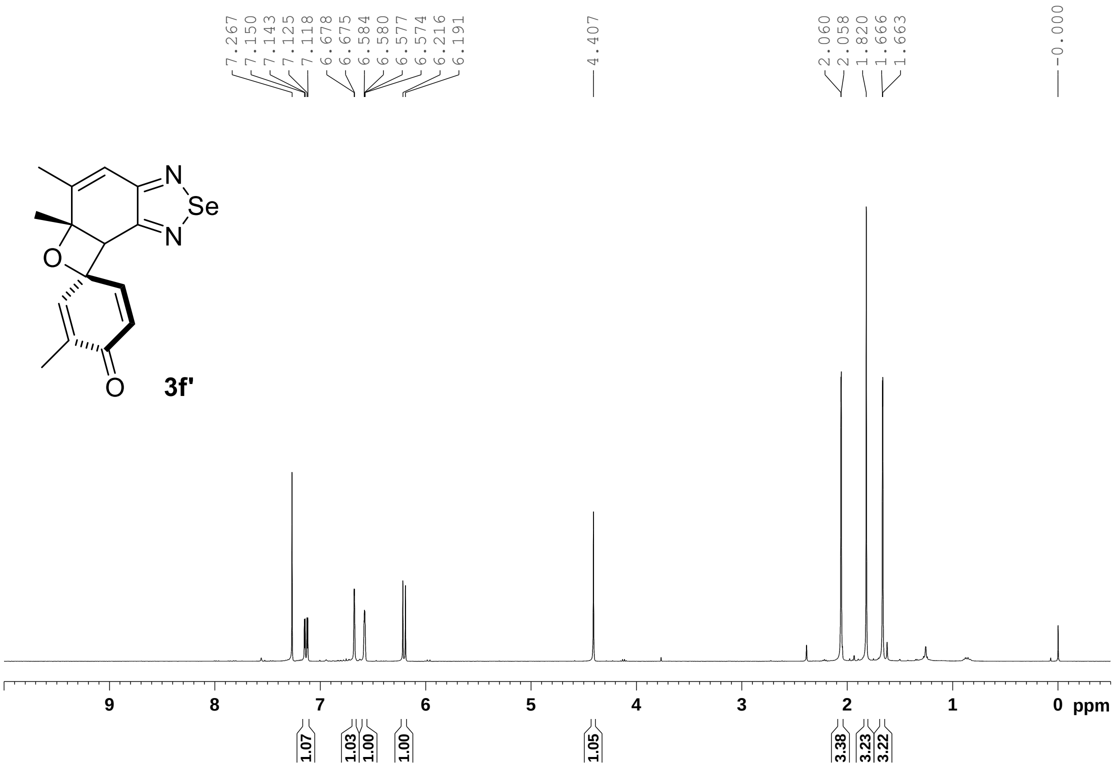

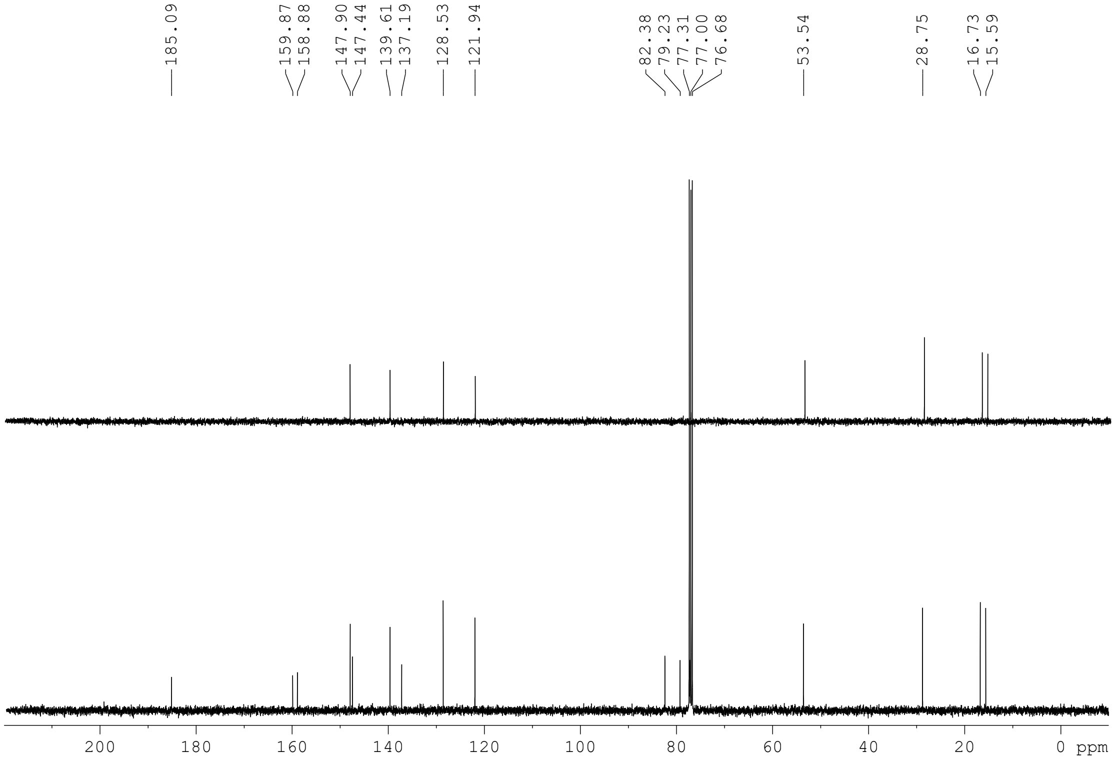

^{1}H NMR ($CDCl_3$, 400 MHz), ^{13}C NMR ($CDCl_3$, 100 MHz), and DEPT 135 spectra of **3g**

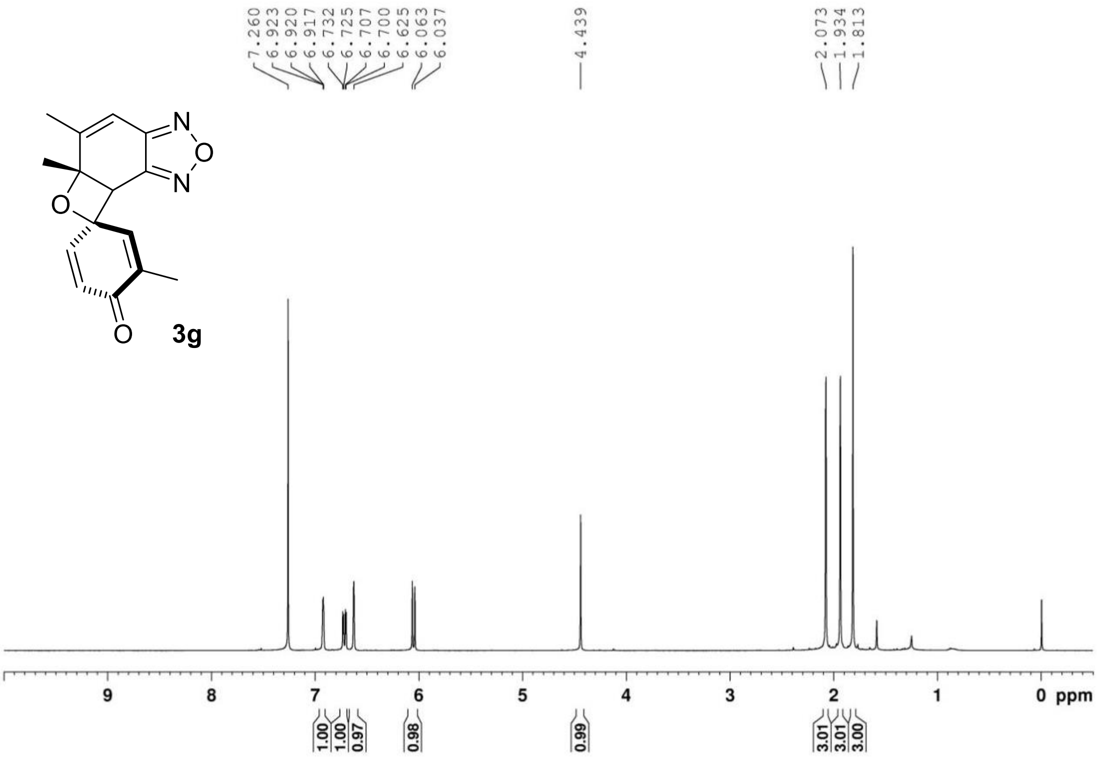

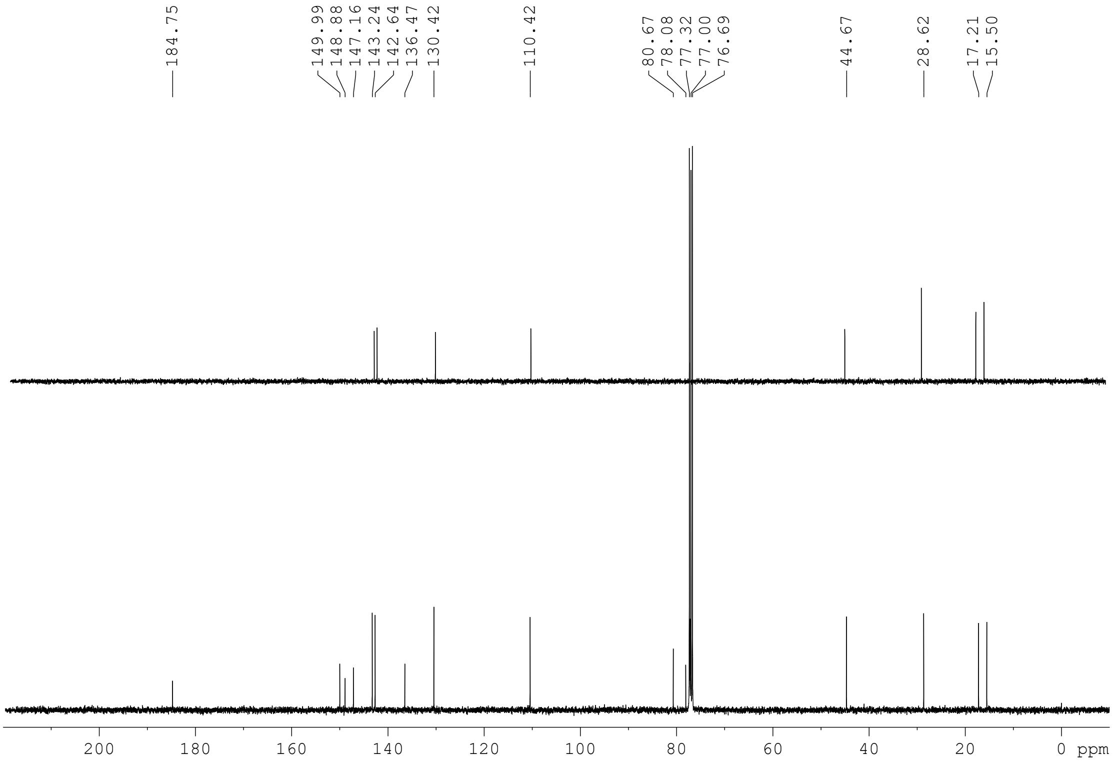

^{1}H NMR ($CDCl_3$, 400 MHz), ^{13}C NMR ($CDCl_3$, 100 MHz), and DEPT 135 spectra of **3h**

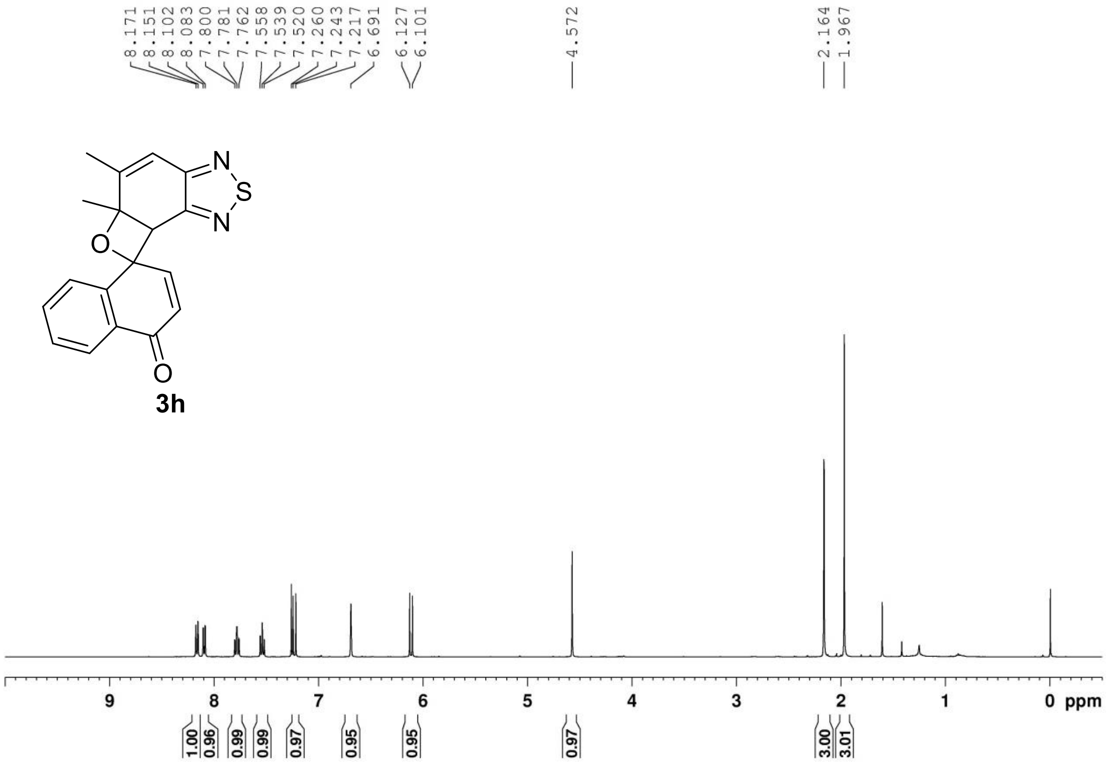

3h

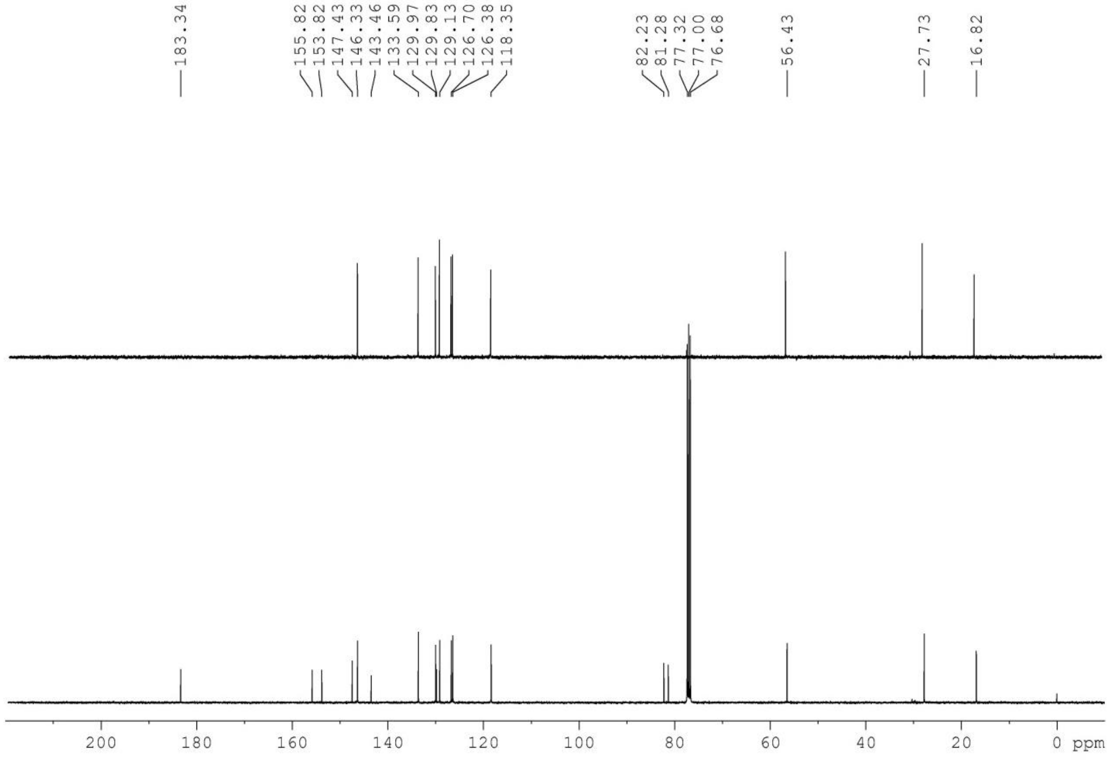

^{1}H NMR (CD_3COCD_3, 400 MHz), ^{13}C NMR (CD_3COCD_3, 100 MHz), and DEPT 135 spectra of **4a**

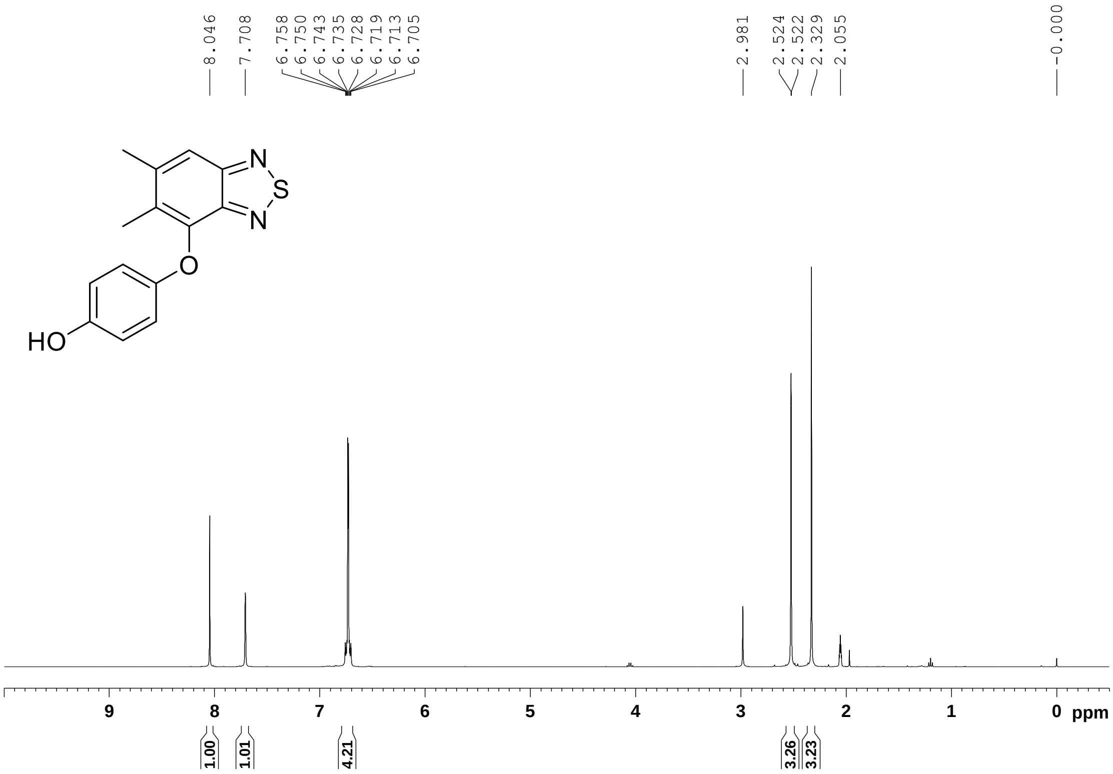

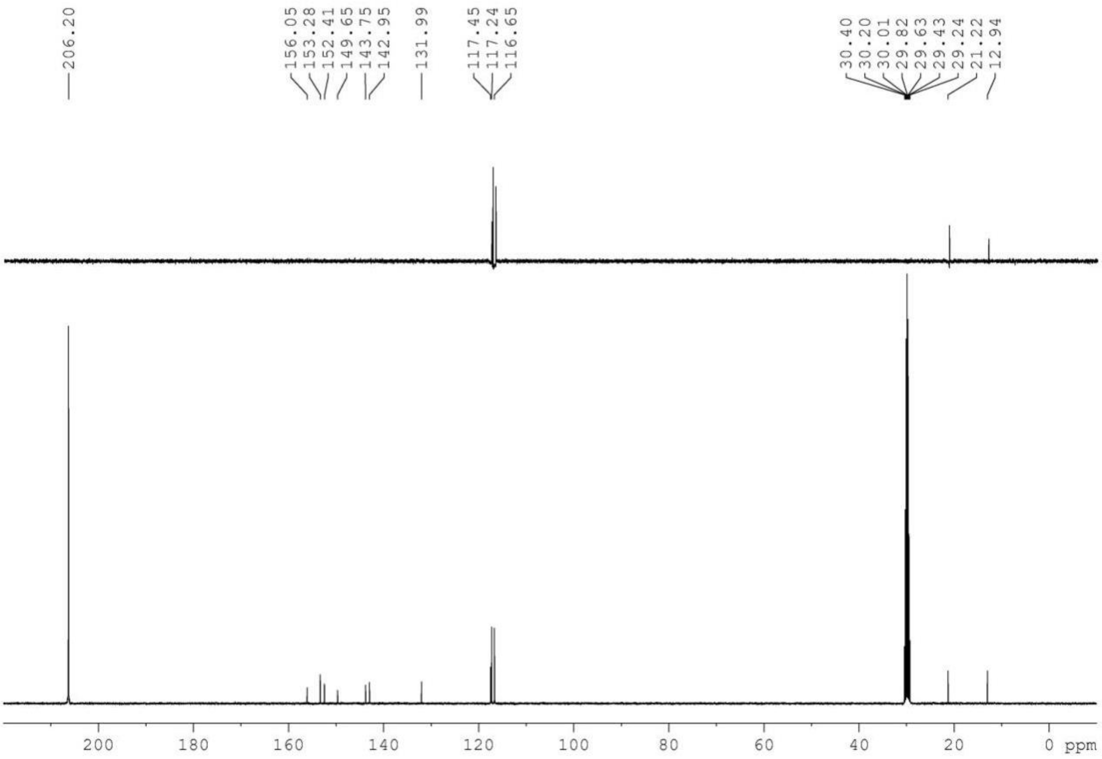

^{1}H NMR (CD_3COCD_3, 400 MHz), ^{13}C NMR (CD_3COCD_3, 100 MHz), and DEPT 135 spectra of **4b**

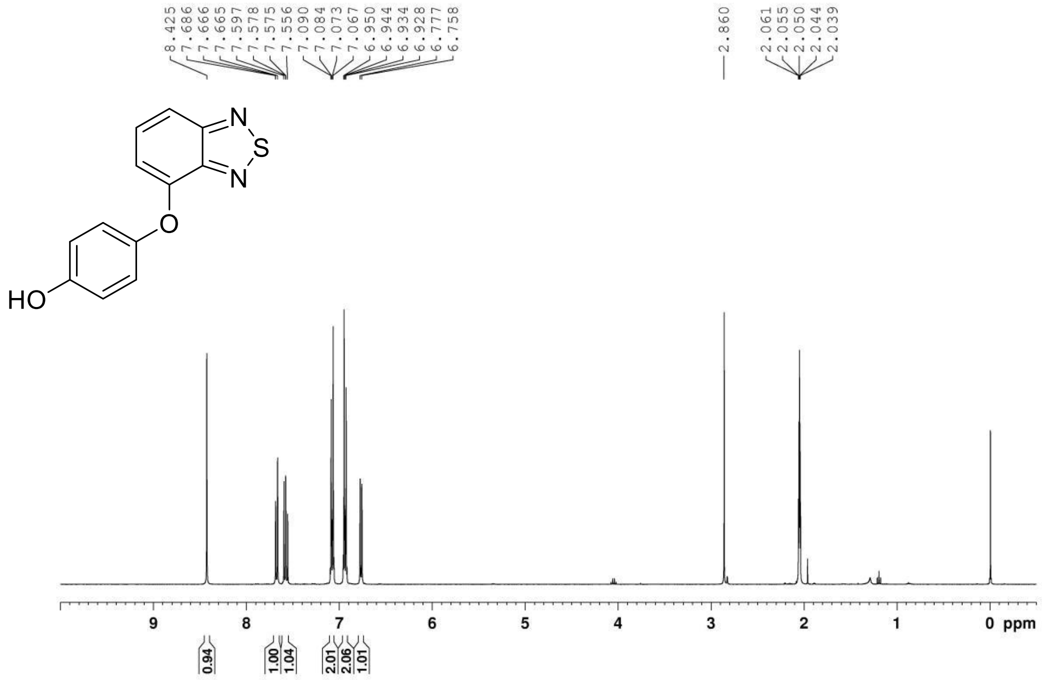

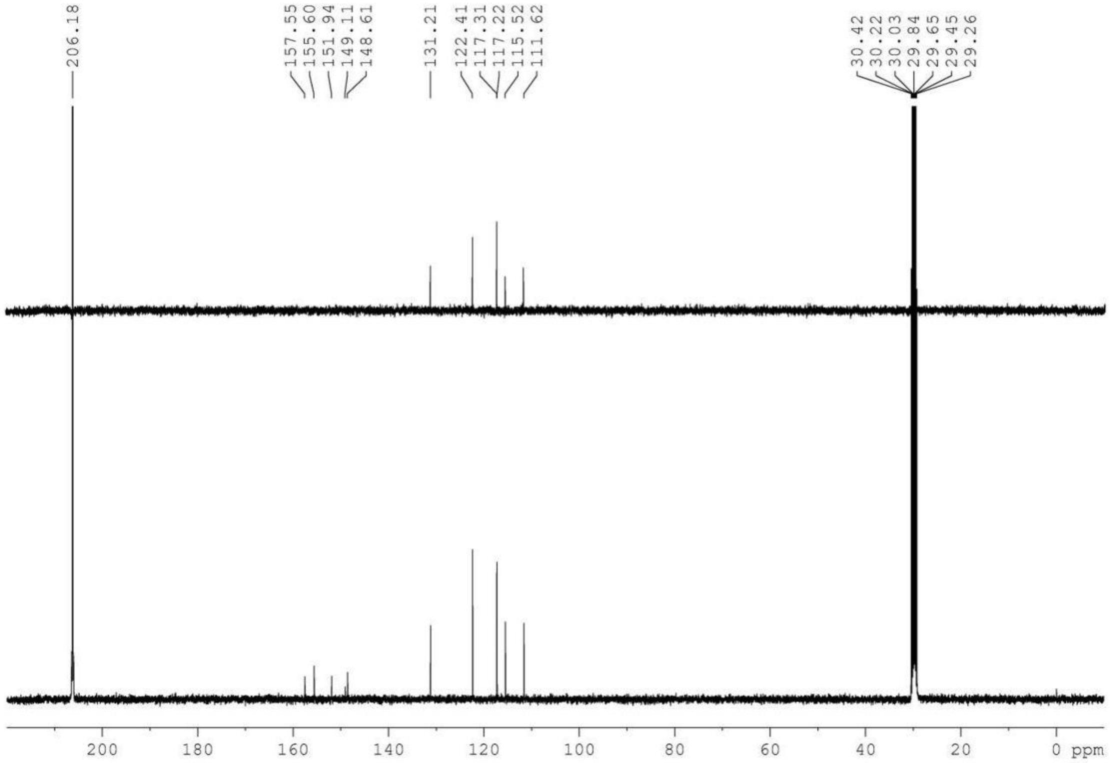

^{1}H NMR (CD_3COCD_3, 400 MHz), ^{13}C NMR (CD_3COCD_3, 100 MHz), and DEPT 135 spectra of **4c**

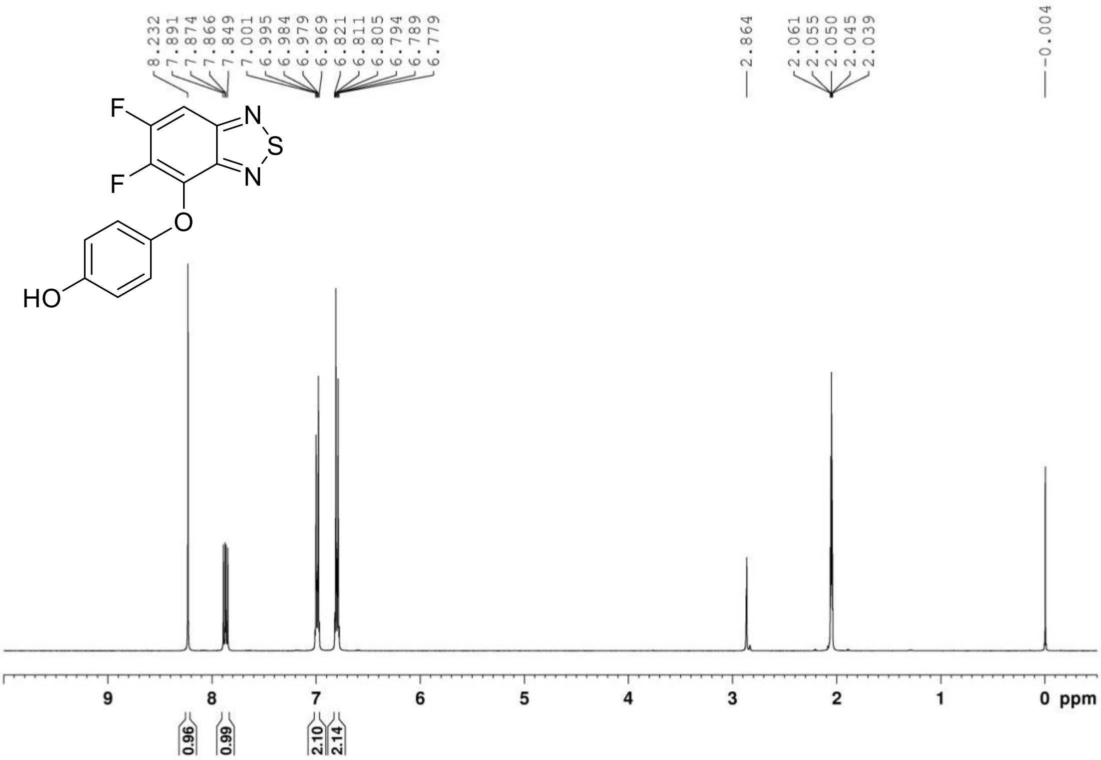

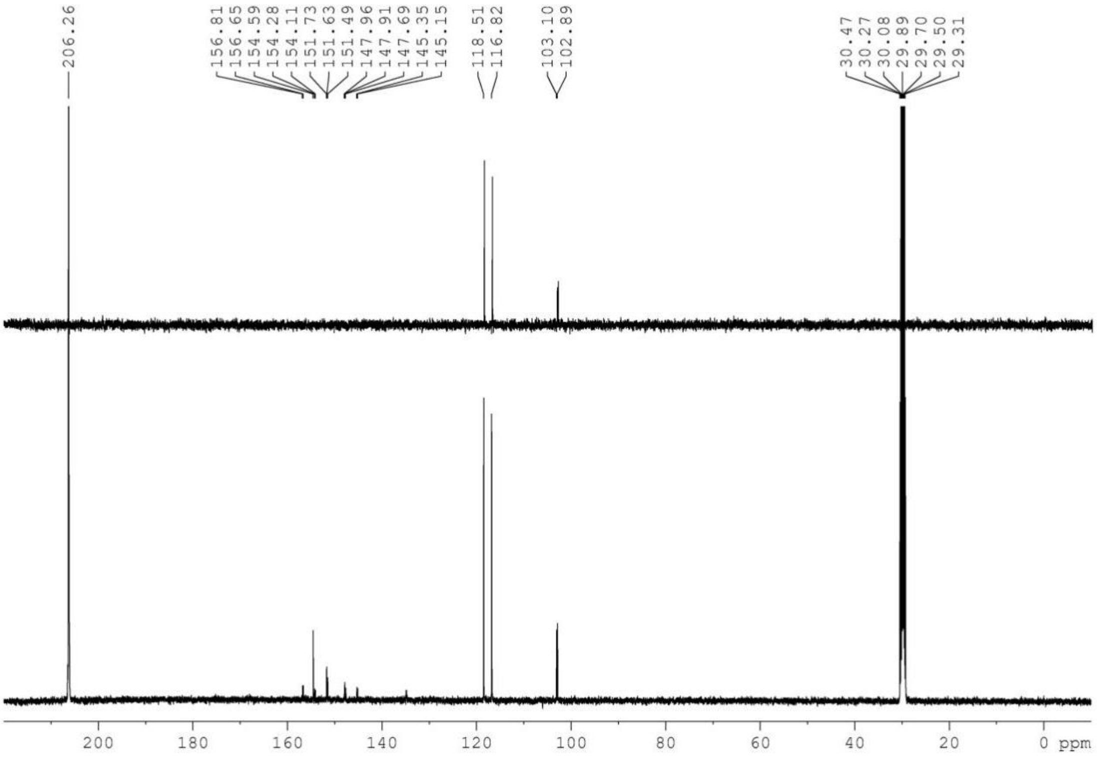

^{19}F NMR (CD_3COCD_3, 100 MHz) spectrum of **4c**

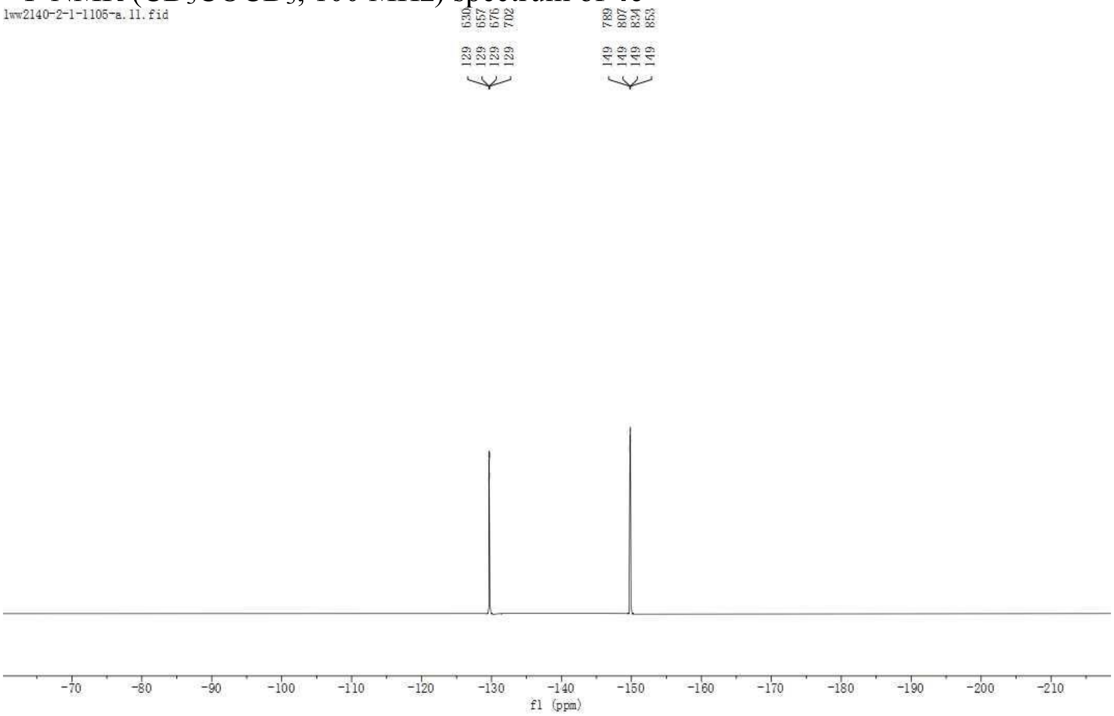

^{1}H NMR (CD_3COCD_3, 400 MHz), ^{13}C NMR (CD_3COCD_3, 100 MHz), and DEPT 135 spectra of **4d**

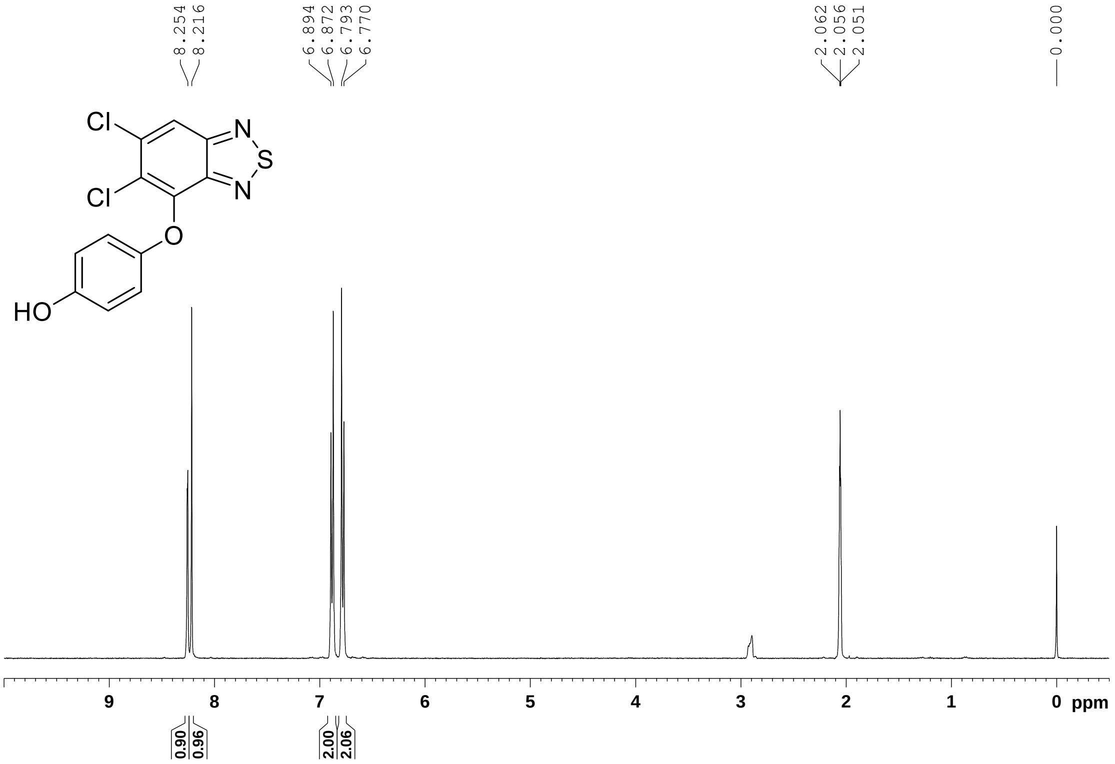

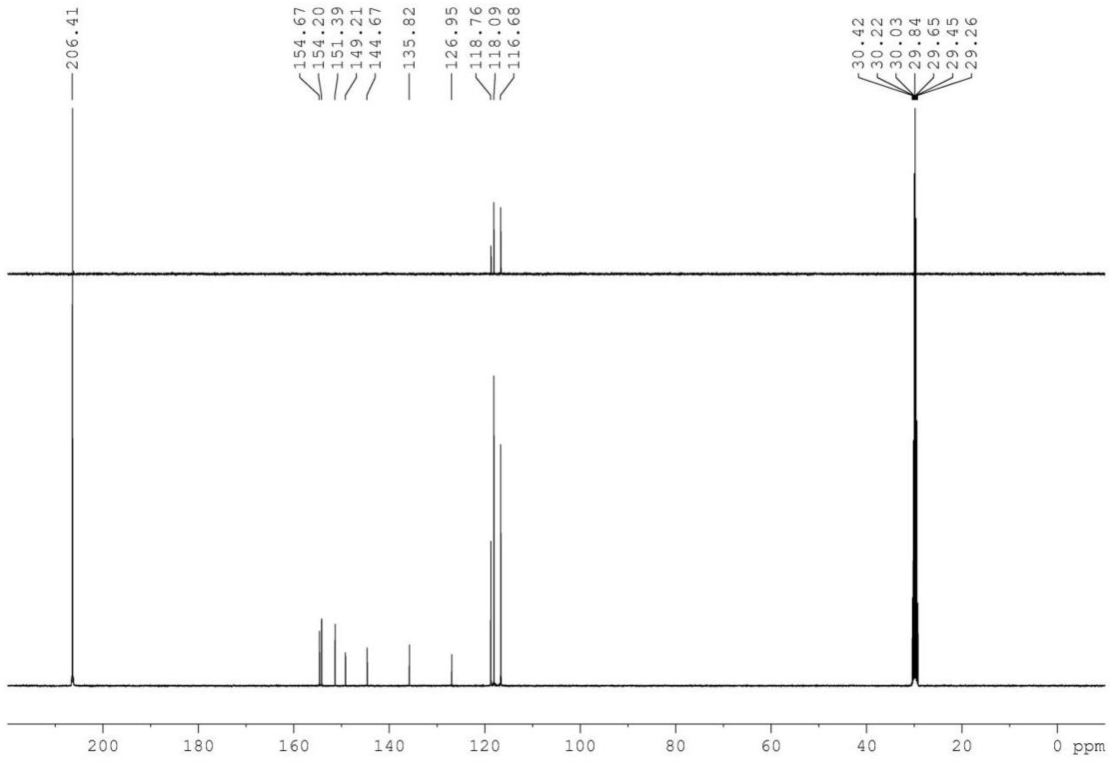

^{1}H NMR (CD_3COCD_3, 400 MHz), ^{13}C NMR (CD_3COCD_3, 100 MHz), and DEPT 135 spectra of **4e**

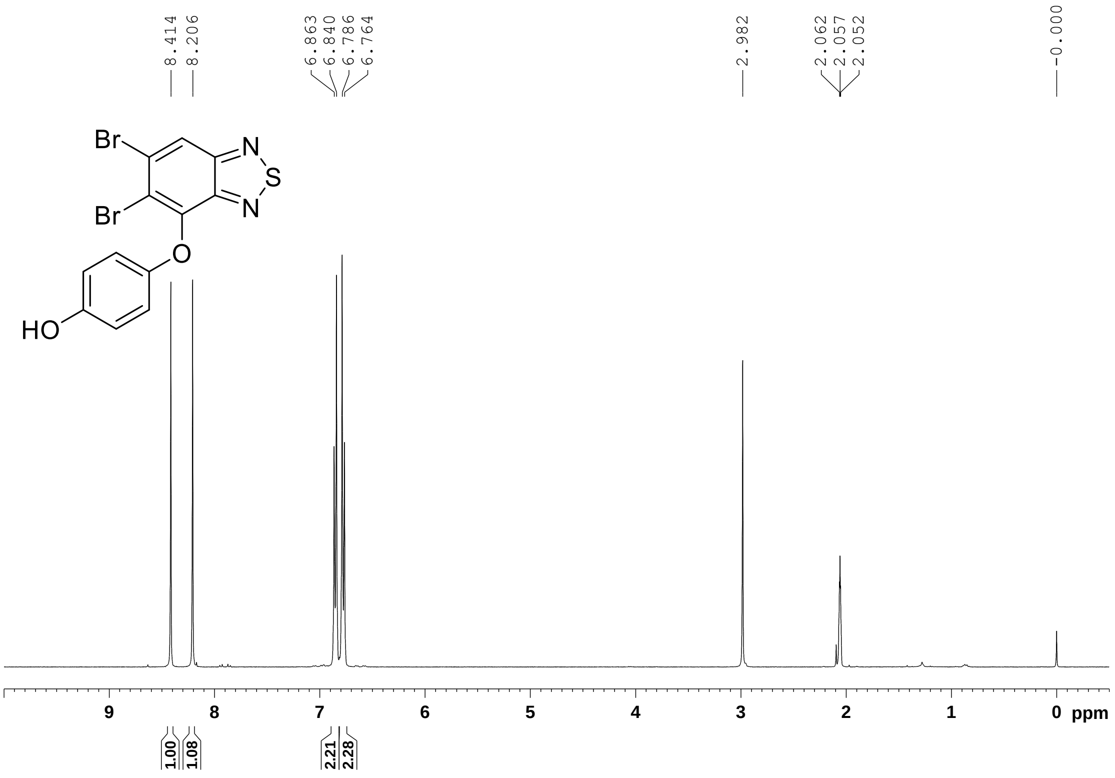

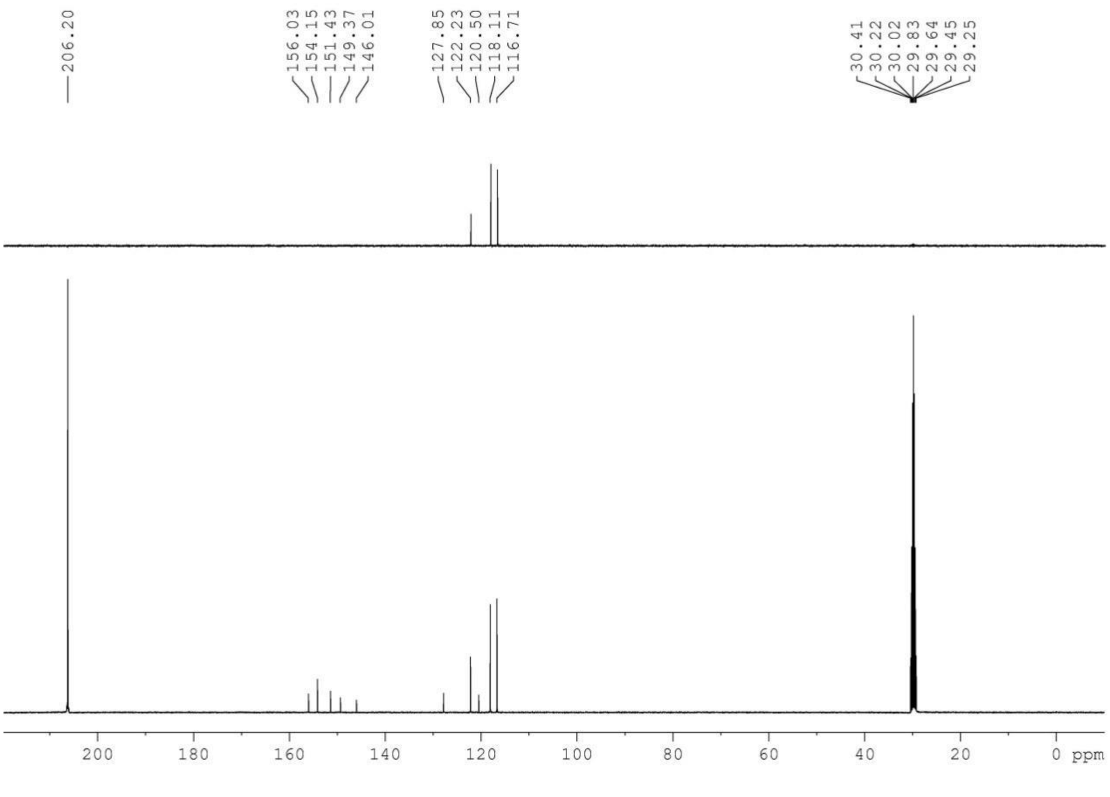

^{1}H NMR ($CDCl_3$, 400 MHz), ^{13}C NMR ($CDCl_3$, 100 MHz), and DEPT 135 spectra of **4f**

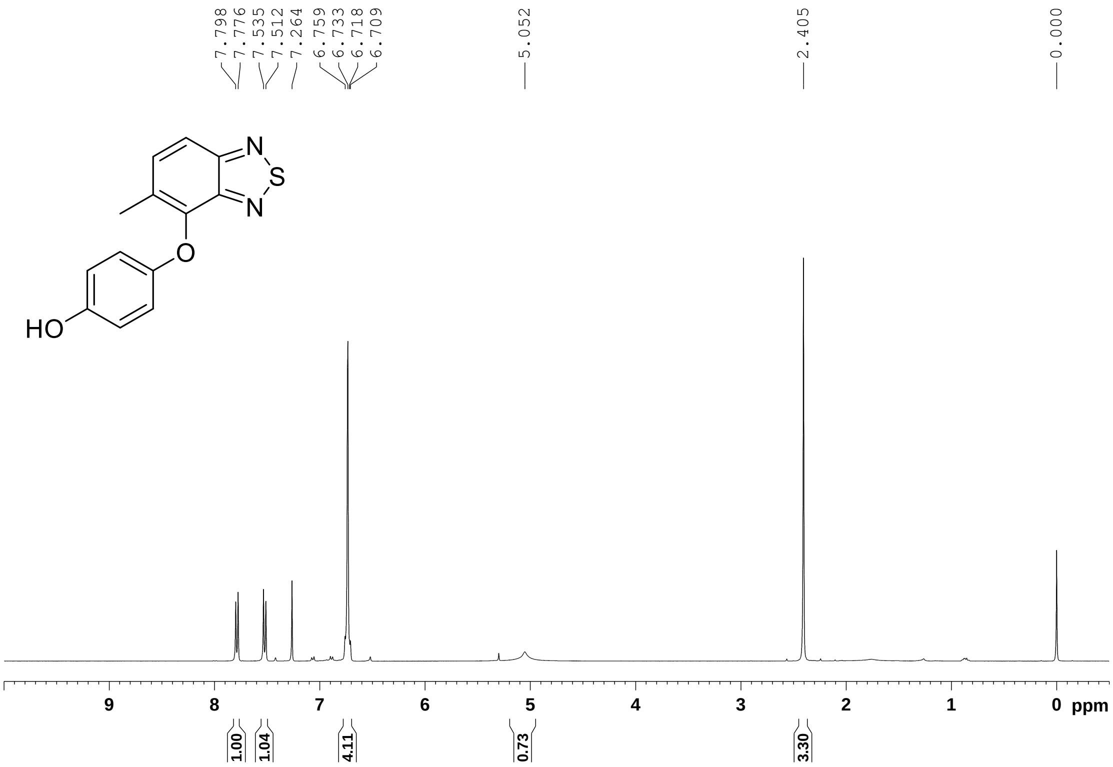

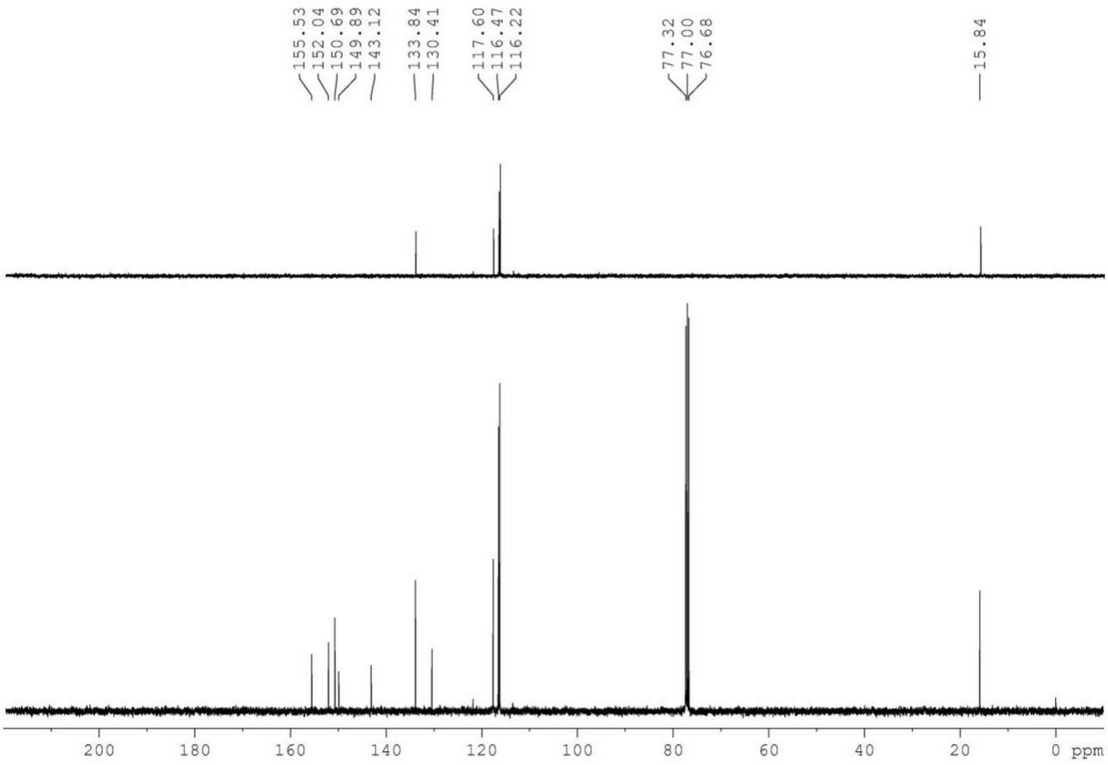

^{1}H NMR ($CDCl_3$, 400 MHz), ^{13}C NMR ($CDCl_3$, 100 MHz), and DEPT 135 spectra of **4g**

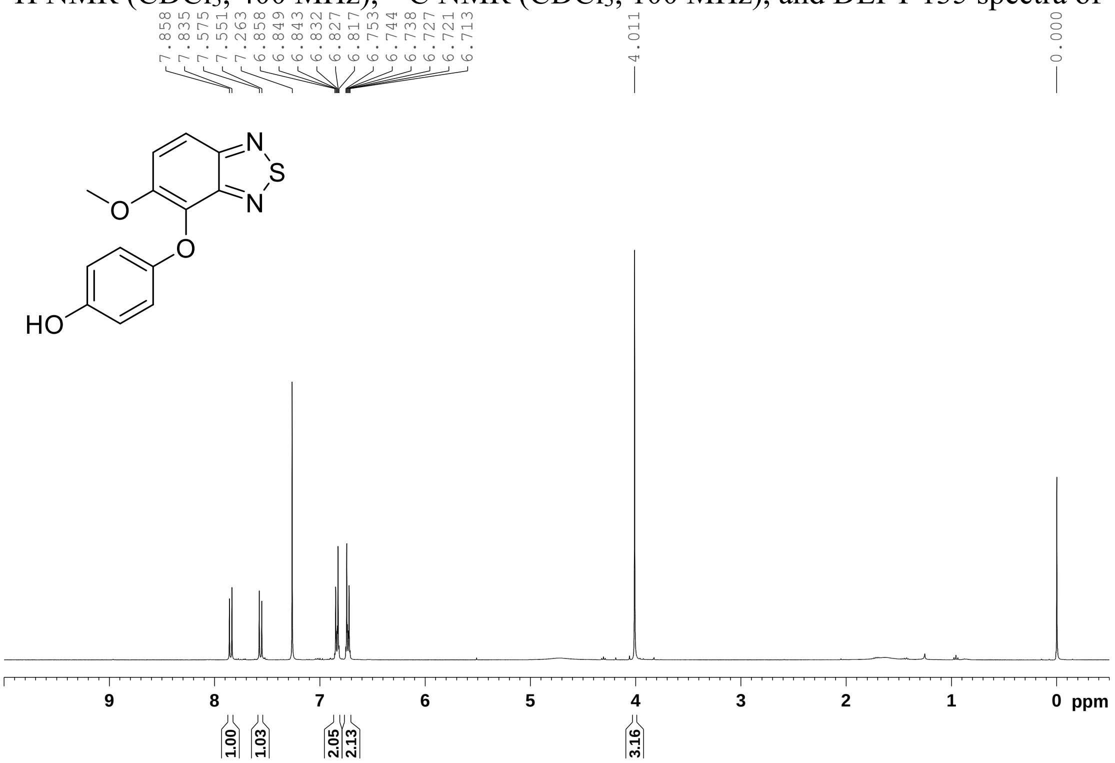

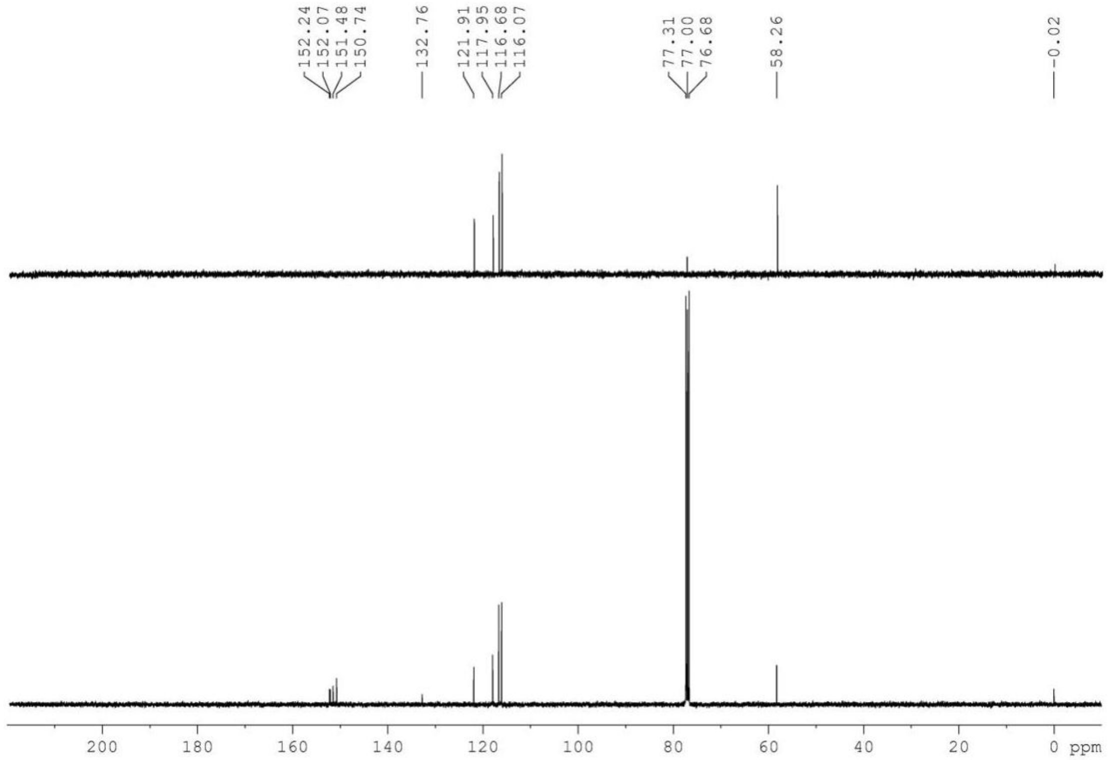

^{1}H NMR (CD_3COCD_3, 400 MHz), ^{13}C NMR (CD_3COCD_3, 100 MHz), and DEPT 135 spectra of **4h**

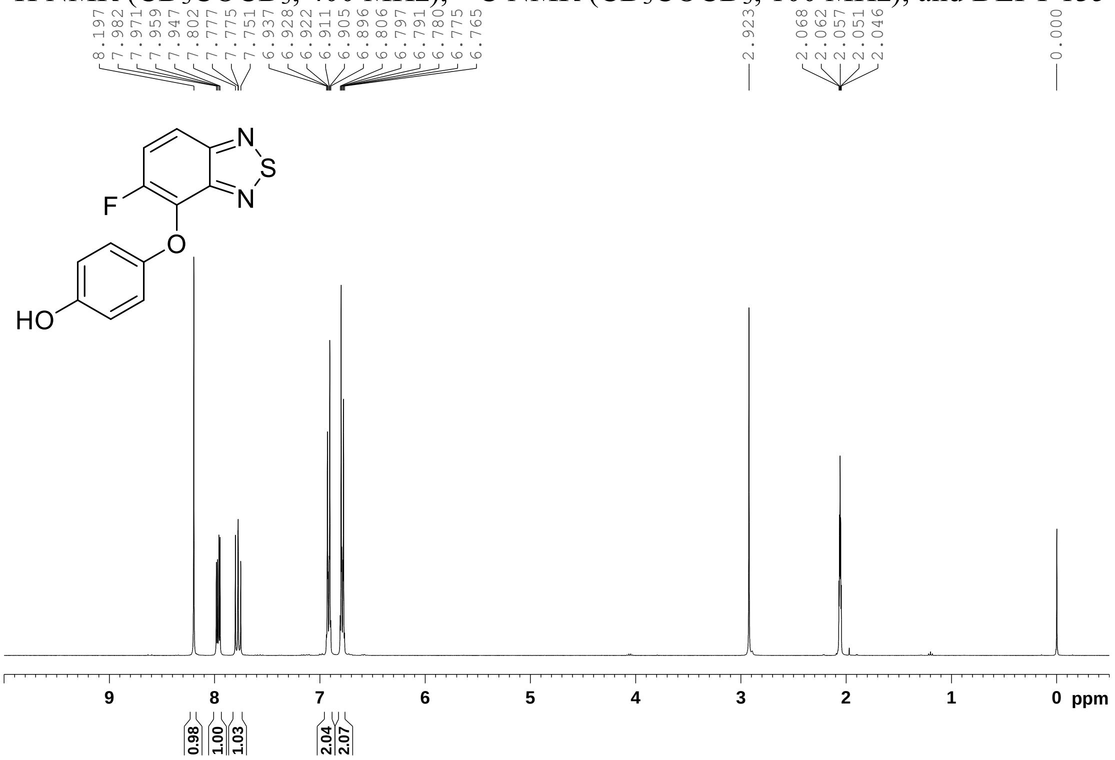

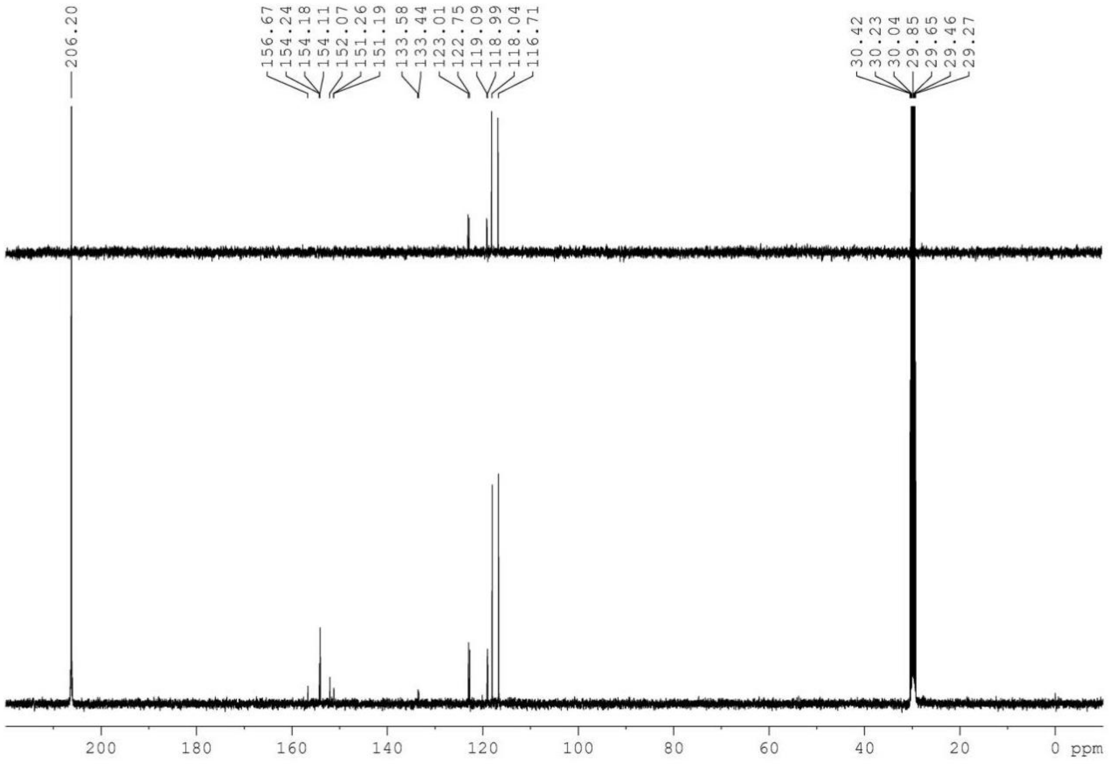

^{19}F NMR (CD_3COCD_3, 100 MHz) spectrum of **4h**

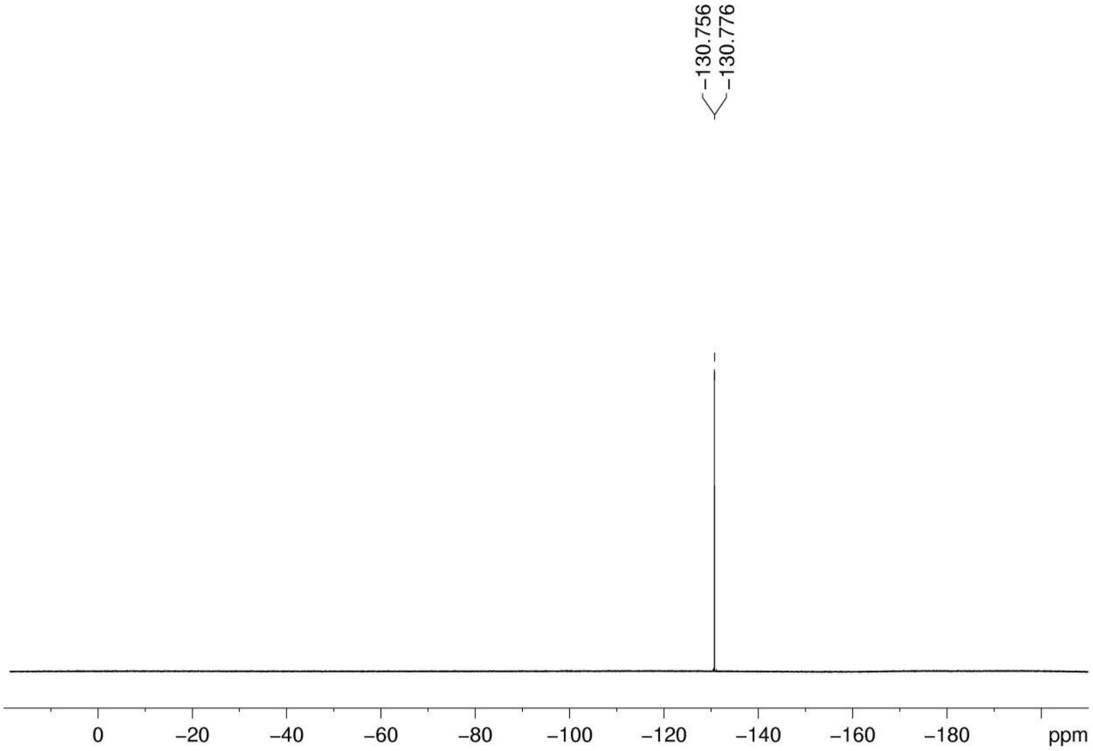

^{1}H NMR (CD_3COCD_3, 400 MHz), ^{13}C NMR (CD_3COCD_3, 100 MHz), and DEPT 135 spectra of **4h'**

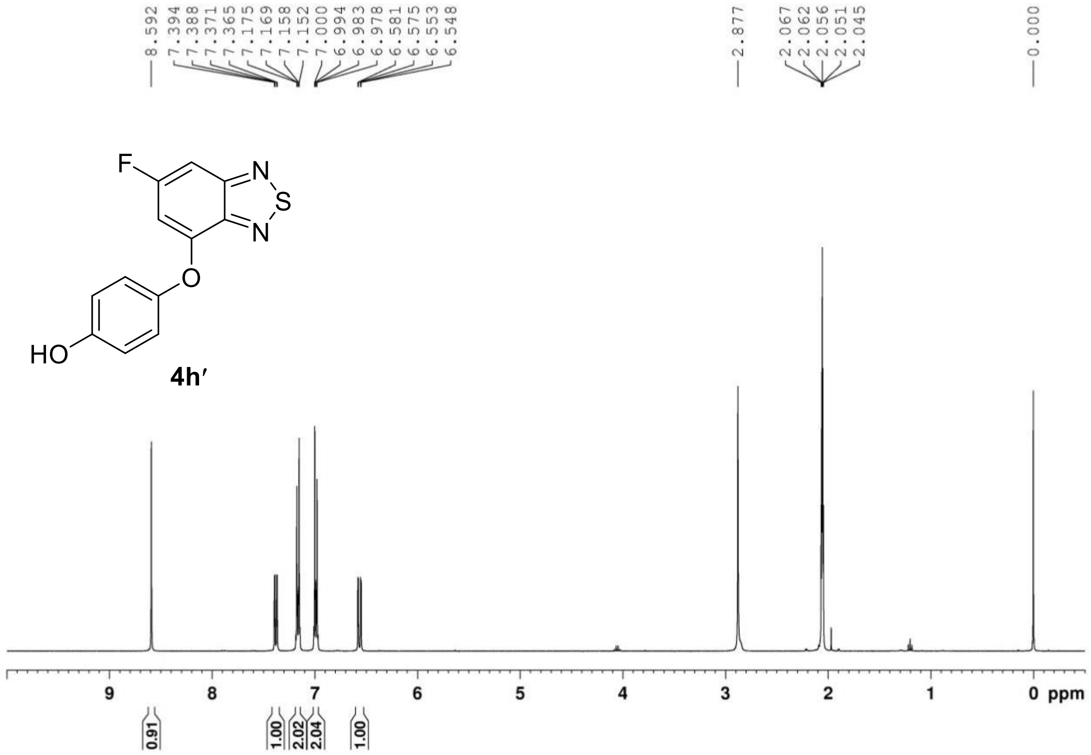

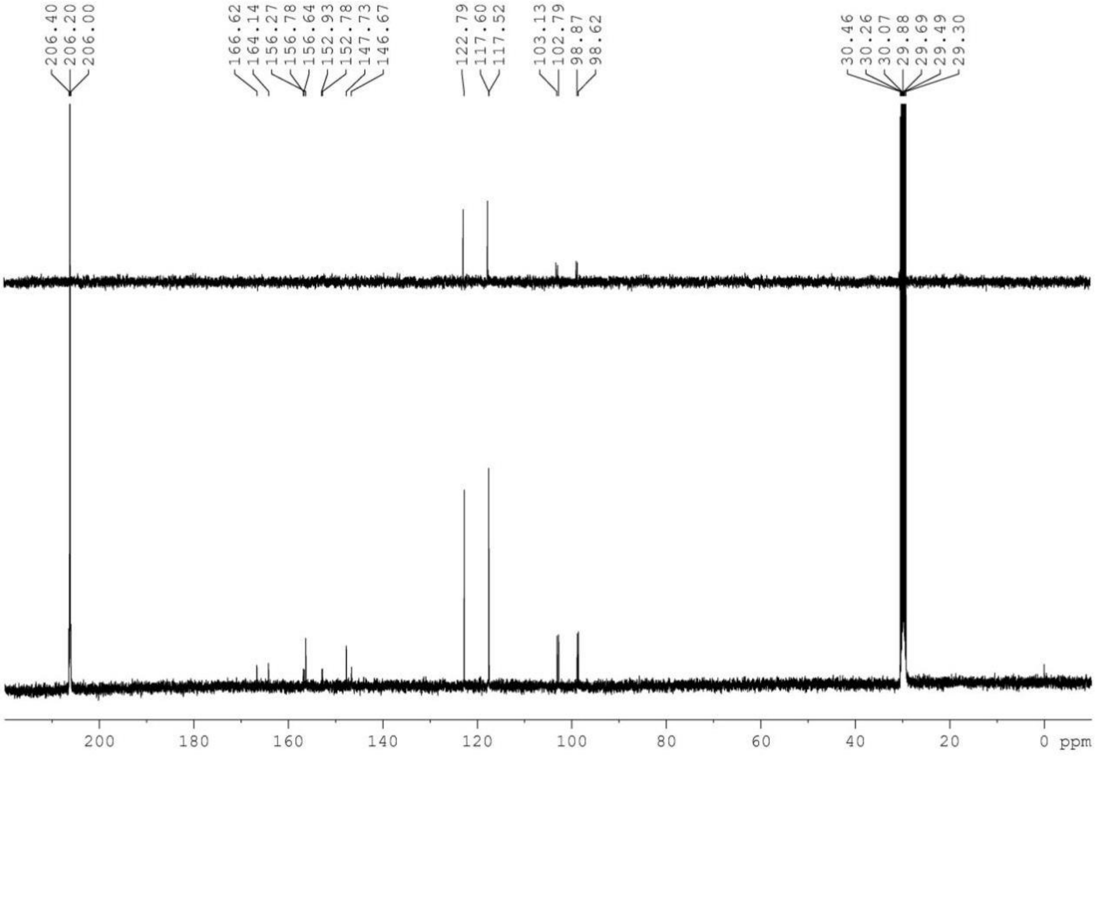

^{19}F NMR (CD_3COCD_3, 100 MHz) spectrum of **4h'**

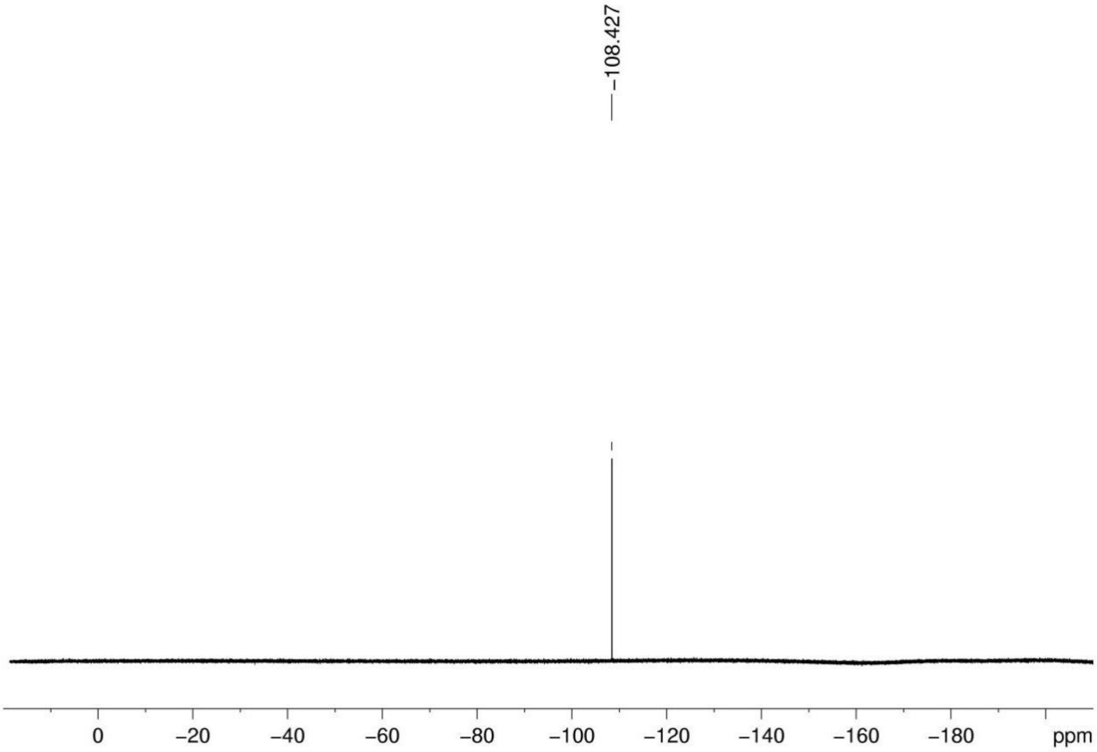

^{1}H NMR (CD_3COCD_3, 400 MHz), ^{13}C NMR (CD_3COCD_3, 100 MHz), and DEPT 135 spectra of **4i**

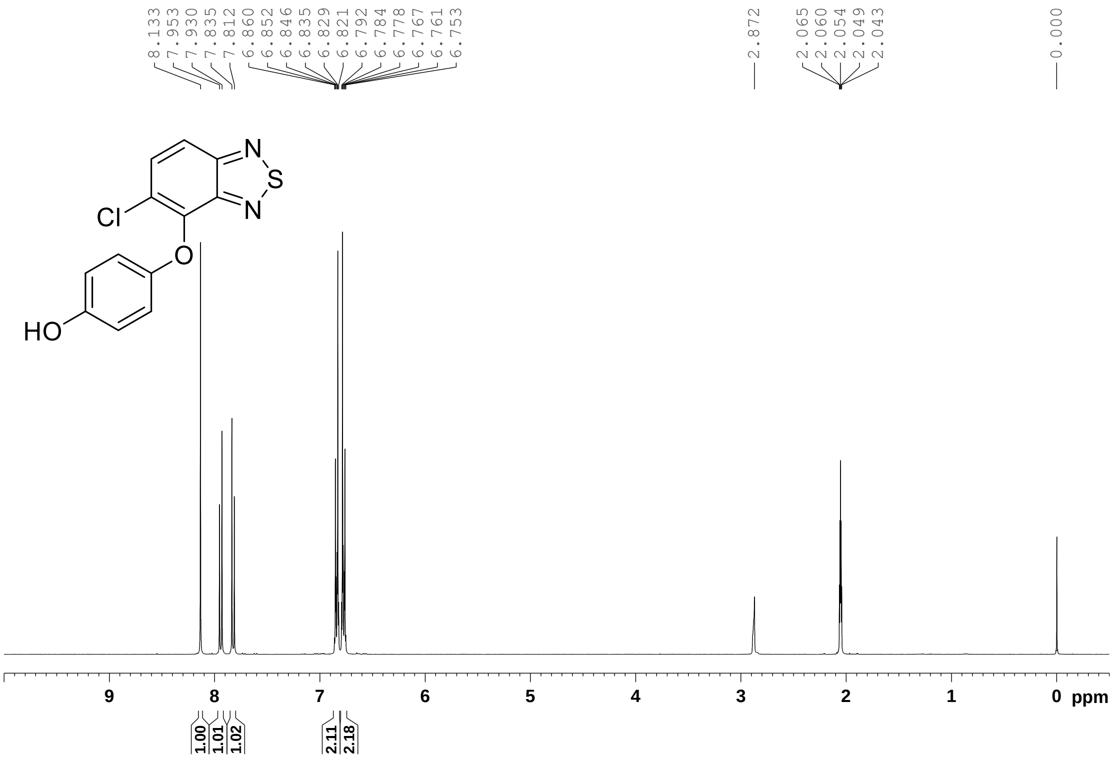

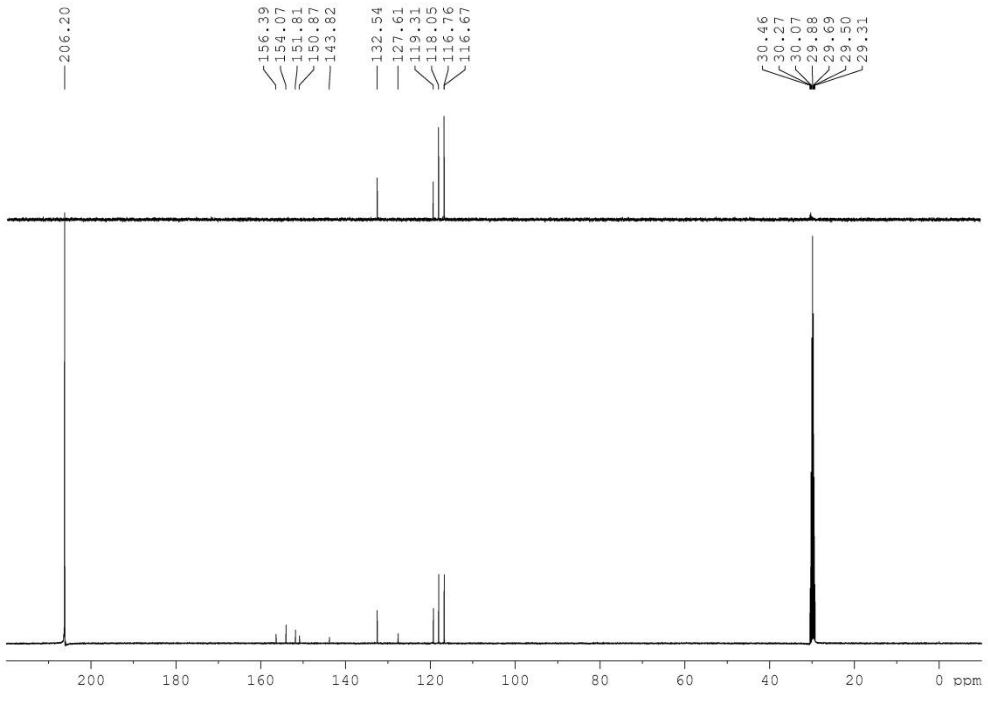

^{1}H NMR (CD_3COCD_3, 400 MHz), ^{13}C NMR (CD_3COCD_3, 100 MHz), and DEPT 135 spectra of **4i'**

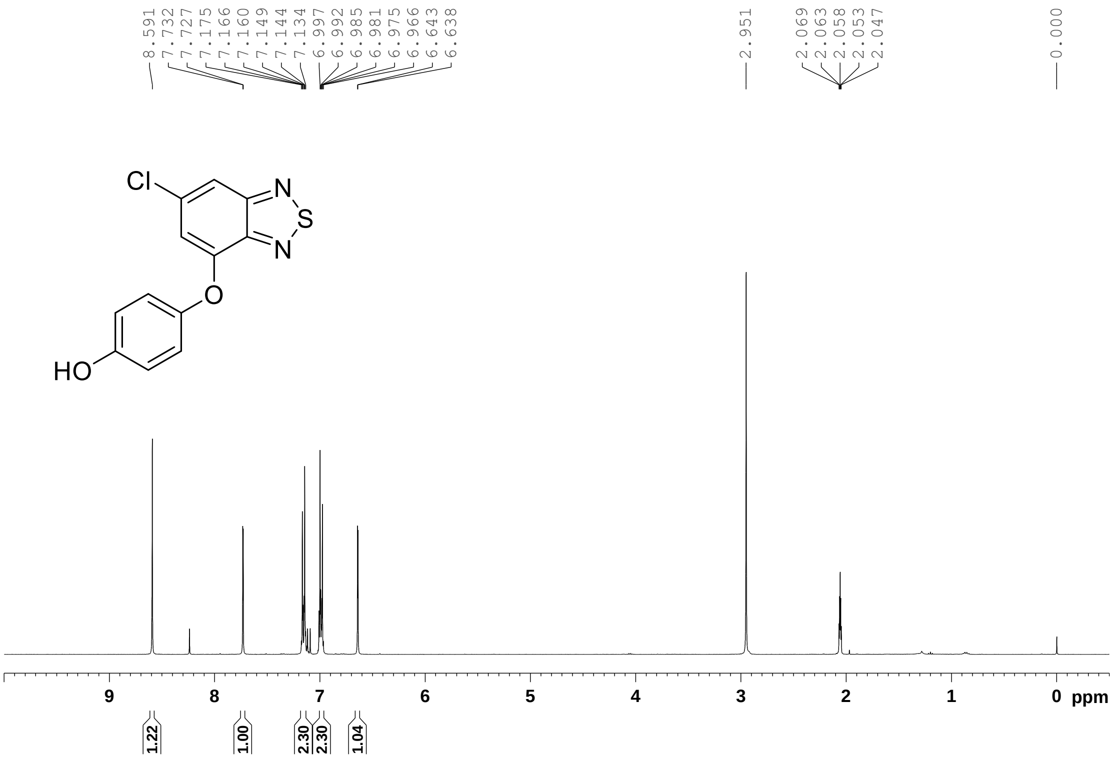

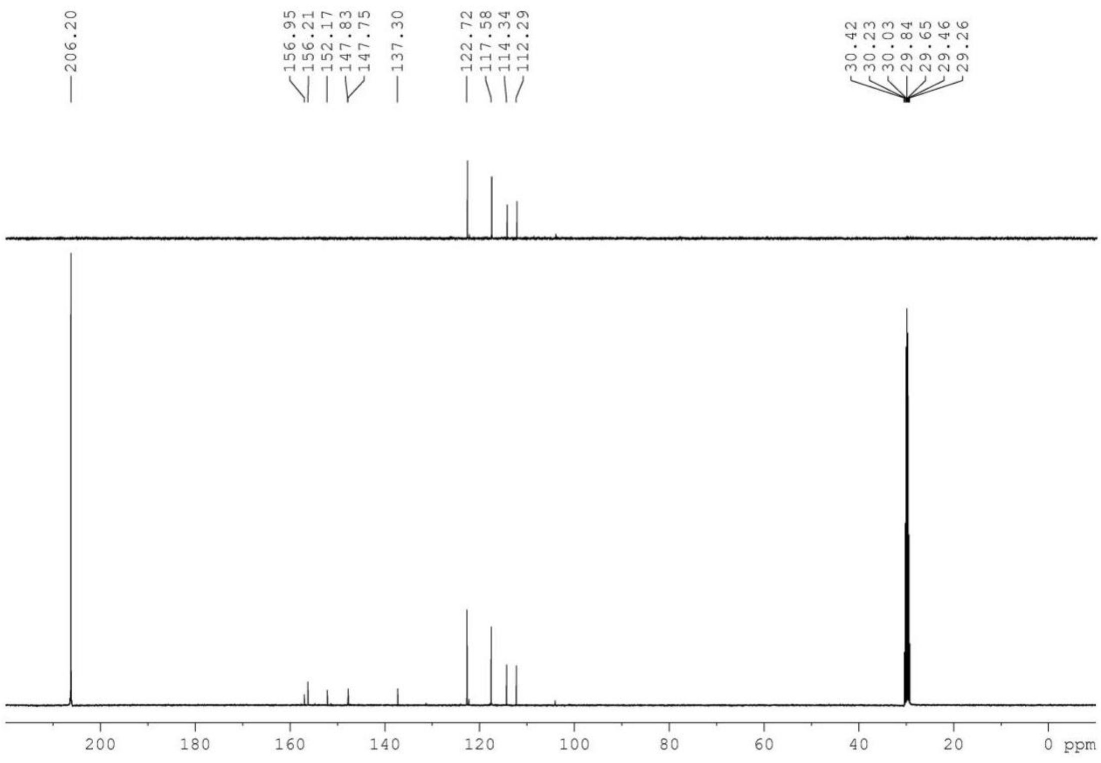

^{1}H NMR (CD_3COCD_3, 400 MHz), ^{13}C NMR (CD_3COCD_3, 100 MHz), and DEPT 135 spectra of **4j**

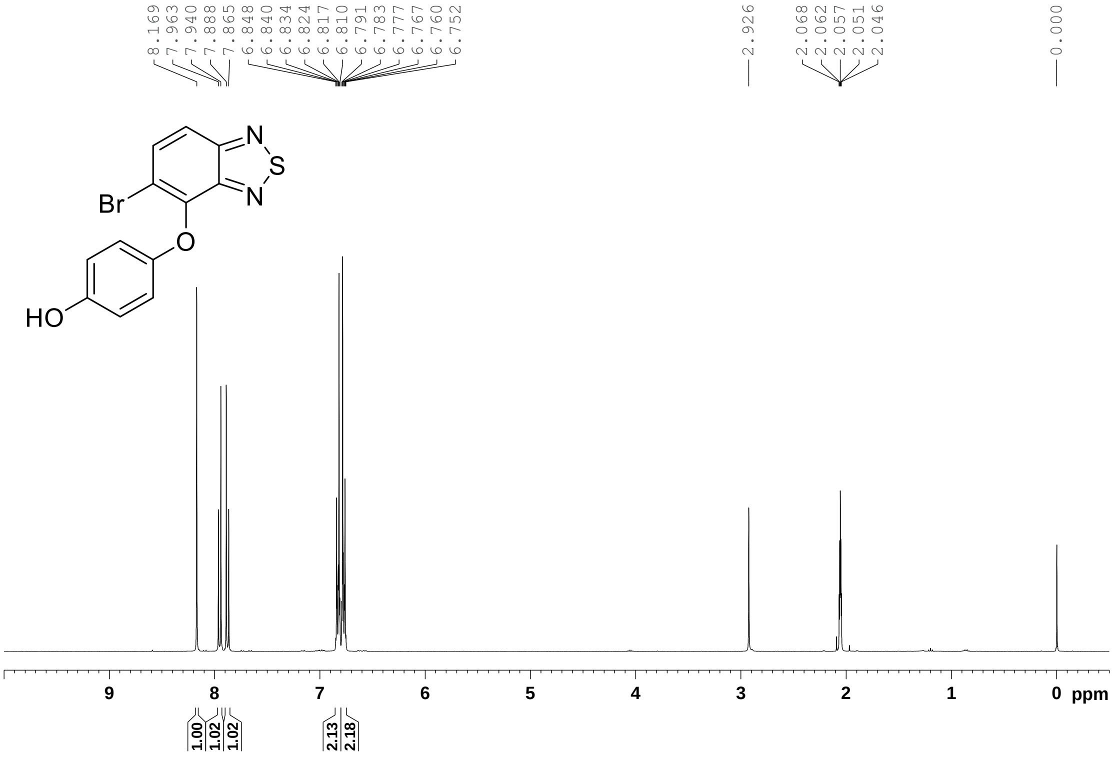

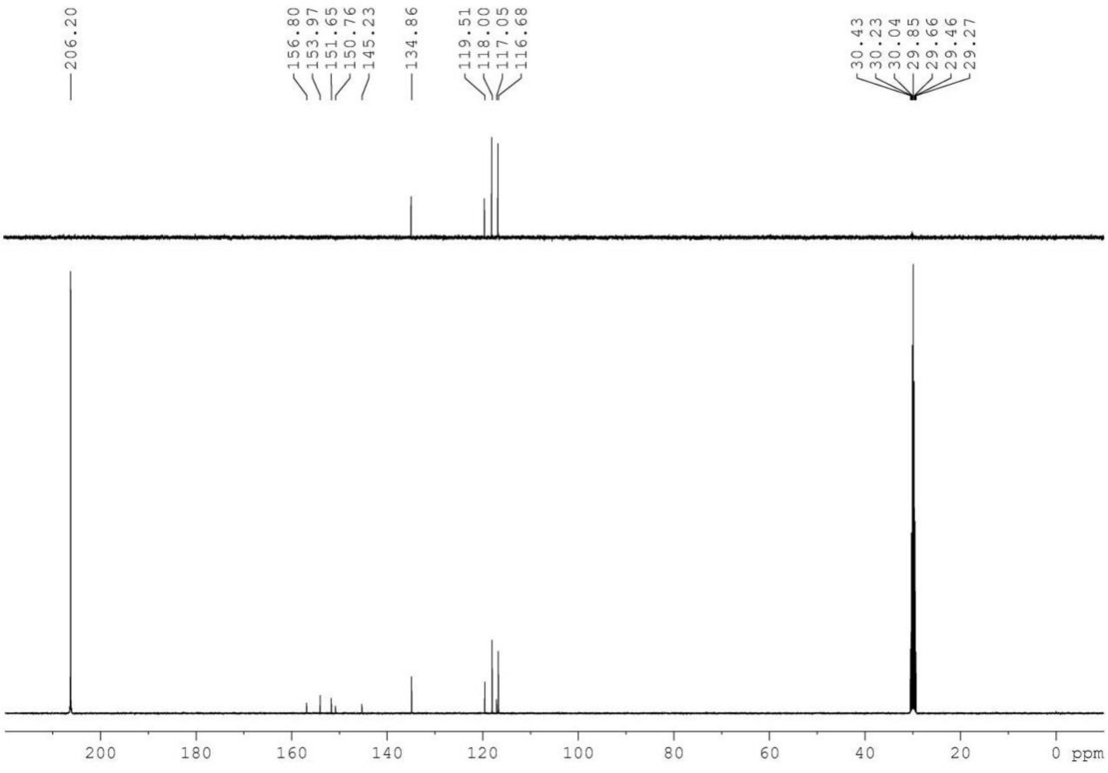

^{1}H NMR (CD_3COCD_3, 400 MHz), ^{13}C NMR (CD_3COCD_3, 100 MHz), and DEPT 135 spectra of **4j'**

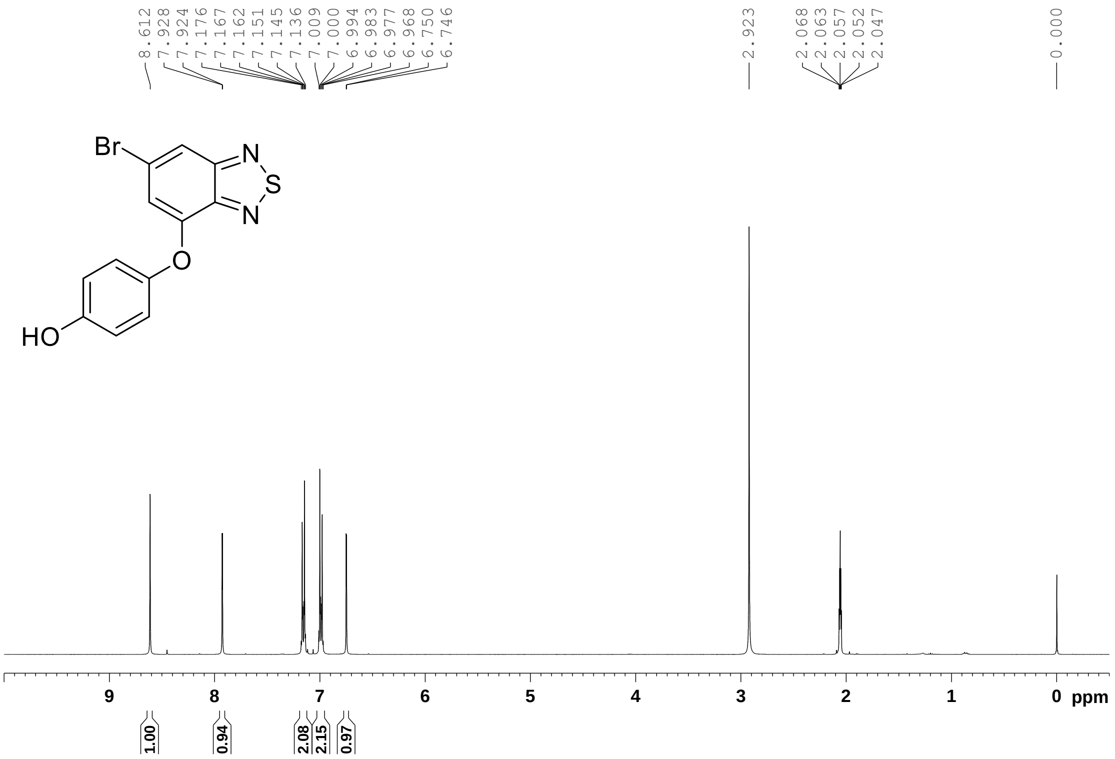

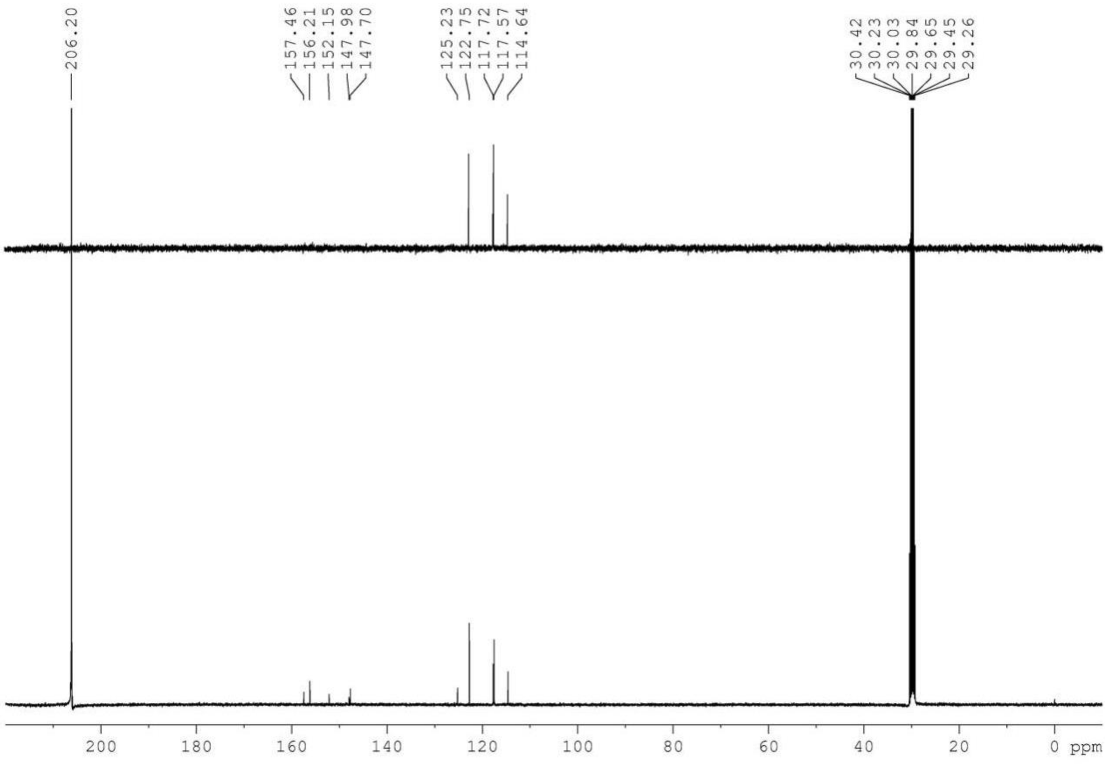

^{1}H NMR (CD_3COCD_3, 400 MHz), ^{13}C NMR (CD_3COCD_3, 100 MHz), and DEPT 135 spectra of **4k**

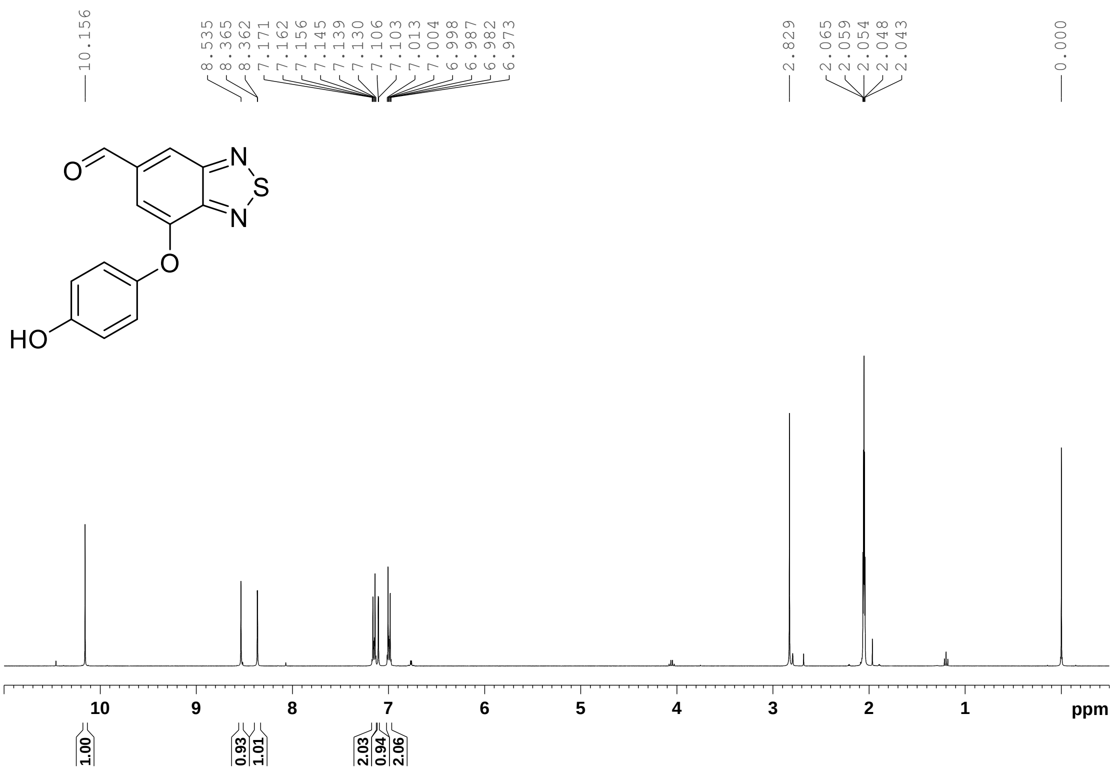

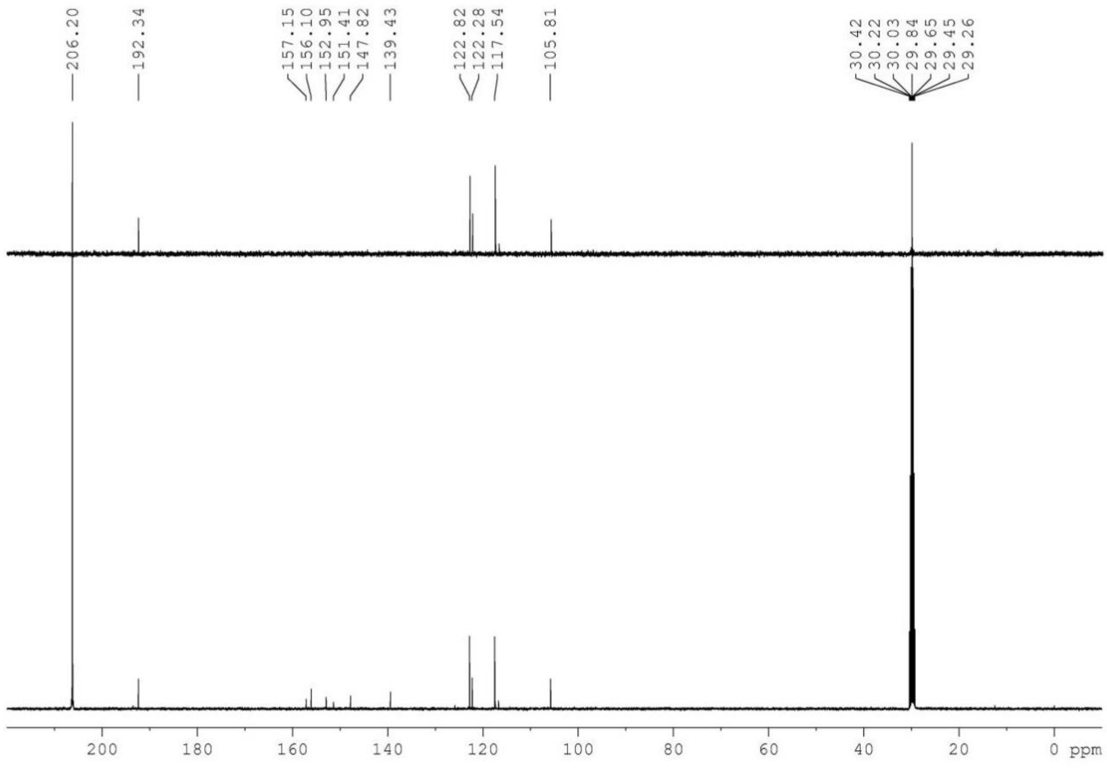

^{1}H NMR (CD_3COCD_3, 400 MHz), ^{13}C NMR (CD_3COCD_3, 100 MHz), and DEPT 135 spectra of **4l**

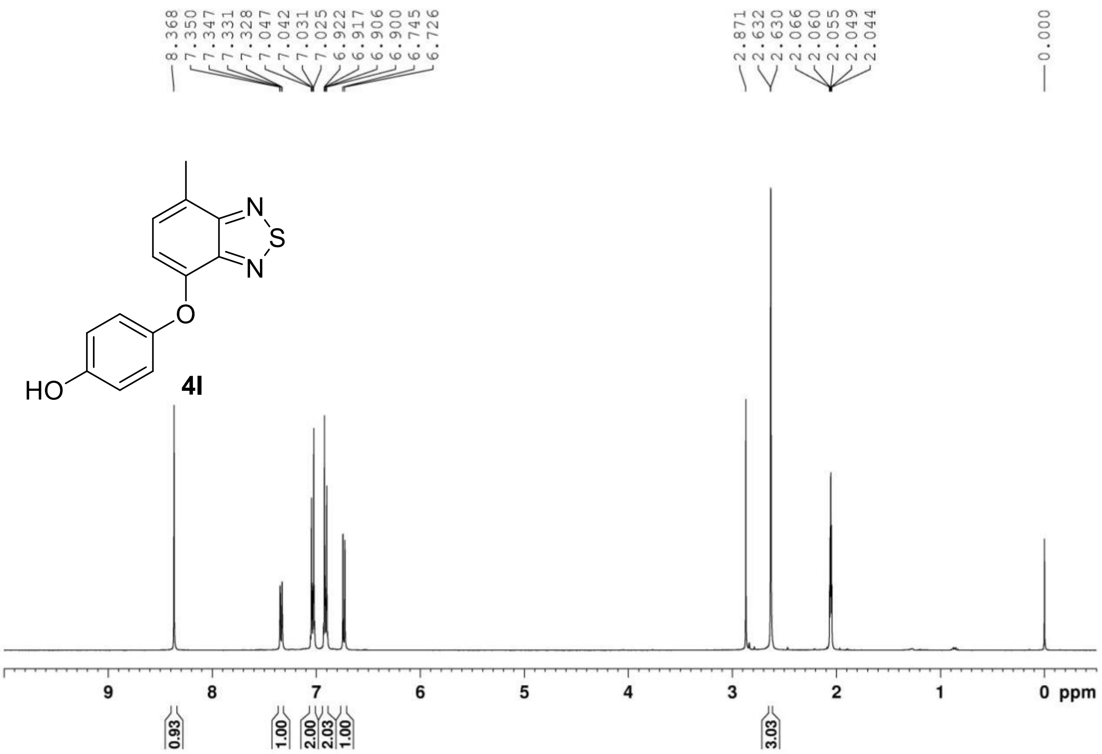

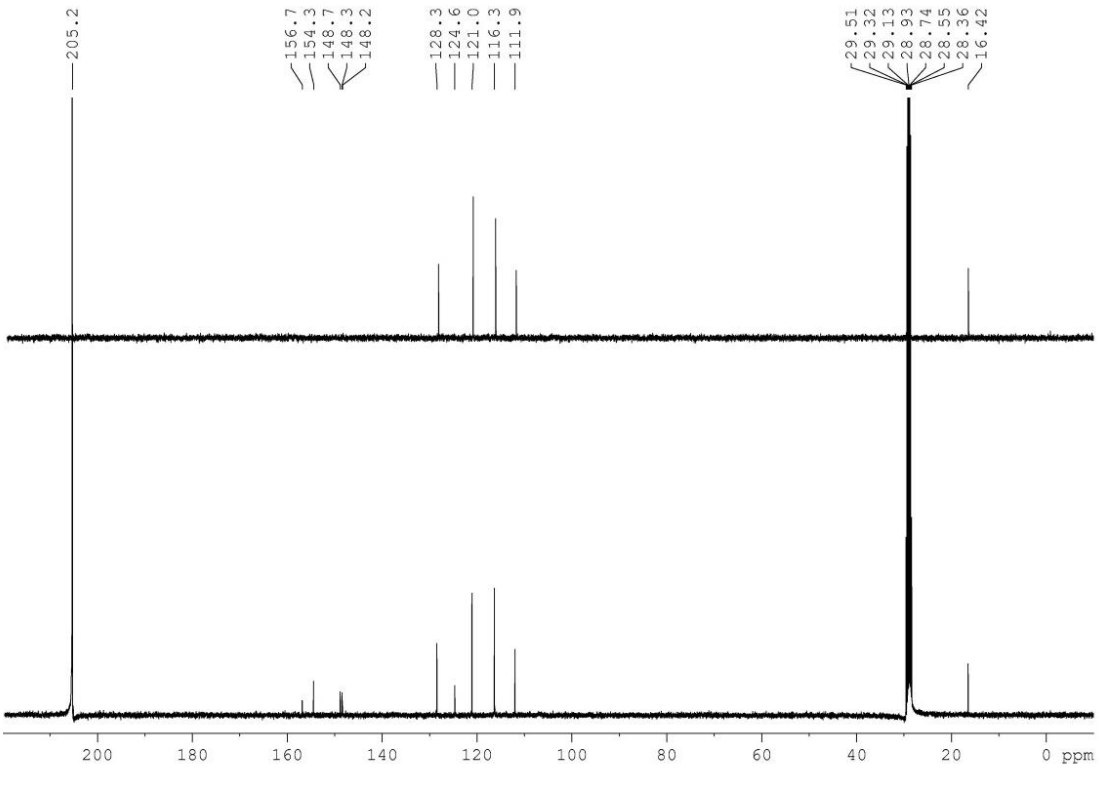

^1H NMR (CD_3COCD_3, 400 MHz), ^{13}C NMR (CD_3COCD_3, 100 MHz), and DEPT 135 spectra of **4m**

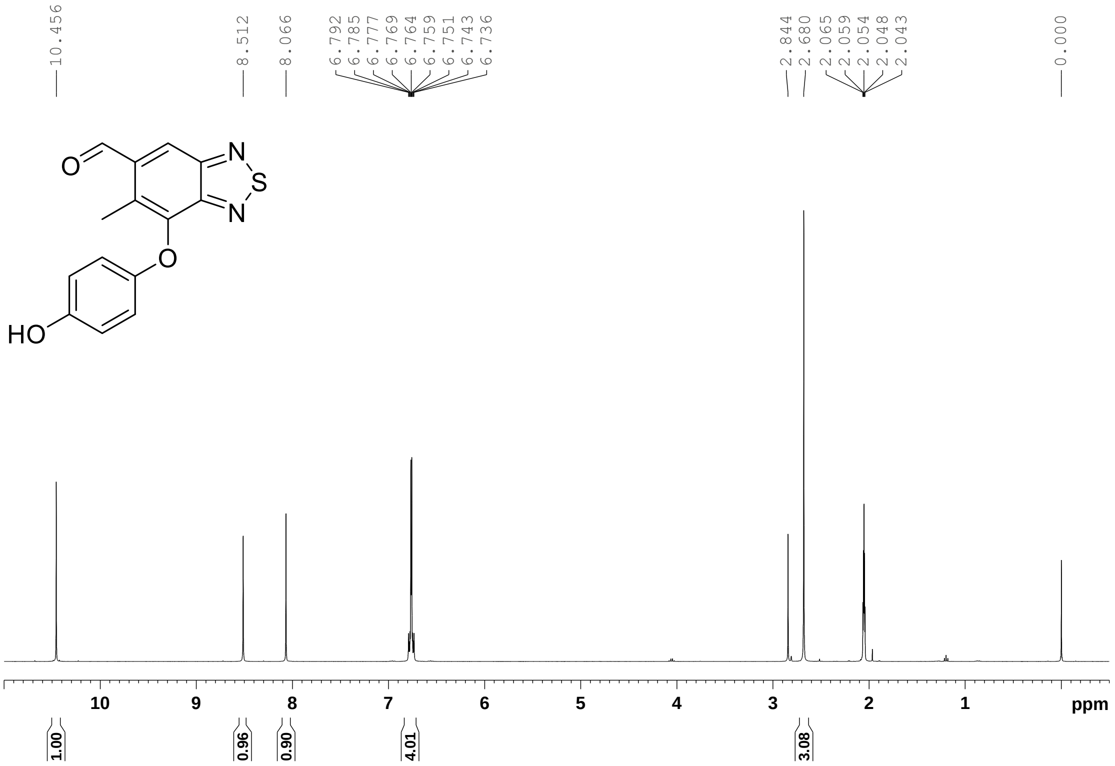

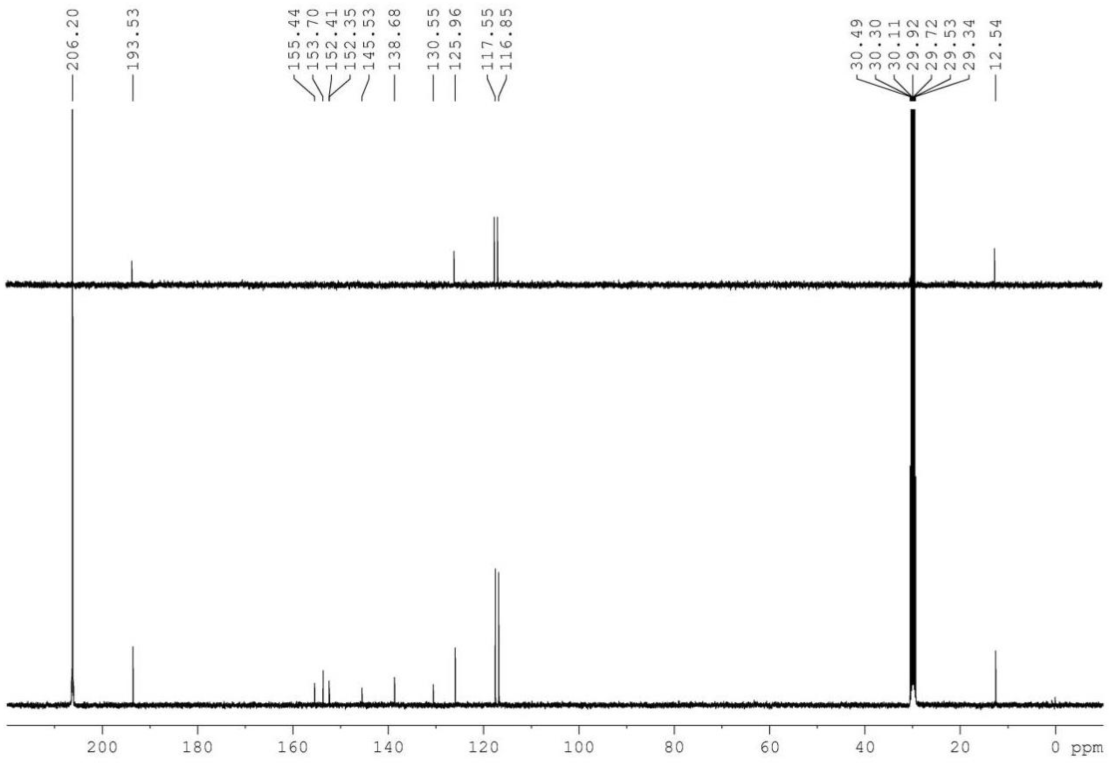

^{1}H NMR (CD_3COCD_3, 400 MHz), ^{13}C NMR (CD_3COCD_3, 100 MHz), and DEPT 135 spectra of **4n**

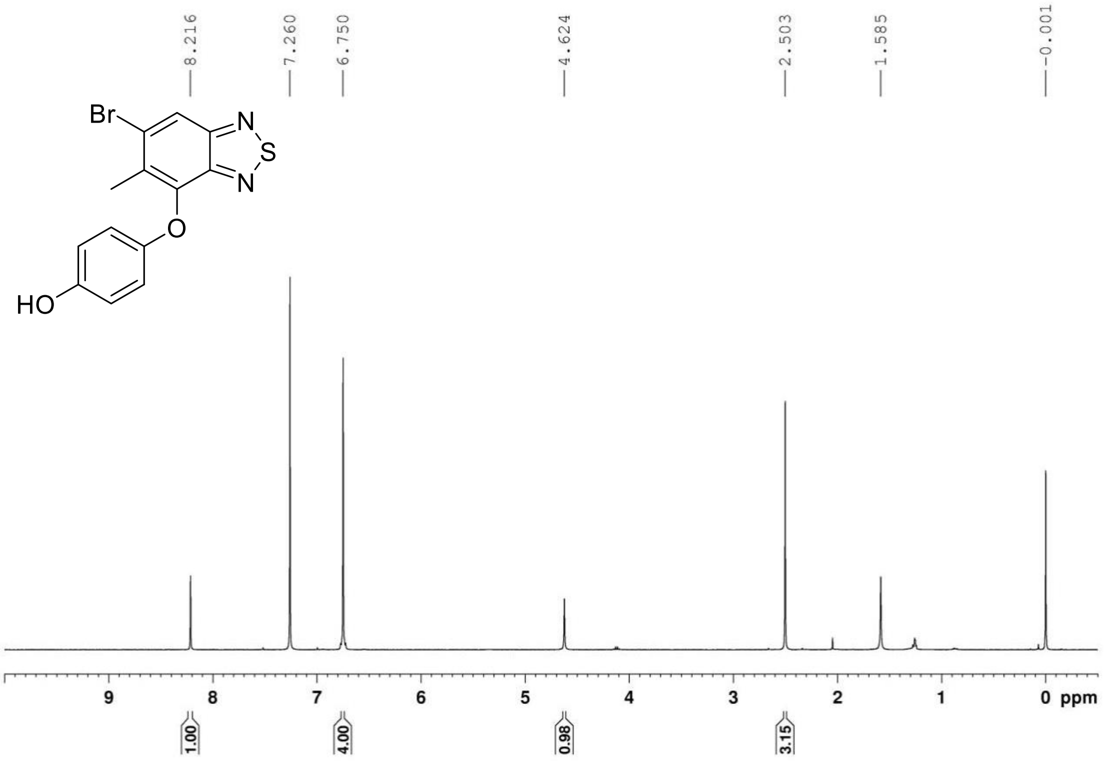

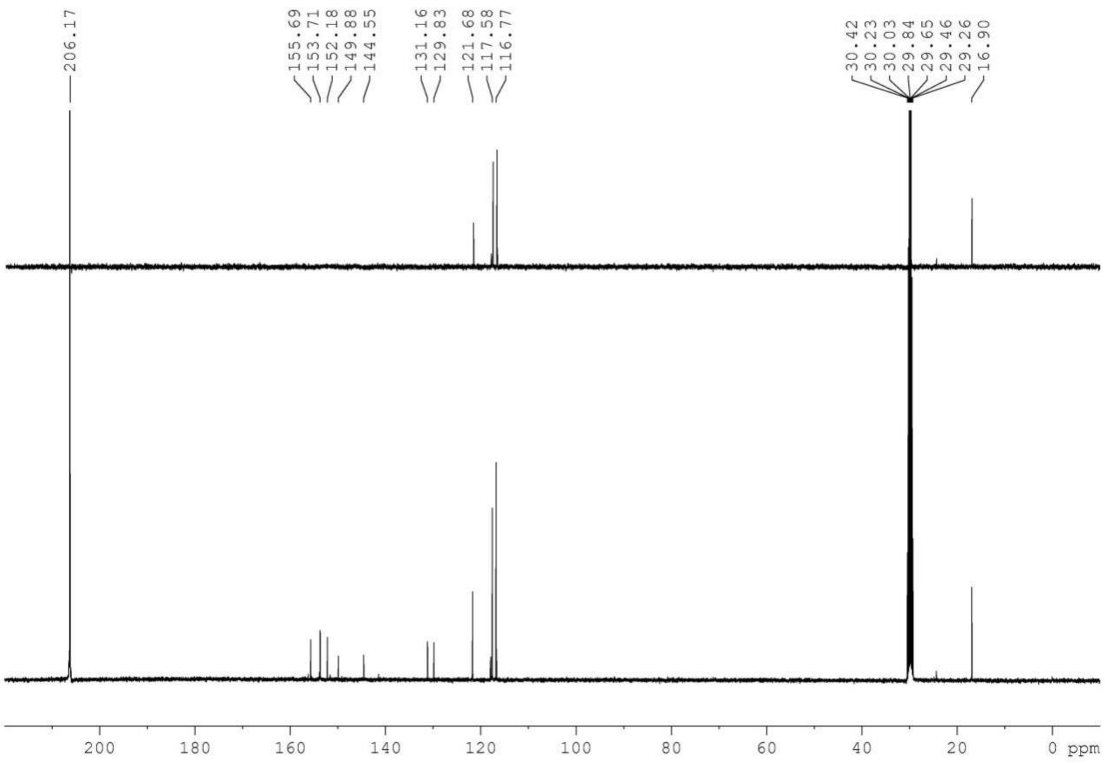

^{1}H NMR (CD_3COCD_3, 400 MHz), ^{13}C NMR (CD_3COCD_3, 100 MHz), and DEPT 135 spectra of **4n'**

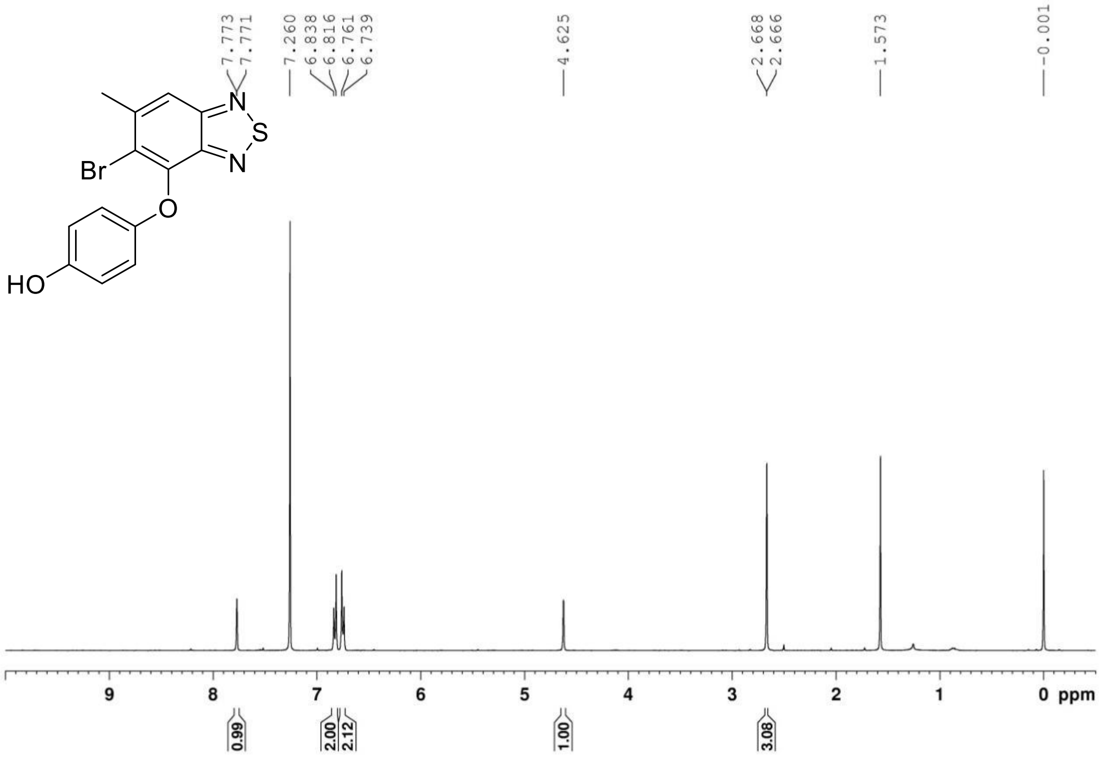

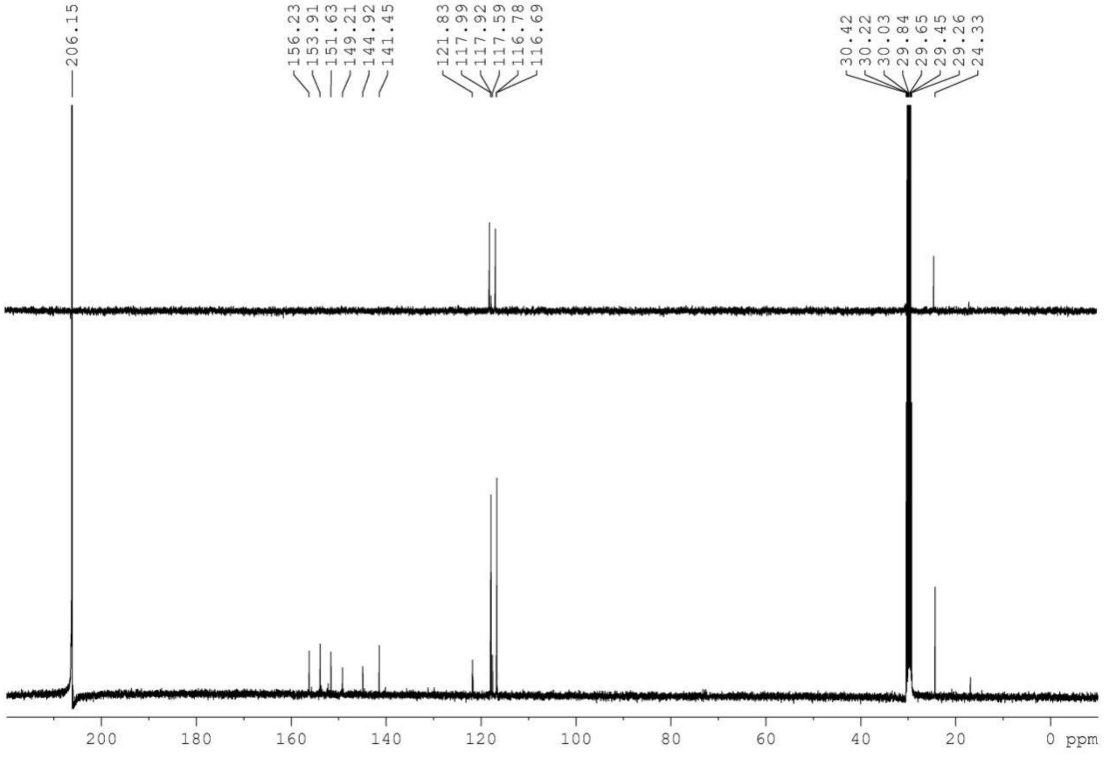

^{1}H NMR ($CDCl_3$, 400 MHz), ^{13}C NMR ($CDCl_3$, 100 MHz), and DEPT 135 spectra of **4o**

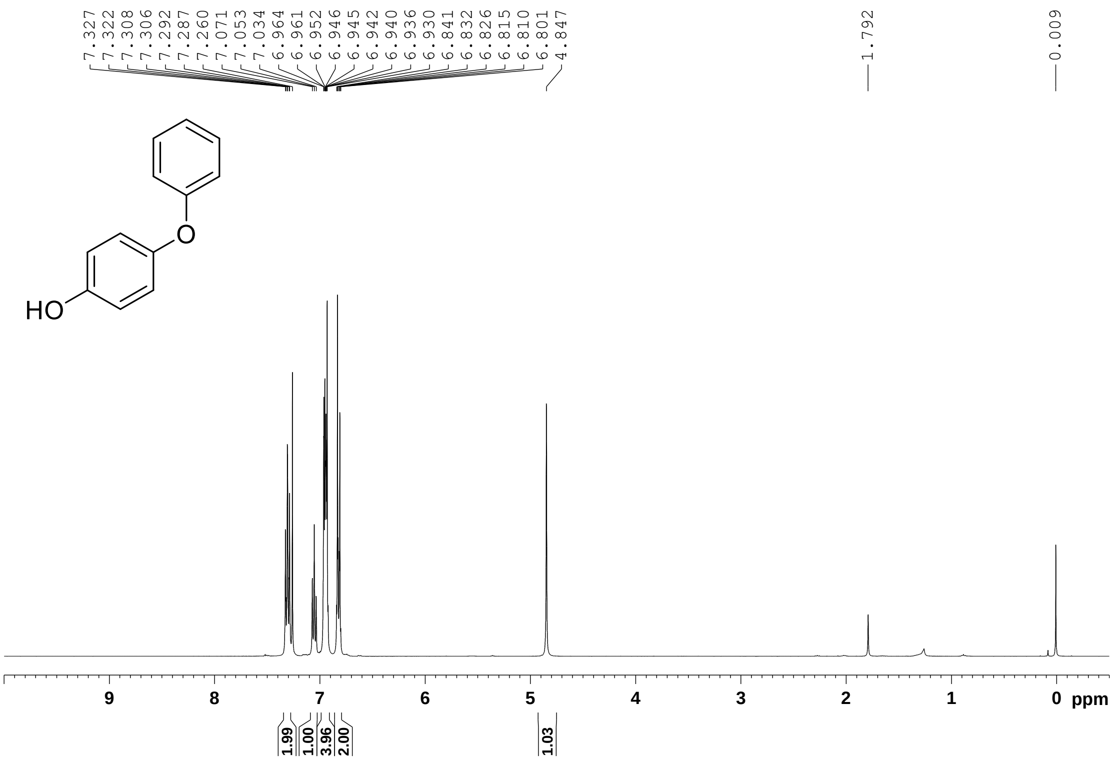
7.327
7.322
7.308
7.306
7.292
7.287
7.260
7.071
7.053
7.034
6.964
6.961
6.952
6.946
6.945
6.942
6.940
6.936
6.930
6.841
6.832
6.826
6.815
6.810
6.801
4.847
1.792
0.009
HO
O
9
8
7
6
5
4
3
2
1
0 ppm
1.99
1.00
3.96
2.00
1.03

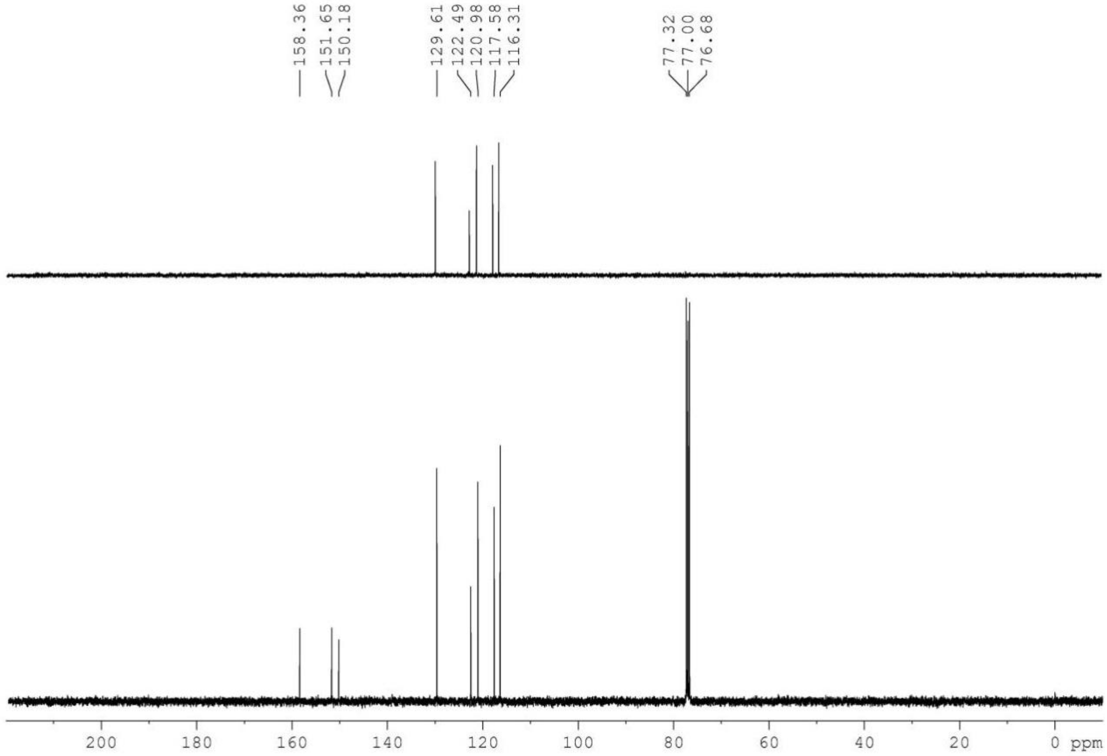
158.36
151.65
150.18
129.61
122.49
120.98
117.58
116.31
77.32
77.00
76.68
200
180
160
140
120
100
80
60
40
20
0 ppm

^{1}H NMR ($CDCl_3$, 400 MHz), ^{13}C NMR ($CDCl_3$, 100 MHz), and DEPT 135 spectra of **4p**

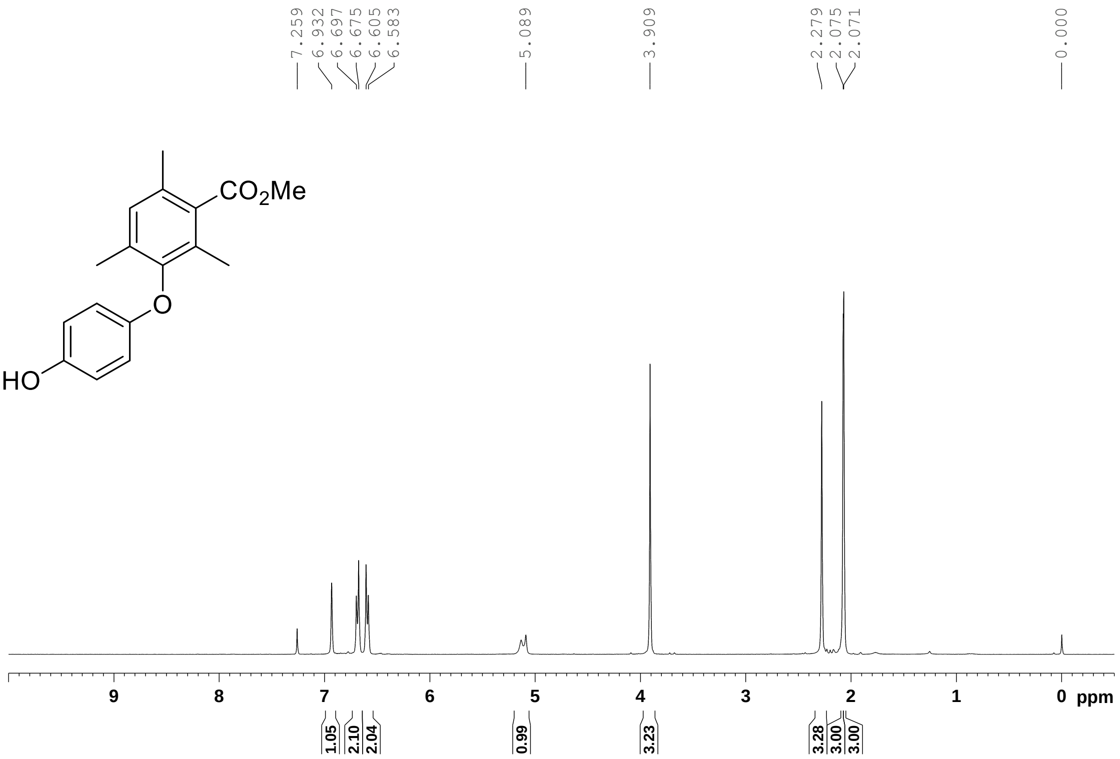

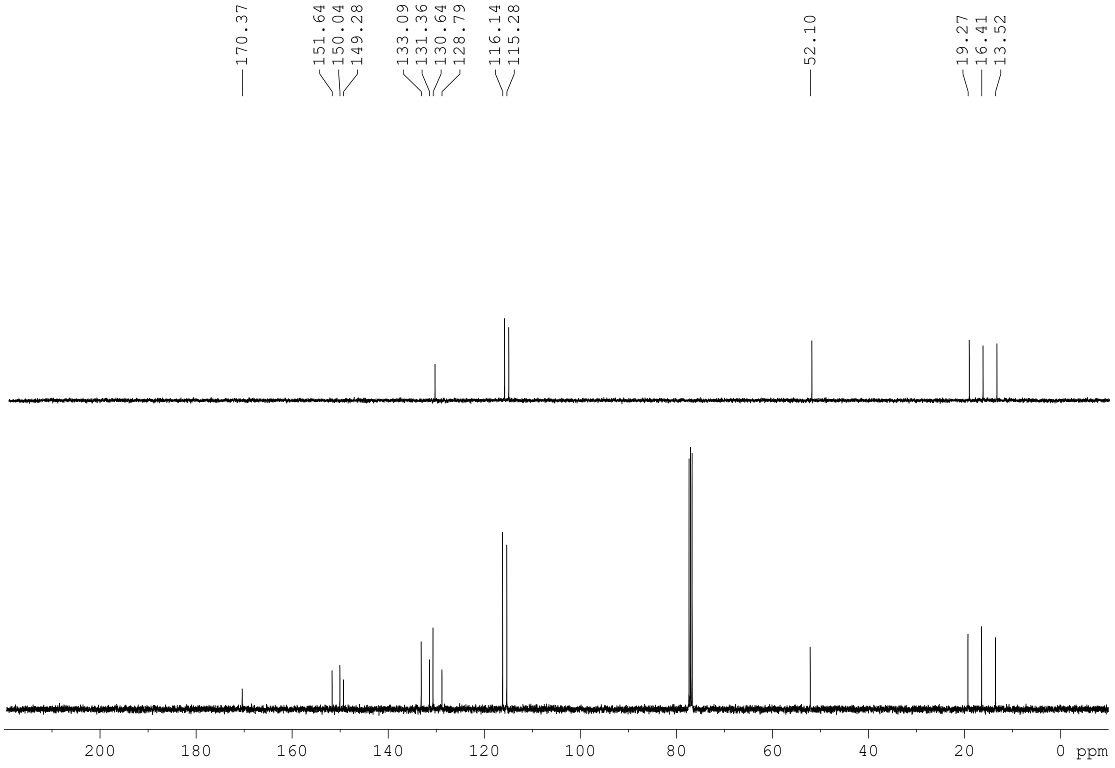

^1H NMR ($CDCl_3$, 400 MHz), ^{13}C NMR ($CDCl_3$, 100 MHz), and DEPT 135 spectra of **4q**

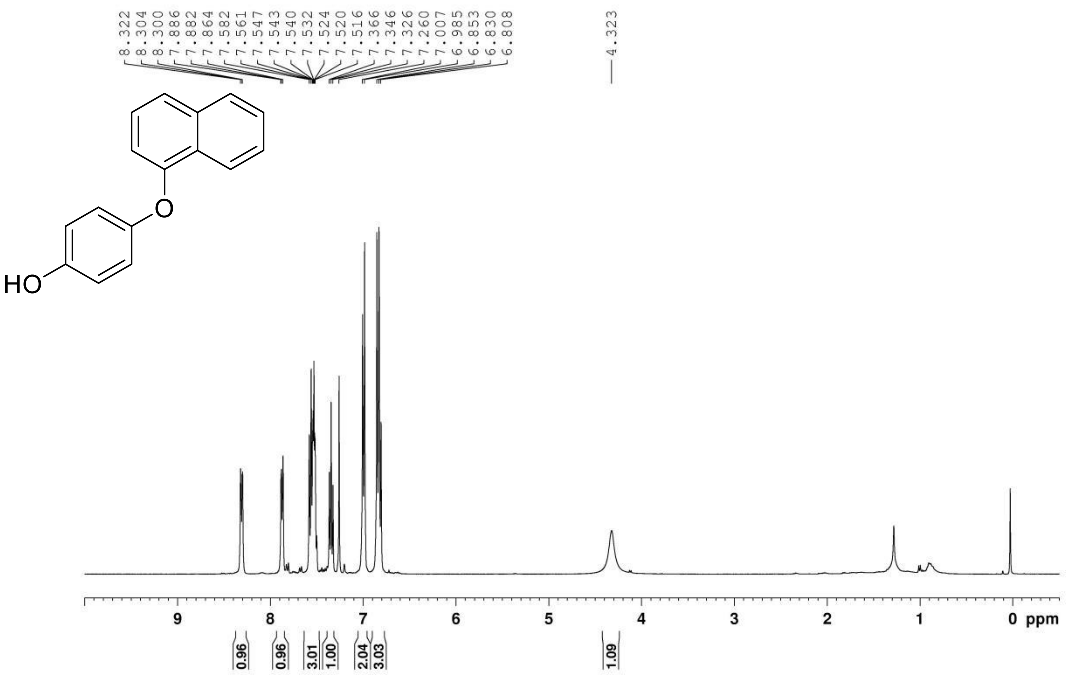

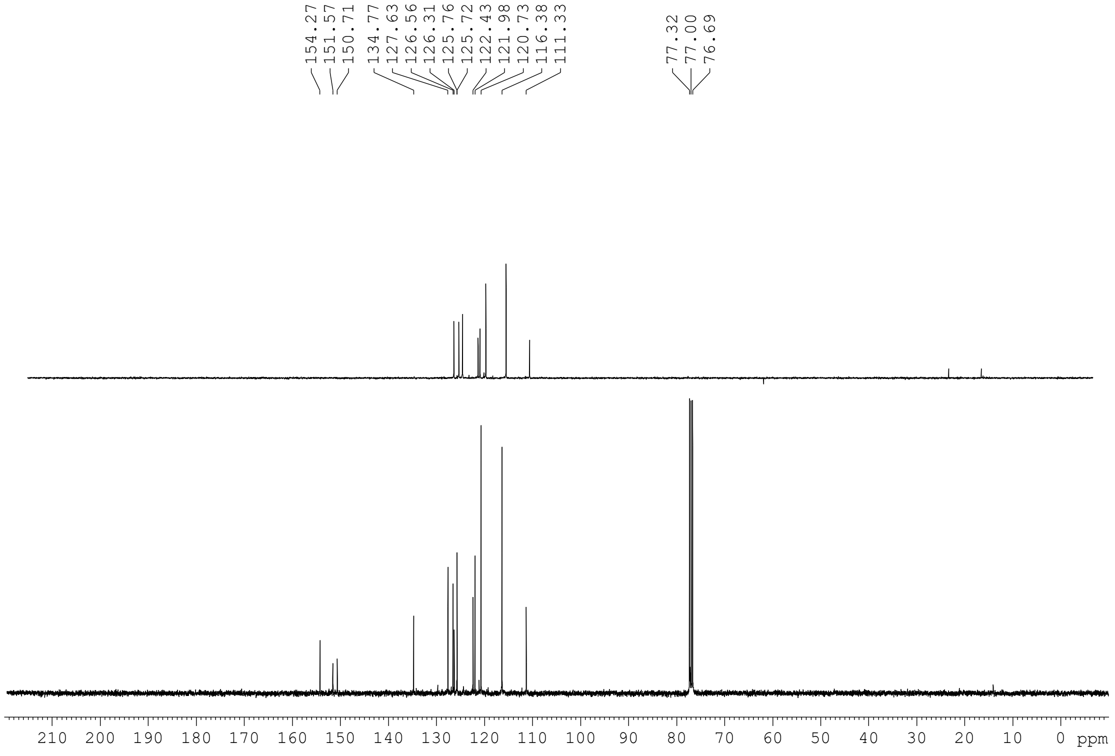

^{1}H NMR ($CDCl_3$, 400 MHz), ^{13}C NMR ($CDCl_3$, 100 MHz), and DEPT 135 spectra of **4r**

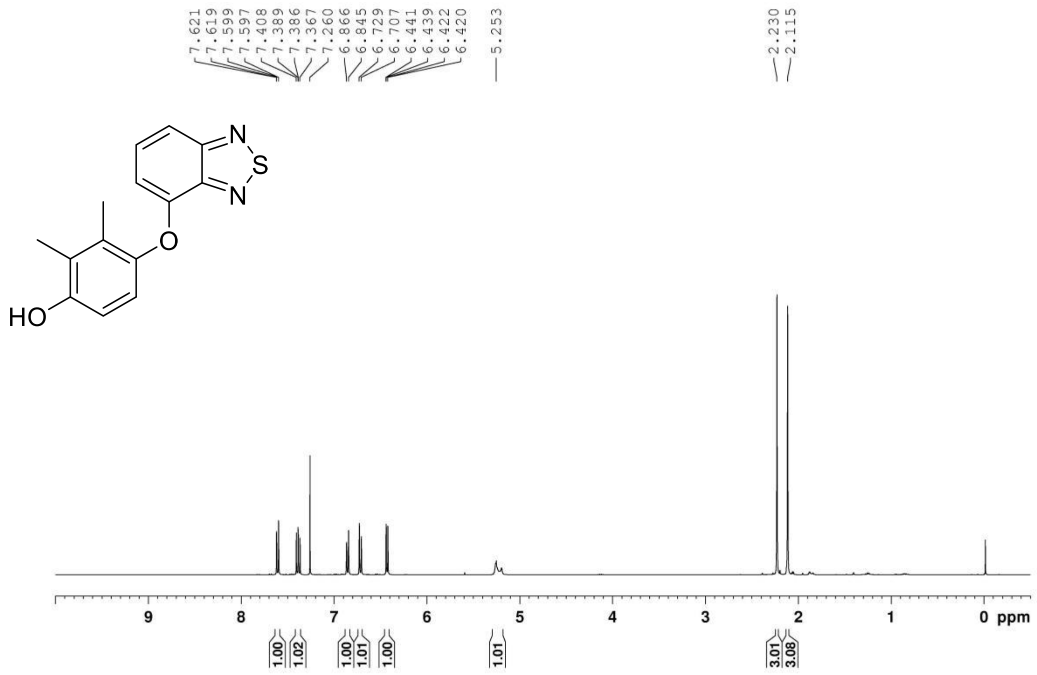

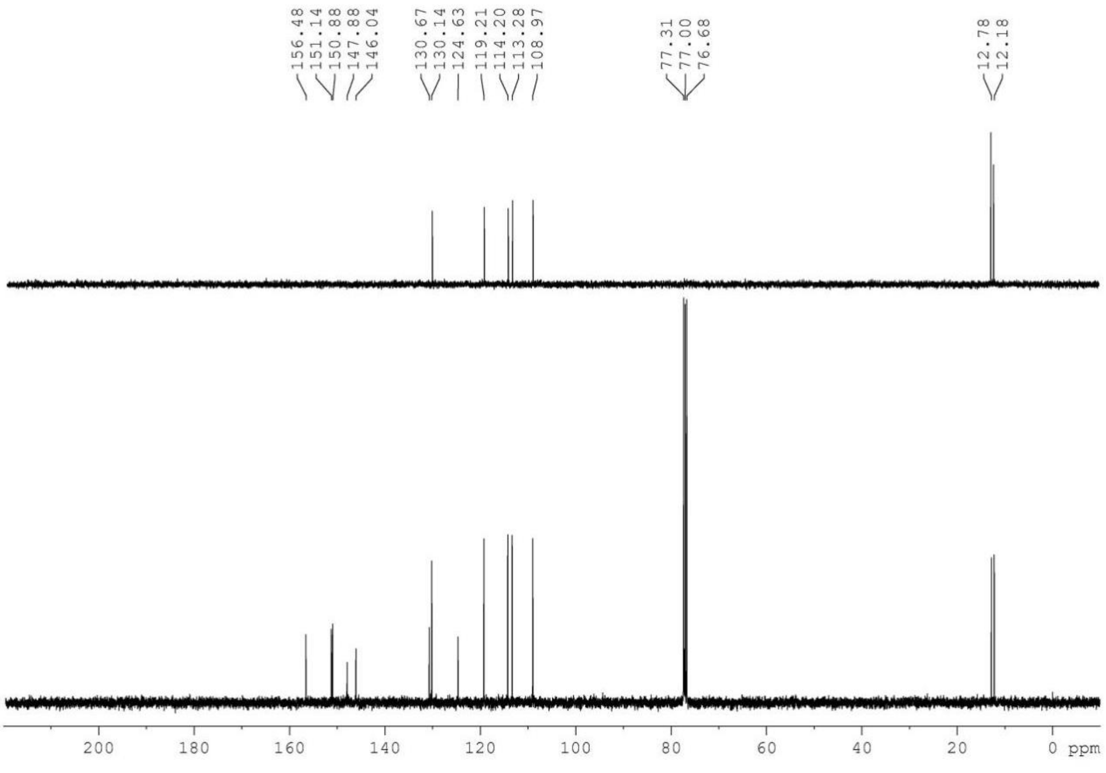

^{1}H NMR ($CDCl_3$, 400 MHz), ^{13}C NMR ($CDCl_3$, 100 MHz), and DEPT 135 spectra of **4s**

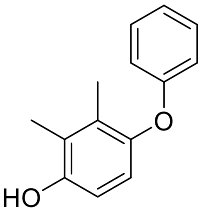

4s

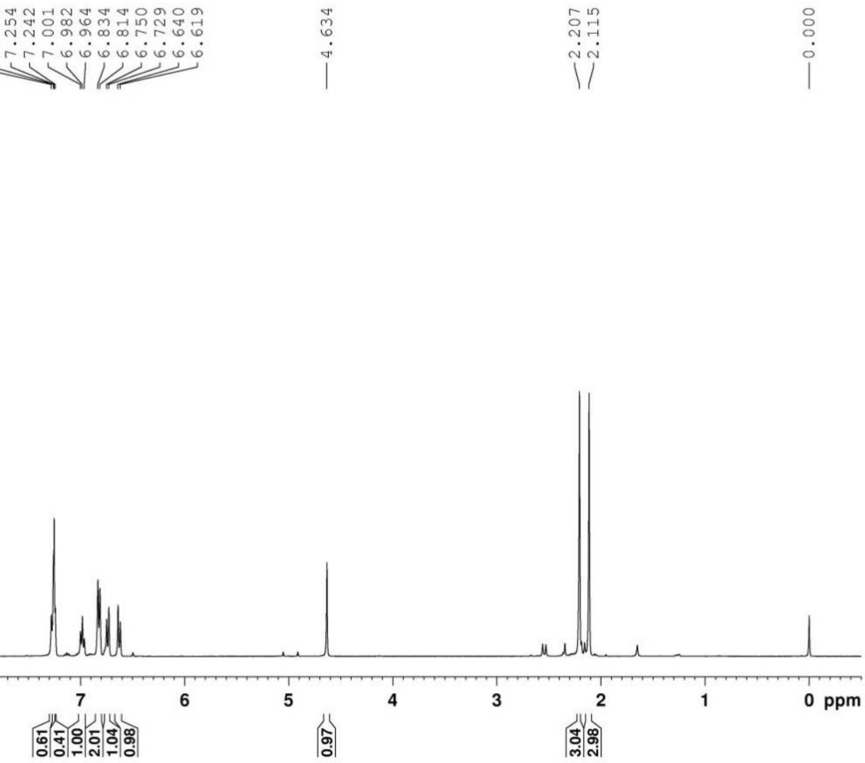

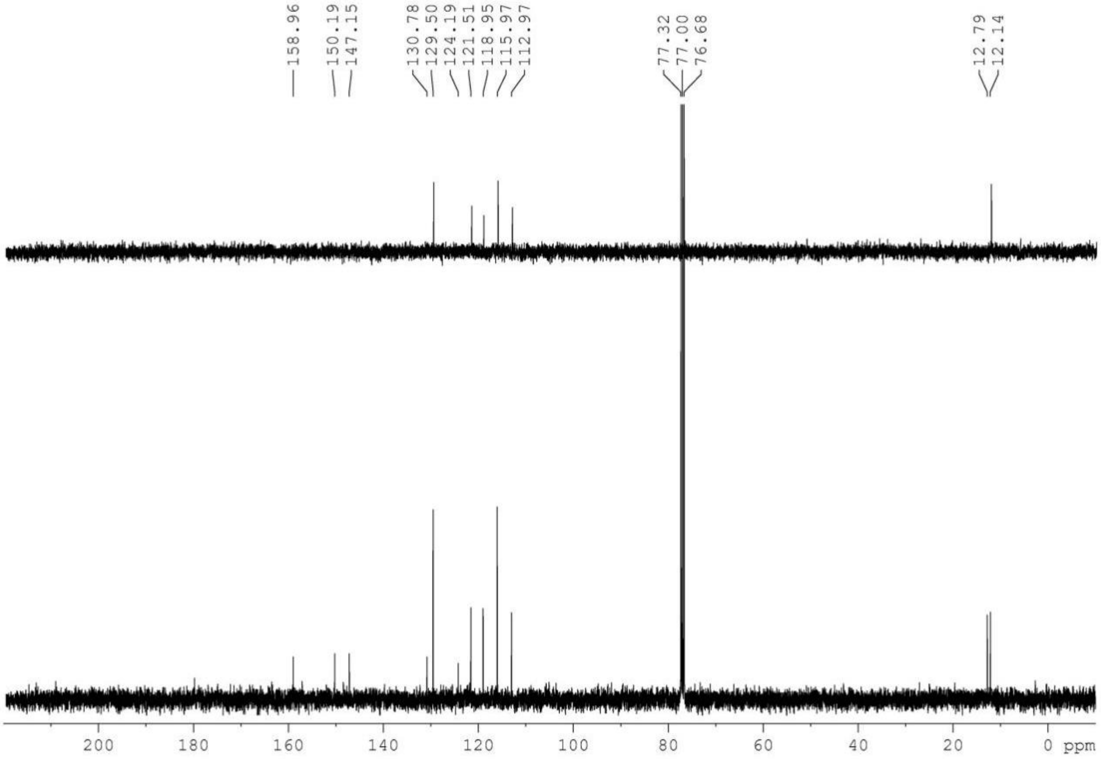

^{1}H NMR ($CDCl_3$, 400 MHz), ^{13}C NMR ($CDCl_3$, 100 MHz), and DEPT 135 spectra of **4t**

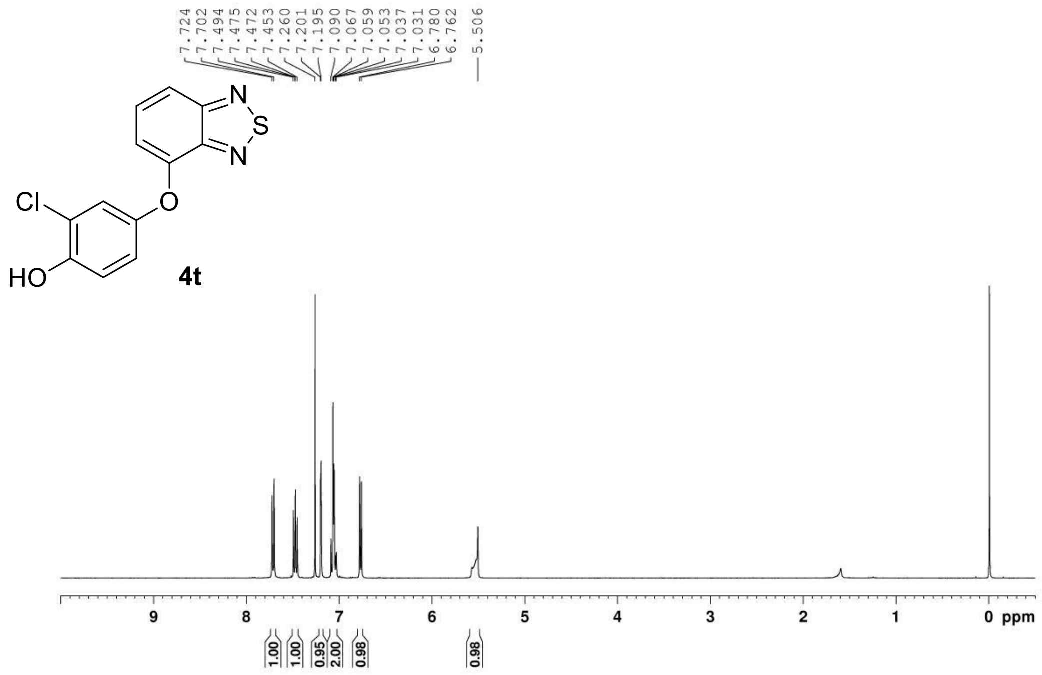

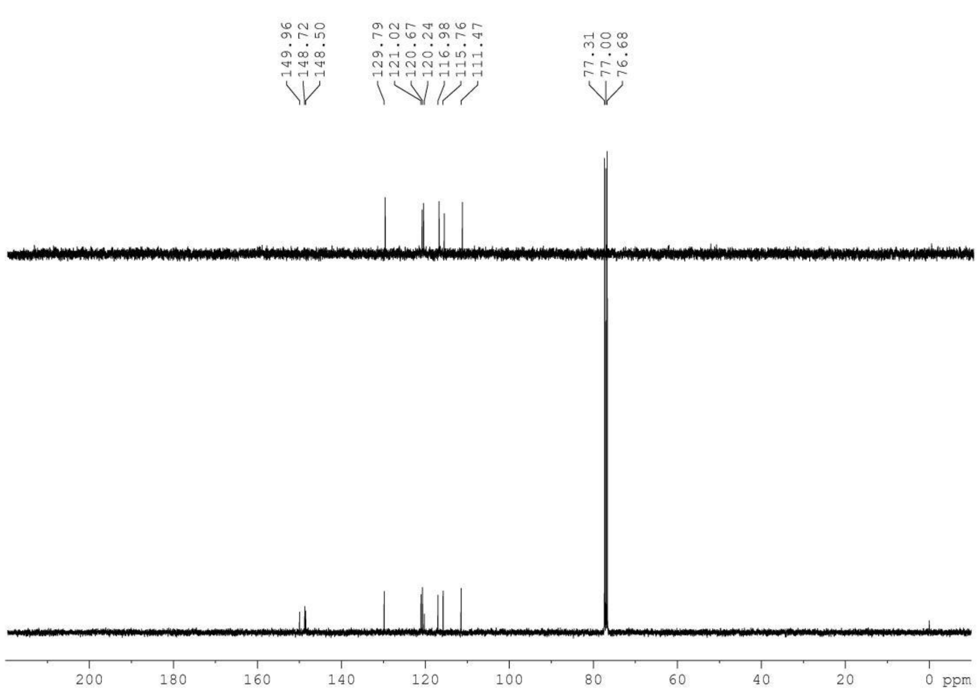

^{1}H NMR ($CDCl_3$, 400 MHz), ^{13}C NMR ($CDCl_3$, 100 MHz), and DEPT 135 spectra of **4u**

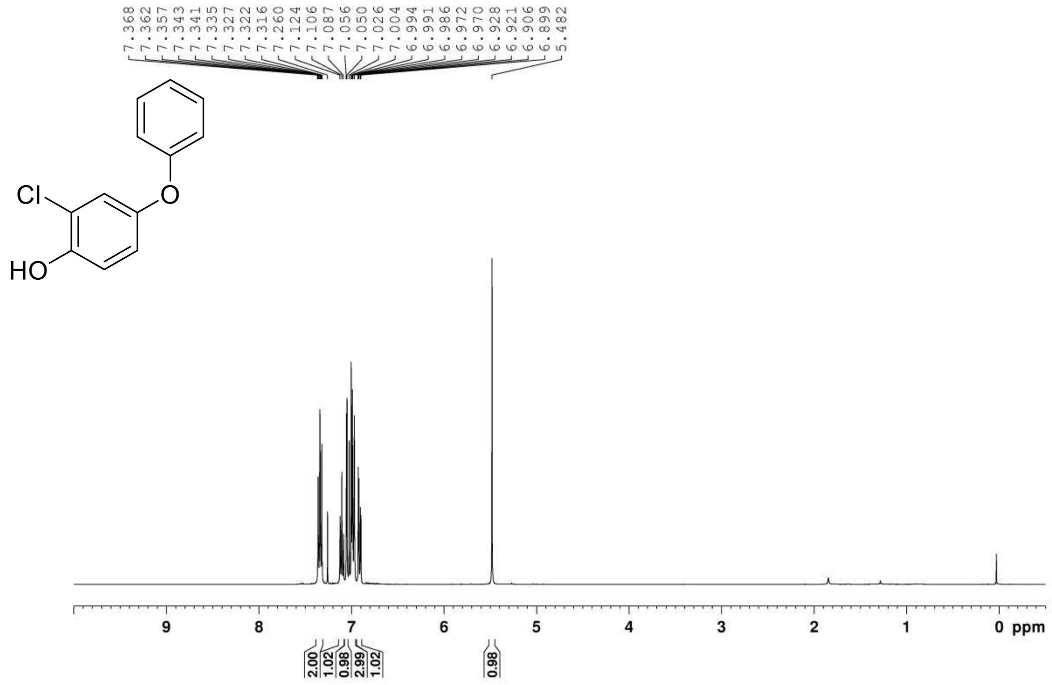

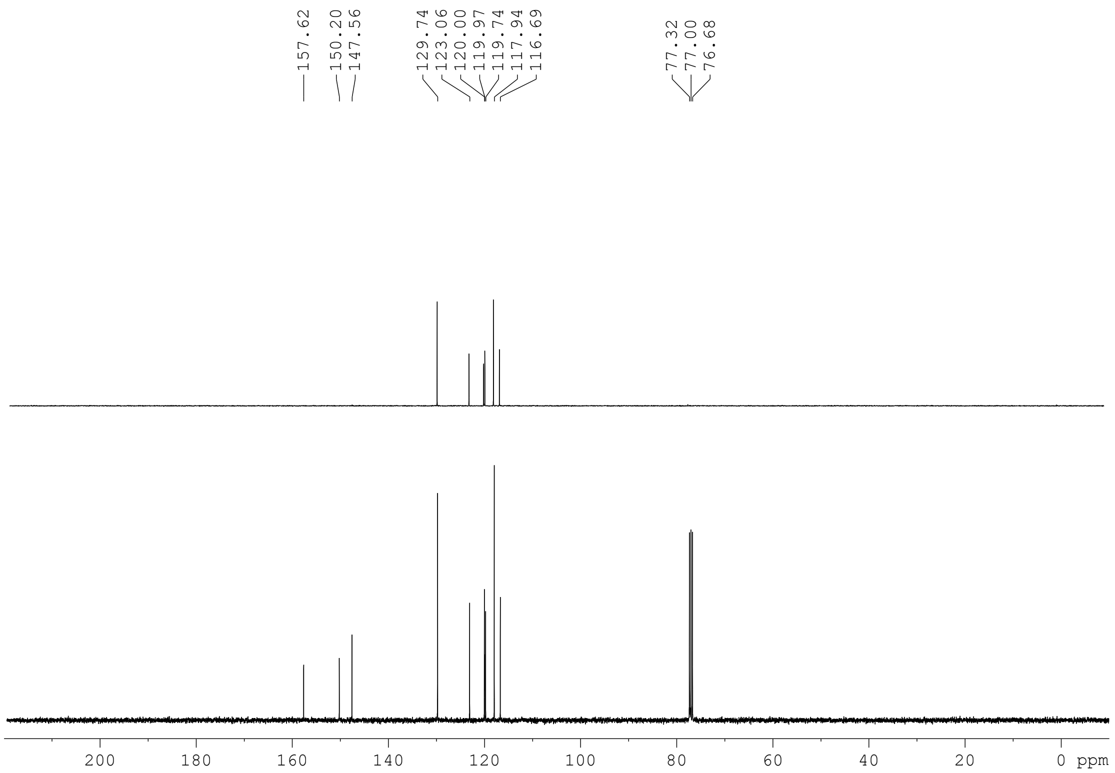

^{1}H NMR ($CDCl_3$, 400 MHz) and ^{13}C NMR ($CDCl_3$, 100 MHz) spectra of **5**

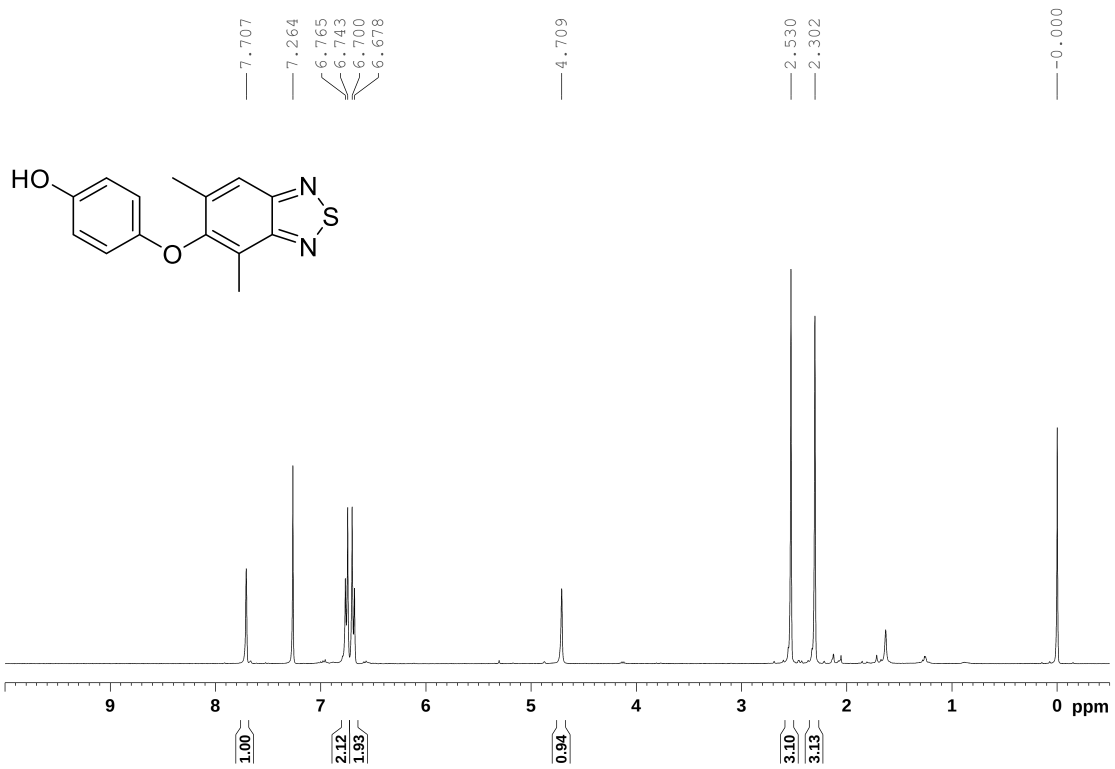

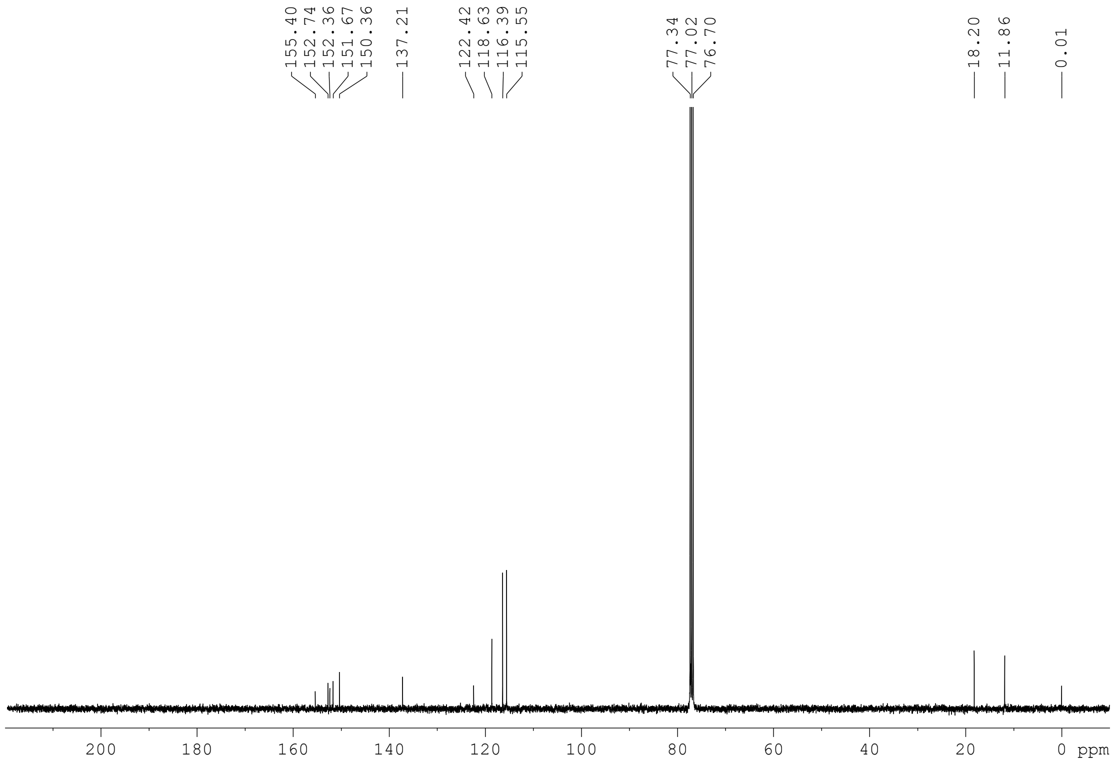

5. Mechanistic investigation

Based on the literature,[1] Paterno-Buchi reaction can proceed either via a) formation of biradical intermediate, or b) formation of radical ion pair intermediate followed by photoinduced electron transfer (PET).

The UV-Vis absorption and fluorescence spectra of **1a** and **2a** were investigated. As shown in Fig. S1, only **2a** had an absorption peak at 450 nm, and it was not affected when **1a** or $Cu(OTf)_2$ was added, which indicated that **2a** was exited under the two optimized conditions firstly without the effect by **1a** or $Cu(OTf)_2$. The fluorescence of **2a** was not quenched by the increase of the proportion of **1a** (Fig. S2a) and the fluorescence lifetime of **2a** at 505 nm was maintained at the nanosecond level (Fig. S2c), which indicated that **1a** or $Cu(OTf)_2$ did not reduce singlet excited state generation of **2a**. The fluorescence of **2a**+**1a** (1:3) was not affected by $Cu(OTf)_2$ (Fig. S2b). So, $Cu(OTf)_2$ just acted as a Lewis acid in the reaction of the synthesis of diaryl ethers.

The result of the energy transfer calculations from the Gibbs energy of PET equation of the reaction of **1a** and **2a** showed $\Delta G > 0$, which also forbided the possibility of PET mechanism (Scheme 5). So, we propose that the reaction proceeds via the formation of a biradical intermediate.

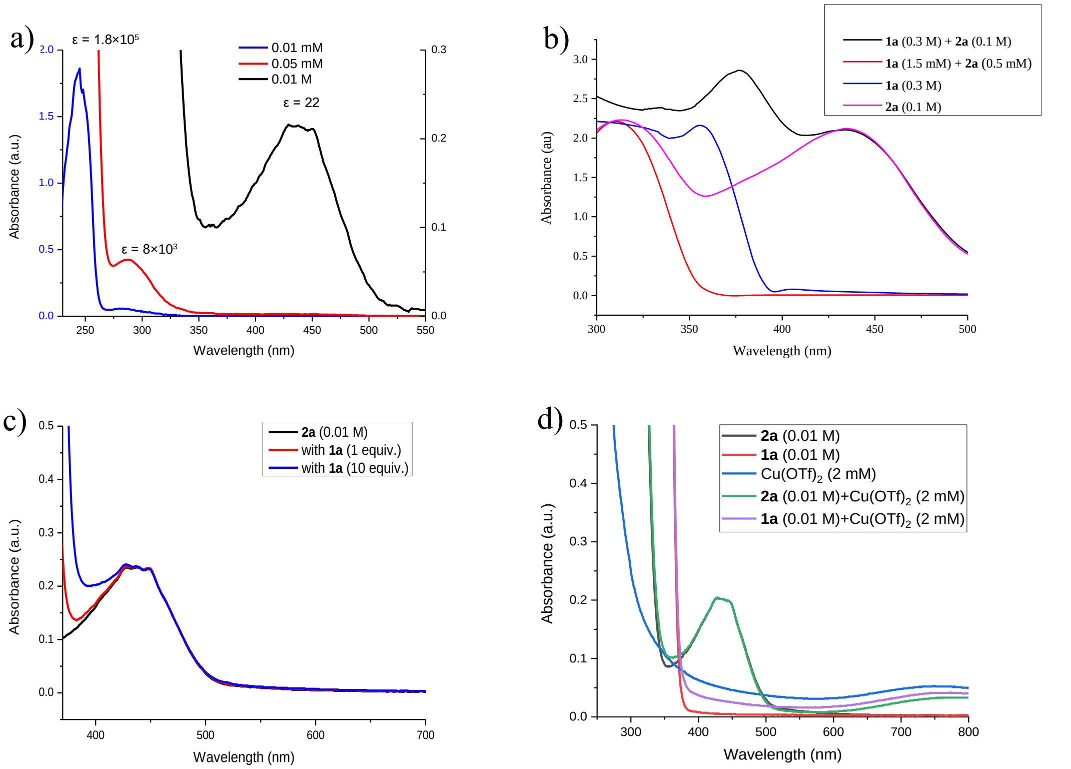

Figure S1. UV-vis absorption spectra in DCM of a) **2a** with different concentration; b) **1a** and **2a** in reaction concentration in DCM; c) **2a** with different equivalent weight of **1a** in DCM; d) **1a**, **2a** and $Cu(OTf)_2$ in CH_3CN.

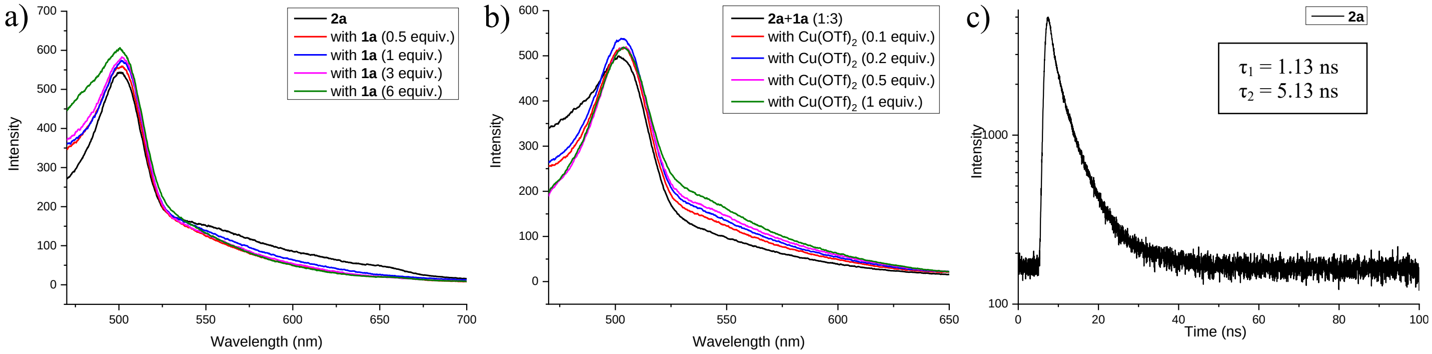

Figure S2. The fluorescence spectra of a) **2a** with different equivalent weight of **1a**; b) **2a**+**1a** (1:3) with different equivalent weight of **1a** $Cu(OTf)_2$ in CH_3CN excited at 450 nm; c) transient PL spectra of **2a** in CH_3CN. The concentration of **2a** was (5 x 10^{-4} M).

Reaction with triplet quencher DABCO.

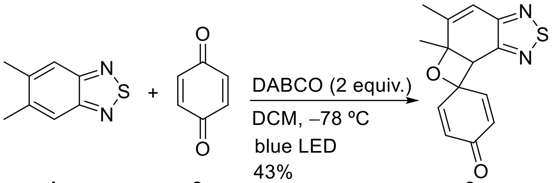

1a **2a** **3a**

To a quartz tube containing a stirring bar was added **1a** (148 mg, 0.9 mmol), quinone **2** (33 mg, 0.3 mmol), DABCO (68 mg, 0.6 mmol), and dichloromethane (3 mL). The reaction tube was degased with argon and stirred for 48 h at –78 °C under blue LED (λ = 450 nm, 5 W) irradiation. The reaction system was raised to room temperature, and transferred into a single-necked flask. The reaction mixture was concentrated *in vacuo* and separated by column chromatography (PE/EtOAc = 5:1) to obtain **3** (36 mg, 43%). It confirmed that no quenching of **2a** took place in the excited state.

Reaction under oxygen atmosphere.

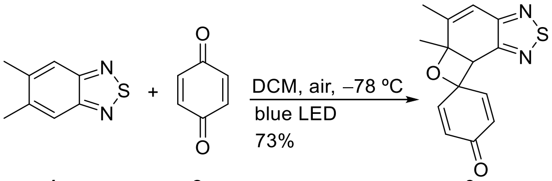

1a **2a** **3a**

To a quartz tube containing a stirring bar was added **1a** (148 mg, 0.9 mmol), quinone **2** (33 mg, 0.3 mmol), and dichloromethane (3 mL). The reaction tube was stirred for 48 h at –78 °C in air under blue LED (λ = 450 nm, 5 W) irradiation. The reaction mixture was concentrated *in vacuo* and separated by column chromatography (PE/EtOAc = 5:1) to obtain **3** (61 mg, 73%).

Energy transfer calculations:

Using the Gibbs energy of PET equation:

$\Delta G = E(D) - E_{red}(A) - \Delta E_{exc} + \Delta E_{coul}$

ΔG of PET process between *p*-benzoquinone (**2a**) and **1a** has been calculated. E_{ox}(D) is the oxidation potential of the donor **1a**, E_{ox}(D) = 1.64 V, which was detected by the cyclic voltammetry (Figure 5). E_{red}(A) is the reduction potential of the acceptor **2a** reported E_{red}(**2a**) = – 0.90 V,[2] ΔE_{exc} is the excitation energy of the acceptor **2a**, reported in literature as 2.20 V for triplet state[3] and 2.34 V (from fluorescence)[4] for singlet state. ΔE_{coul} is a term that considers the ability of the solvent to separate the radical-ion pair, its value is reported as 0.05 V for CH_3CN.

ΔG_{PET} = **+0.39** for triplet state of **2a**
ΔG_{PET} = **+0.25** for singlet state of **2a**

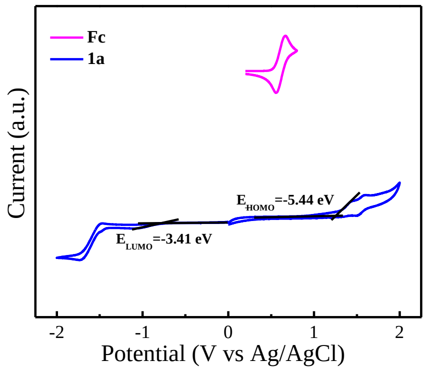

Figure S3. Energy transfer calculations and cyclic voltammetry (CV) curve of **1a** in DCM.

Reference:

[1] a) A. Sharma, V. Dixit, S. Kumar, N. Jain, *Org. Lett.* **2021**, *23*, 3409–3414; b) M. Fréneau, N. Hoffmann, *J. Photochem. Photobiol. C Photochem. Rev.* **2017**, *33*, 83–108.

[2] A. Sagadevan, A. Ragupathi, K. C. Hwang, *Angew. Chem. Int. Ed.* **2015**, *54*, 13896–13901.

[3] K. A. Schnapp, R. M. Wilson, D. M. Ho, R. A. Caldwell, D. Creed, *J. Am. Chem. Soc.* **1990**, *112*, 3700–3702.

[4] J. Yang, J. Duan, G. Wang, H. Zhou, B. Ma, C. Wu, J. Xiao, *Org. Lett.* **2020**, *22*, 7284–7289.

6. DFT calculations data

The structure optimization of ground state of **3a** (opt b3lyp/6-31g(d,p))

Standard orientation:

Center Number	Atomic Number	Atomic Type	Coordinates (Angstroms) X	Y	Z
1	6	0	-1.956826	-1.476825	-0.582601
2	6	0	-0.810873	-1.682135	0.390591
3	6	0	-0.232267	-0.378475	0.986389
4	6	0	-0.972880	0.848706	0.572036
5	6	0	-2.014930	0.855460	-0.276270
6	6	0	-2.482183	-0.266669	-0.838640
7	7	0	-0.614840	1.975340	1.020284
8	16	0	-1.618145	3.188369	0.377308
9	7	0	-2.483112	2.011849	-0.495402
10	6	0	-2.482176	-2.710517	-1.277606
11	8	0	0.391609	-1.968222	-0.285251
12	6	0	0.995833	-0.759056	0.135916
13	6	0	2.238719	-0.997497	0.945088
14	6	0	3.412843	-0.423678	0.646480
15	6	0	3.591095	0.408937	-0.414028
16	6	0	2.526023	0.667455	-1.219240
17	6	0	1.305307	0.151062	-1.017982
18	8	0	4.676912	0.904709	-0.640203
19	6	0	-1.090980	-2.740854	1.453966
20	1	0	-0.060041	-0.436393	2.082918
21	1	0	-3.322293	-0.185358	-1.548510
22	1	0	-2.999109	-3.379701	-0.554492
23	1	0	-3.213640	-2.471533	-2.081112
24	1	0	-1.644615	-3.271584	-1.750238
25	1	0	2.163475	-1.667445	1.818031
26	1	0	4.276552	-0.644711	1.298522
27	1	0	2.667873	1.333627	-2.088771
28	1	0	0.491197	0.393684	-1.720906
29	1	0	-1.255937	-3.744554	1.003069
30	1	0	-0.241118	-2.850496	2.164922
31	1	0	-1.994759	-2.467124	2.044488

Total Energy $E_{(RB3LYP/6\text{-}31G(d))} = -1198.82023663$ A.U.

The structure optimization of ground state of **3a'**(opt b3lyp/6-31g(d,p))

Standard orientation:

Center Number	Atomic Number	Atomic Type	Coordinates (Angstroms) X	Y	Z
1	6	0	0.420120	1.703496	0.064558
2	6	0	-0.110768	0.340944	0.447692
3	6	0	0.568826	-0.502169	-0.652198
4	6	0	2.014667	-0.502505	-0.317850
5	6	0	2.553530	0.714932	-0.095964
6	6	0	1.765418	1.811039	-0.053771
7	7	0	2.785781	-1.498744	-0.370835
8	16	0	4.369560	-0.930399	-0.121836
9	7	0	3.816531	0.676531	-0.021334
10	6	0	-0.501938	2.884214	0.187683
11	6	0	-1.307943	-0.506348	-0.054929
12	8	0	-0.380765	-1.520884	-0.491445
13	6	0	-2.106063	0.131010	-1.164715
14	6	0	-3.444786	0.205096	-1.152367
15	6	0	-4.215689	-0.302644	-0.155142
16	6	0	-3.592818	-0.927258	0.878432
17	6	0	-2.261536	-1.055630	0.971447
18	6	0	0.273734	0.016152	1.904900
19	8	0	-5.426080	-0.207067	-0.190698
20	1	0	0.481058	-0.116659	-1.697306
21	1	0	2.229406	2.810337	-0.068773
22	1	0	-1.356537	2.779856	-0.516902
23	1	0	-0.906192	2.944735	1.223366
24	1	0	0.010604	3.844619	-0.042730
25	1	0	-1.573000	0.576547	-2.020220
26	1	0	-3.943697	0.705920	-2.001352
27	1	0	-4.212955	-1.359054	1.684360
28	1	0	-1.852232	-1.602268	1.835911
29	1	0	1.264945	0.420892	2.204351
30	1	0	-0.468490	0.474938	2.597218
31	1	0	0.324169	-1.076379	2.107122

Total Energy $E_{(RB3LYP/6\text{-}31G(d,p))}$ = −1198.77502142 A.U.

The structure optimization of ground state of **1a** (opt b3lyp/6-31g(d,p))

Standard orientation:

Center Number	Atomic Number	Atomic Type	Coordinates (Angstroms) X	Y	Z
1	6	0	0.704700	0.753984	-0.000072
2	6	0	0.704637	-0.754027	-0.000008
3	6	0	-0.511119	-1.478424	0.000032
4	6	0	-1.688606	-0.708086	-0.000001
5	6	0	-1.688527	0.708076	-0.000011
6	6	0	-0.511189	1.478464	-0.000094
7	7	0	1.938167	1.282400	-0.000057
8	16	0	2.995614	-0.000001	0.000048
9	7	0	1.938212	-1.282400	0.000008
10	6	0	-3.005363	1.420161	0.000094
11	6	0	-3.005427	-1.420149	-0.000056
12	1	0	-0.534269	-2.578152	0.000070
13	1	0	-0.534340	2.578229	-0.000103
14	1	0	-3.831196	0.673981	0.000154
15	1	0	-3.081868	2.057425	0.909320
16	1	0	-3.082064	2.058040	-0.908718
17	1	0	-3.831320	-0.673999	-0.000001
18	1	0	-3.081972	-2.057465	-0.909276
19	1	0	-3.082085	-2.058040	0.908816

Total Energy $E_{(RB3LYP/6\text{-}31G(d,p))} = -817.35827844$ A.U.

The structure optimization of triplet state of **1a** (opt ub3lyp/6-31g(d,p))

Standard orientation:

Center Number	Atomic Number	Atomic Type	Coordinates (Angstroms) X	Y	Z
1	6	0	0.713835	0.723230	-0.000005
2	6	0	0.713835	-0.723230	-0.000031
3	6	0	-0.521900	-1.420158	-0.000020
4	6	0	-1.712770	-0.726961	0.000016
5	6	0	-1.712770	0.726960	0.000006
6	6	0	-0.521899	1.420157	0.000004
7	7	0	1.940054	1.265421	-0.000043
8	16	0	2.992730	0.000000	0.000063
9	7	0	1.940054	-1.265420	-0.000104
10	6	0	-2.988910	1.547653	-0.000016
11	6	0	-2.988910	-1.547653	0.000043
12	1	0	-0.510858	-2.505728	-0.000033
13	1	0	-0.510859	2.505727	-0.000016
14	1	0	-3.897654	0.947944	-0.000553
15	1	0	-3.027884	2.197266	0.880935
16	1	0	-3.027348	2.198008	-0.880439
17	1	0	-3.897653	-0.947942	0.000600
18	1	0	-3.027898	-2.197252	-0.880918
19	1	0	-3.027337	-2.198021	0.880456

Total Energy $E_{(UB3LYP/6\text{-}31G(d,p))} = -817.28488167$ A.U.
